# Supplementary figures and images for: Wnt target gene activation requires β-catenin separation into biomolecular condensates
Source: PLoS Biol. 2024 Sep 24;22(9):e3002368. doi: 10.1371/journal.pbio.3002368 (PMC11460698; doi:10.1371/journal.pbio.3002368)

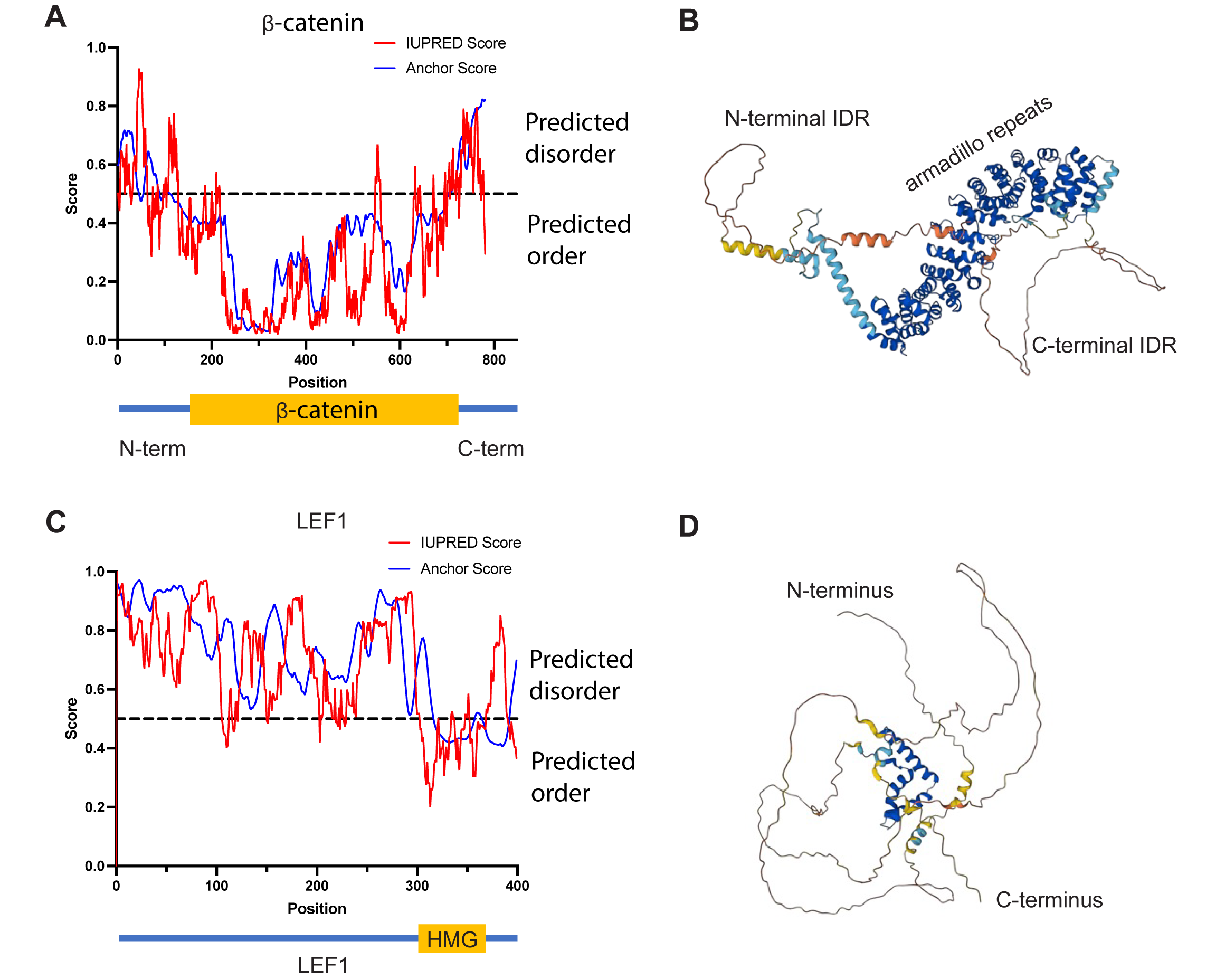

Supplement: S1 Fig — (A) IUPRED and Anchor analysis of human β-catenin. Regions with scores above 0.5 are predicted to be disordered; scores below 0.5 are predicted to be ordered. (B) AlphaFold prediction of β-catenin structure. The N- and C-termini lack a predicted structure, while the armadillo repeat region is α-helix rich and highly structured. (C) IUPRED and Anchor analysis of human LEF1. (D) AlphaFold prediction of LEF1 structure, indicating a mostly disordered structure. Summary data displayed in S1 Fig can be found in S1 Data. (TIF) [file pbio.3002368.s001.tif]

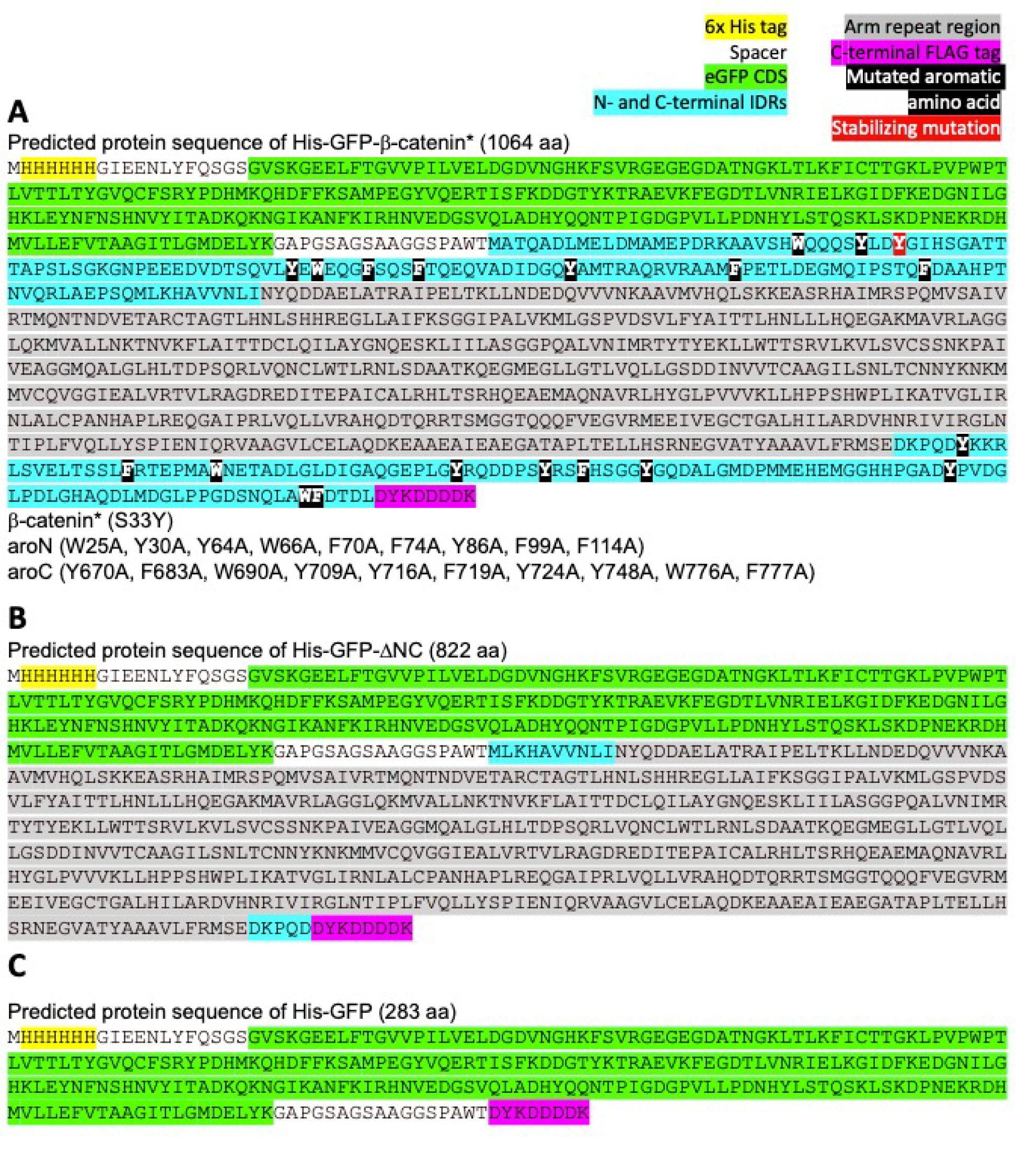

Supplement: S2 Fig — (A) Annotated amino acid sequences of recombinantly expressed His-eGFP-β-catenin* protein and its aromatic mutant derivatives. The specific amino acid residues that were mutated to create β-catenin*, aroN, and aroC are listed below the annotated sequence. AroNC contains all aroN and aroC mutations. (B) Annotated amino acid sequence of the His-eGFP-ΔNC mutant. (C) Annotated amino acid sequence of the His-eGFP mutant. (TIF) [file pbio.3002368.s002.tif]

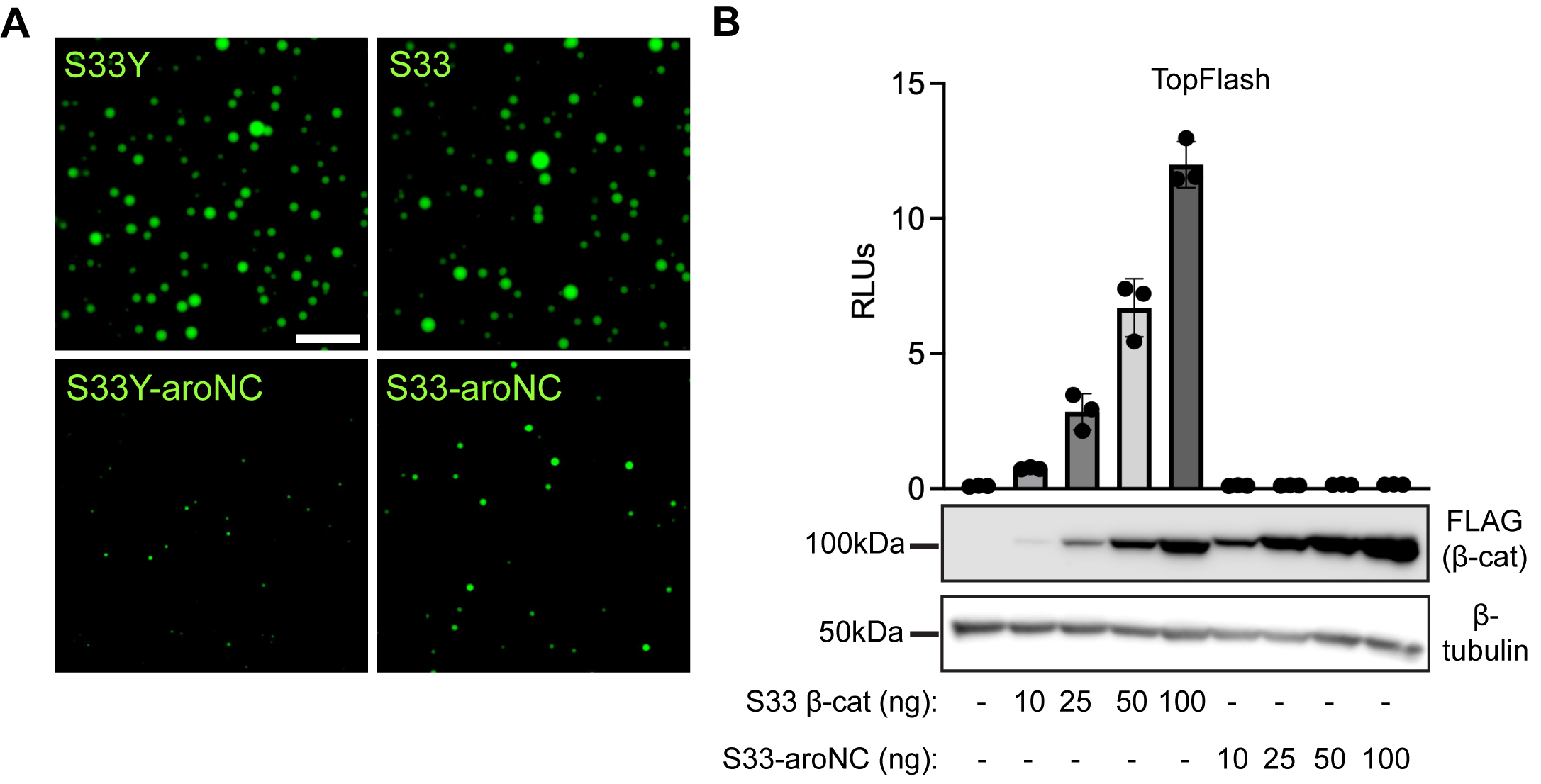

Supplement: S3 Fig — (A) Representative images from an in vitro droplet formation assay with the indicated mutants. Droplet assays were performed in 300 mM NaCl and 10% PEG-8000. Scale bar = 20 μm. (B) TopFlash luciferase reporter activity induced by S33-β-catenin (WT) or S33-aroNC (WT) constructs in HEK293T cells. Cells were transiently transfected with separate plasmids encoding the reporter genes and the β-catenin mutant constructs. Corresponding western blots show relative expression of the FLAG-β-catenin mutant constructs. Summary data displayed in S2 Fig can be found in S1 Data (TIF) [file pbio.3002368.s003.tif]

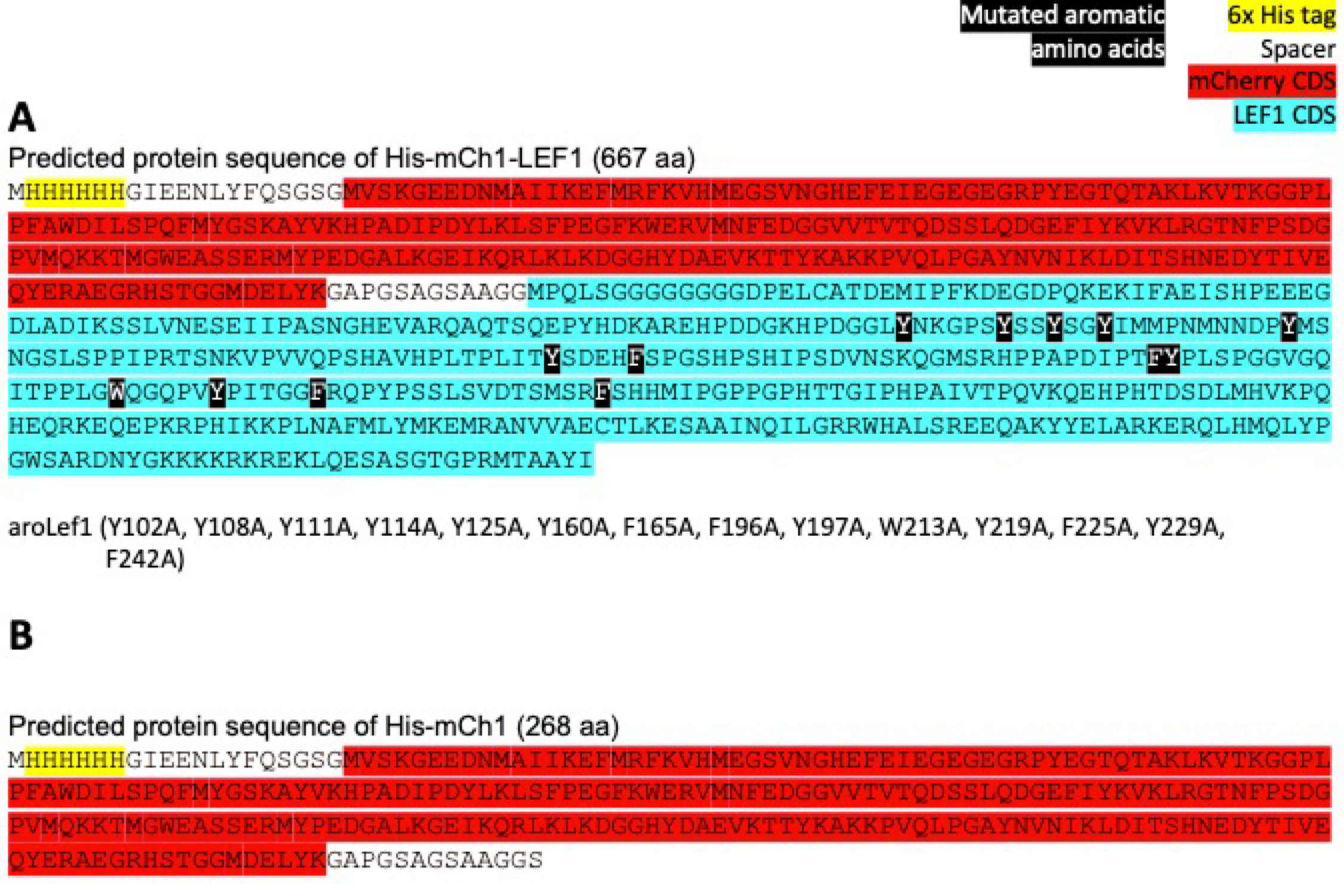

Supplement: S4 Fig — (A) Annotated amino acid sequence of recombinantly expressed His-mCherry-LEF1 protein. Aromatic residues that were changed to alanines in LEF1aro are highlighted in black. (B) Annotated amino acid sequence of His-mCherry. (TIF) [file pbio.3002368.s004.tif]

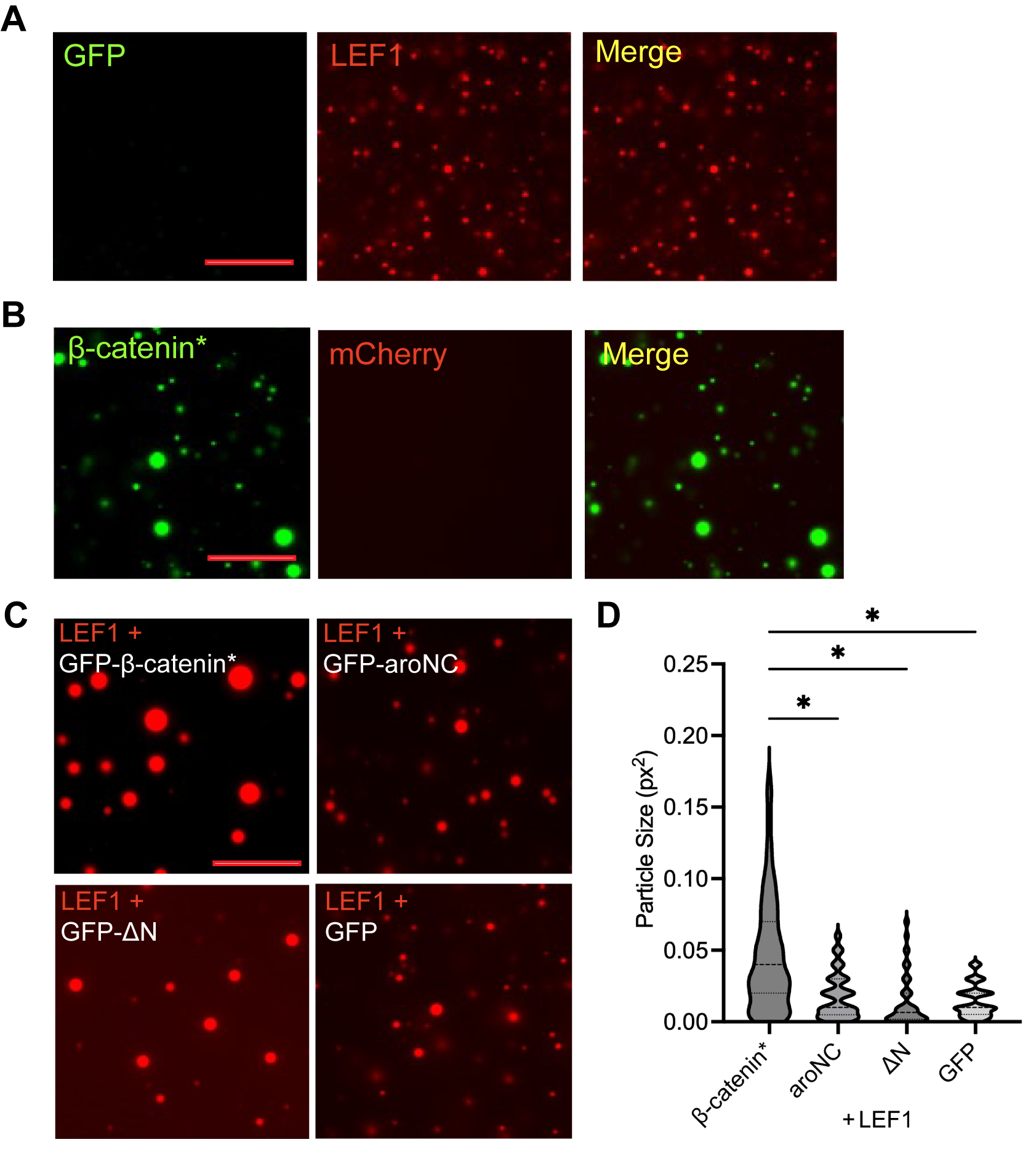

Supplement: S5 Fig — (A) Representative images from a heterotypic in vitro droplet formation assay with eGFP and mCherry-LEF1. The concentration of both eGFP and LEF1 protein is 8 μm. Droplet assays were performed in 300 mM NaCl and 10% PEG-8000. Scale bar = 20 μm. (B) Representative images from a heterotypic in vitro droplet formation assay with eGFP-β-catenin* and mCherry. The concentration of both eGFP-β-catenin* and mCherry protein is 8 μm. Droplet assays were performed in 300 mM NaCl and 10% PEG-8000. Scale bar = 20 μm. (C) Representative images from a heterotypic in vitro droplet formation assay with mCherry-LEF1 and the indicated GFP-β-catenin mutants. The concentration of both proteins is 8 μm. Images depict mCherry-LEF1 fluorescence only. Scale bar = 20 μm. (D) Violin plots depicting quantification of individual particle sizes. For β-catenin*, n = 336, for aroN, n = 624, for aroC, n = 490, and for aroNC, n = 900; p-values were calculated using one-way ANOVA followed by Dunnett’s test. * = p < 0.05. Summary data displayed in S5 Fig can be found in S1 Data. (TIF) [file pbio.3002368.s005.tif]

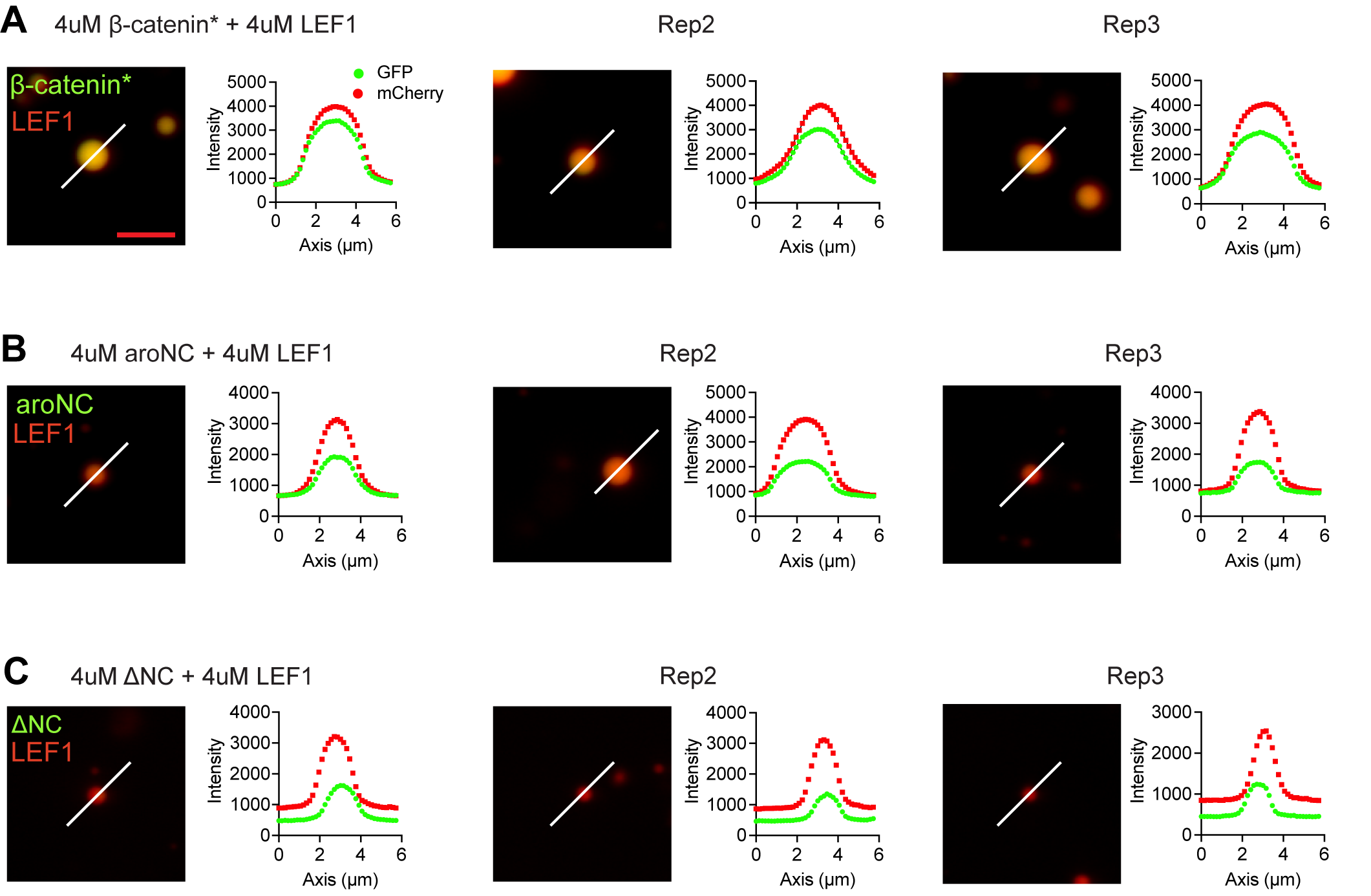

Supplement: S6 Fig — (A) Triplicate line plots showing eGFP-β-catenin* + mCherry-LEF1. (B) Triplicate line plots showing eGFP-aroNC and mCherry-LEF1. (C) Triplicate line plots showing eGFP-ΔNC and mCherry-LEF1. White lines represent the plotted trace. Scale bar = 5 μm. Summary data displayed in S6 Fig can be found in S1 Data. (TIF) [file pbio.3002368.s006.tif]

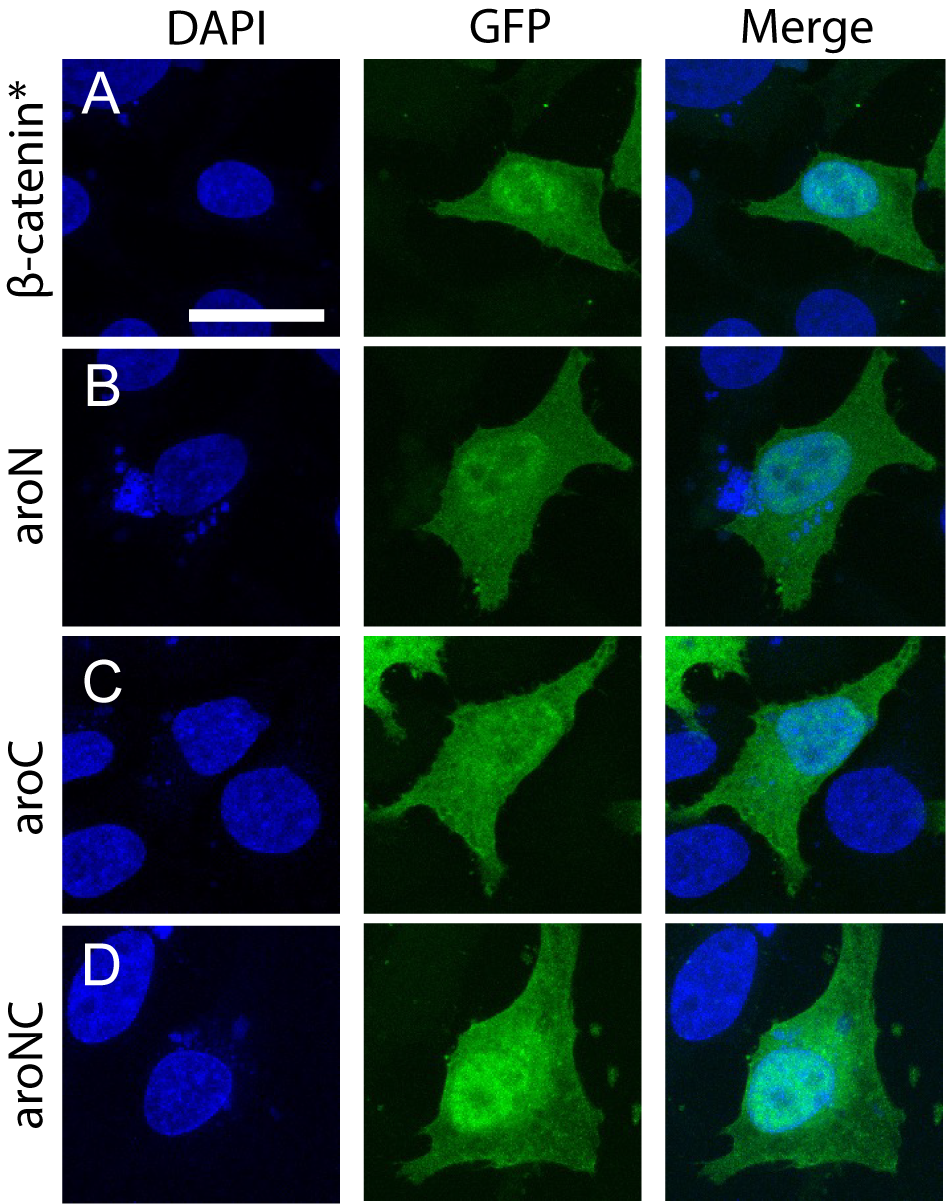

Supplement: S7 Fig — (A) Representation of diffuse eGFP-β-catenin* signal, (B) aroN, (C) aroC, and (D) aroNC in HEK293T cells. Scale bar = 20 μm. (TIF) [file pbio.3002368.s007.tif]

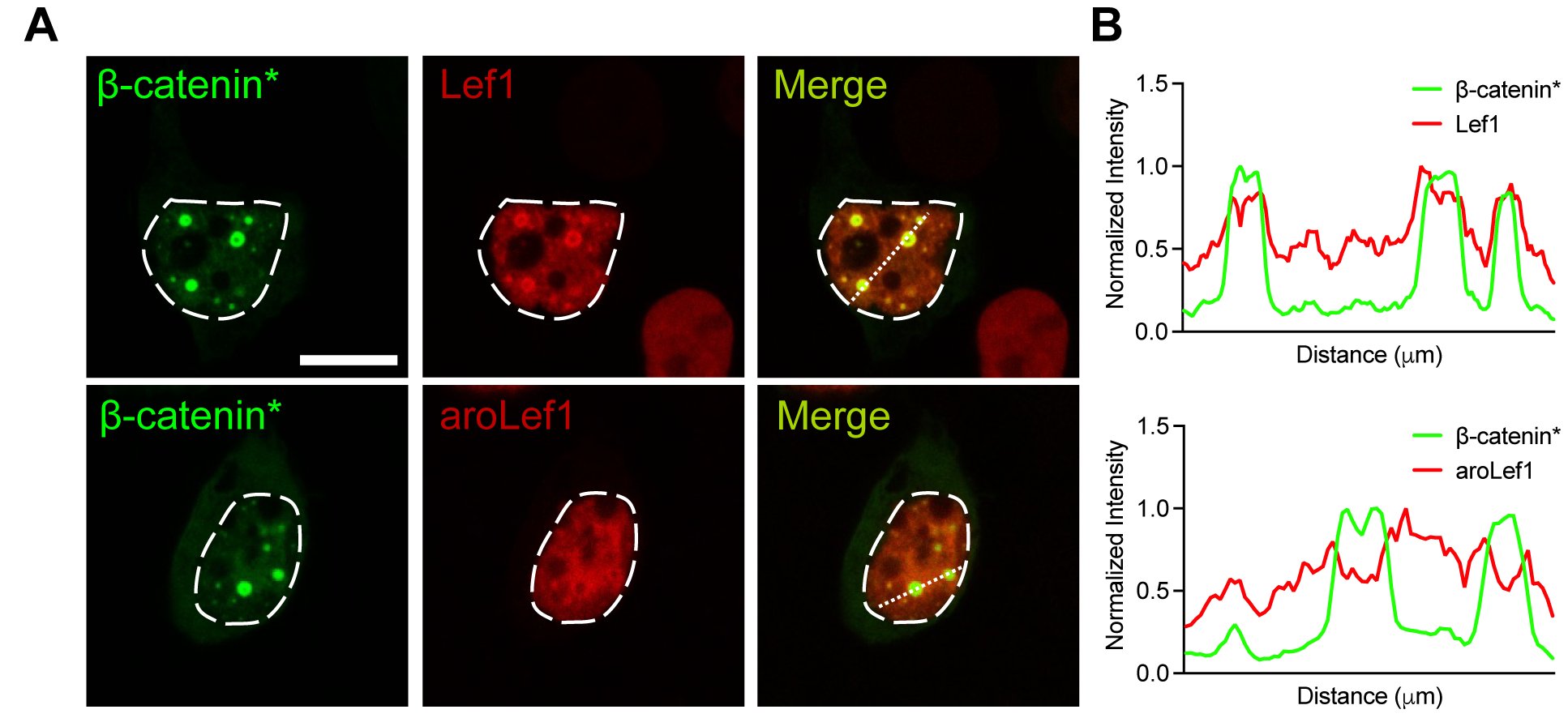

Supplement: S8 Fig — (A) Representative single slice confocal images of HEK293T β-catenin KO cells overexpressing the indicated proteins. Scale bar = 20 μm. (B) Quantification of line plots showing the colocalization of eGFP-β-catenin* and mCherry-LEF1. Summary data displayed in S8 Fig can be found in S1 Data. (TIF) [file pbio.3002368.s008.tif]

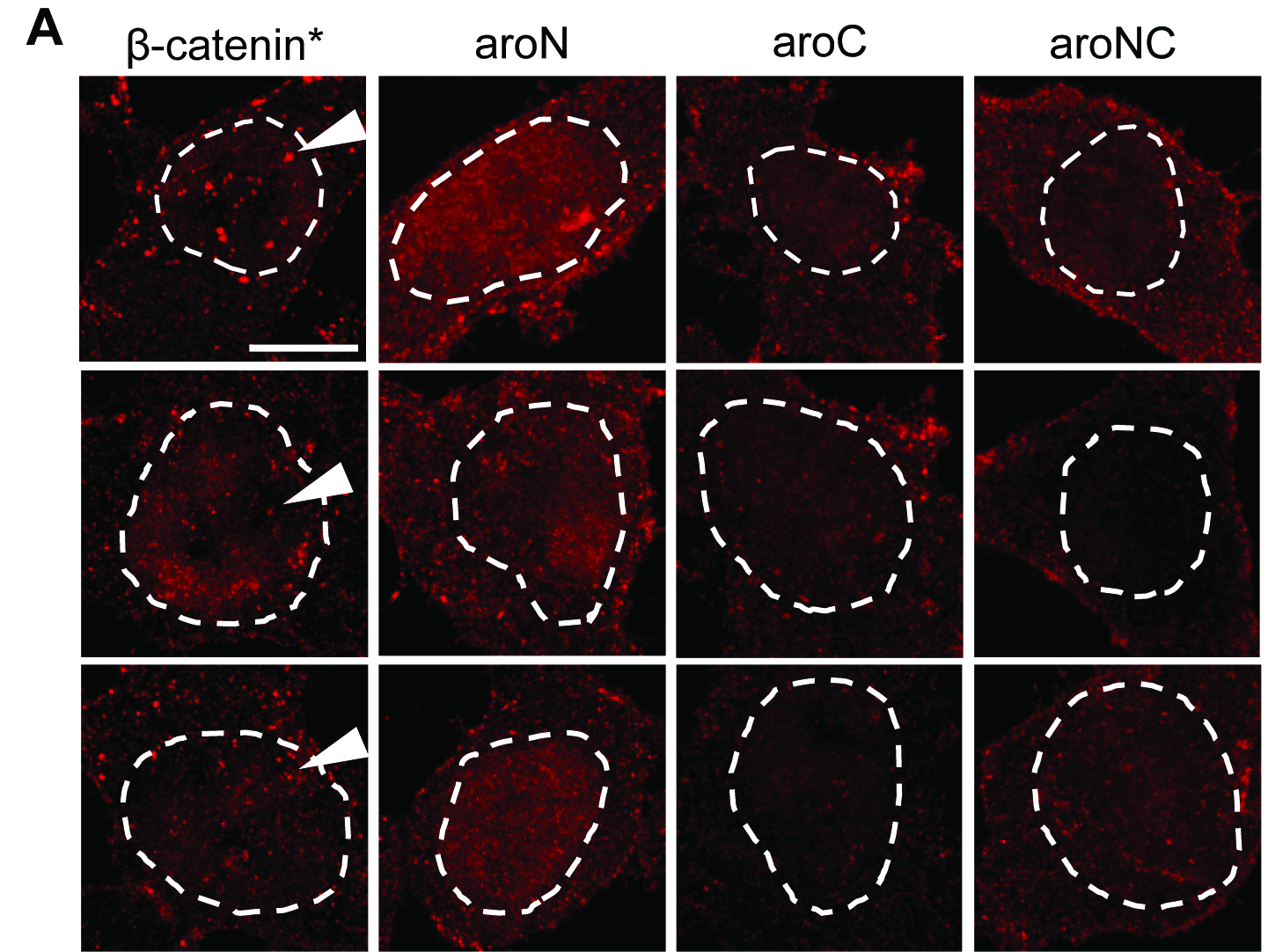

Supplement: S9 Fig — (A) Representative immunofluorescence images of in vivo puncta formed by the indicated FLAG-β-catenin constructs. White arrows indicate puncta. Scale bar = 10 μm. (TIF) [file pbio.3002368.s009.tif]

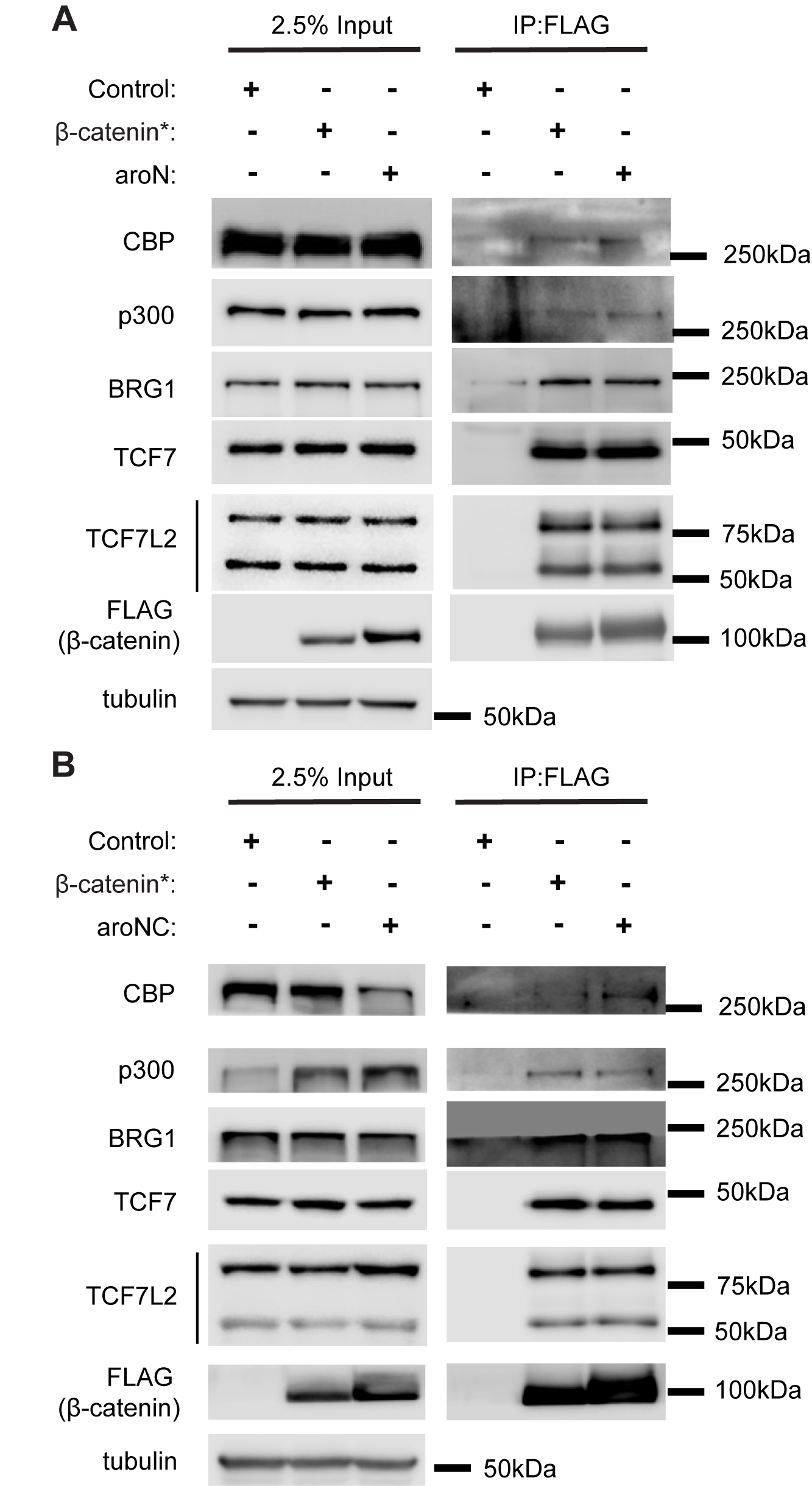

Supplement: S10 Fig — (A) FLAG-β-catenin* and FLAG-aroN immunoprecipitates known, transcriptionally relevant binding partners. Left: total protein lysate from HEK293T β-catenin KO cells overexpressing either FLAG-β-catenin* or FLAG-aroN was used in the immunoprecipitation. Right: elution fractions were run on a gel and blotted for the indicated proteins. (B) Similar immunoprecipitation using total protein lysate from HEK293T β-catenin KO cells overexpressing either FLAG-β-catenin* or FLAG-aroNC. (TIF) [file pbio.3002368.s010.tif]

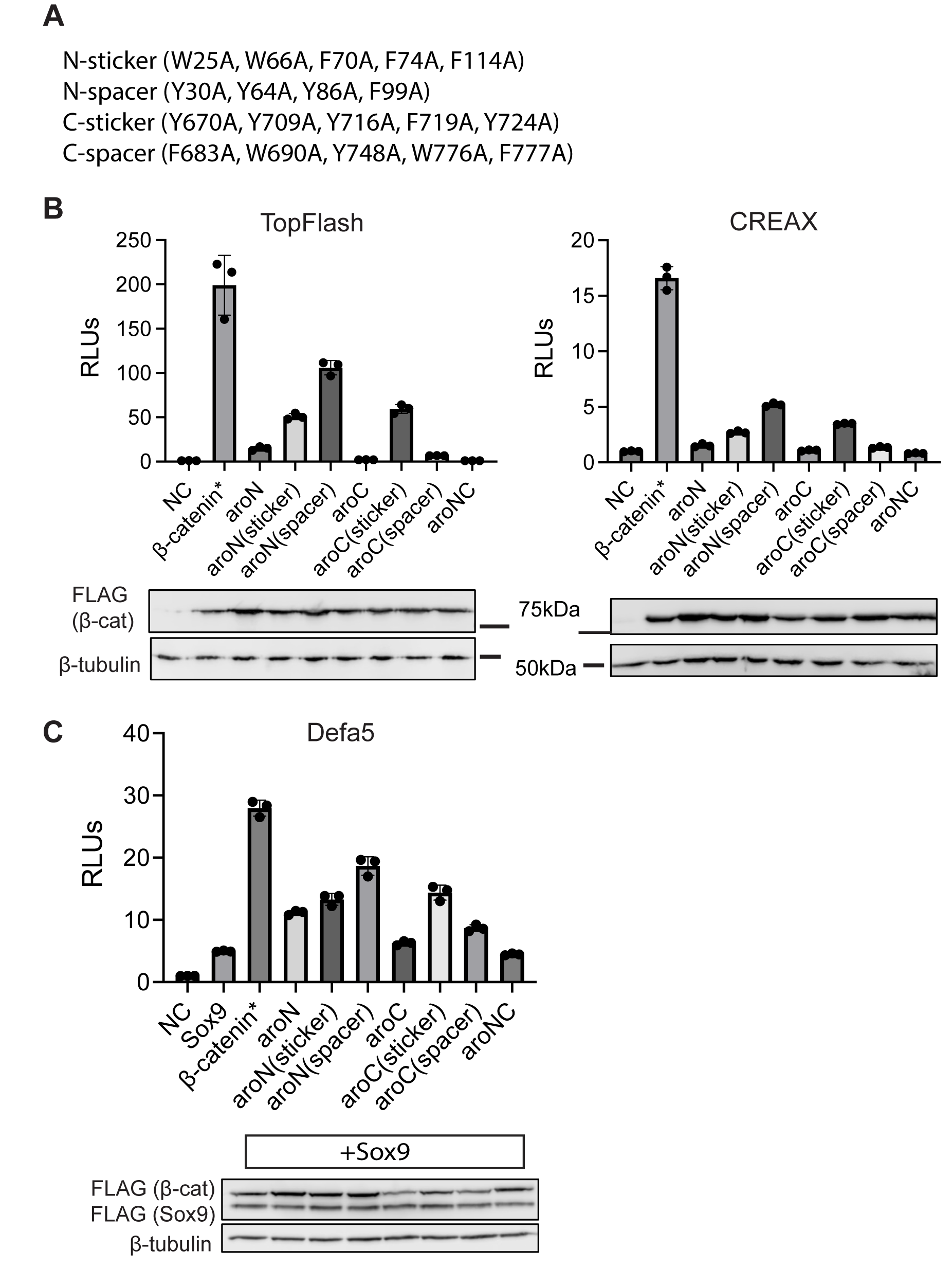

Supplement: S11 Fig — (A) Indications of the specific amino acid residues that were mutated for each construct. (B) Top: TopFlash (left) or CREAX (right) luciferase reporter activity induced by various sticker/spacer β-catenin mutants. Bottom: western blots showing the expression of each mutant construct. The protein samples used for the western blot correspond to the samples used for the luciferase assay. (C) Top: HD5 luciferase reporter activity induced by the sticker/spacer mutants. Bottom: corresponding western blots. Summary data displayed in S11 Fig can be found in S1 Data. (TIF) [file pbio.3002368.s011.tif]

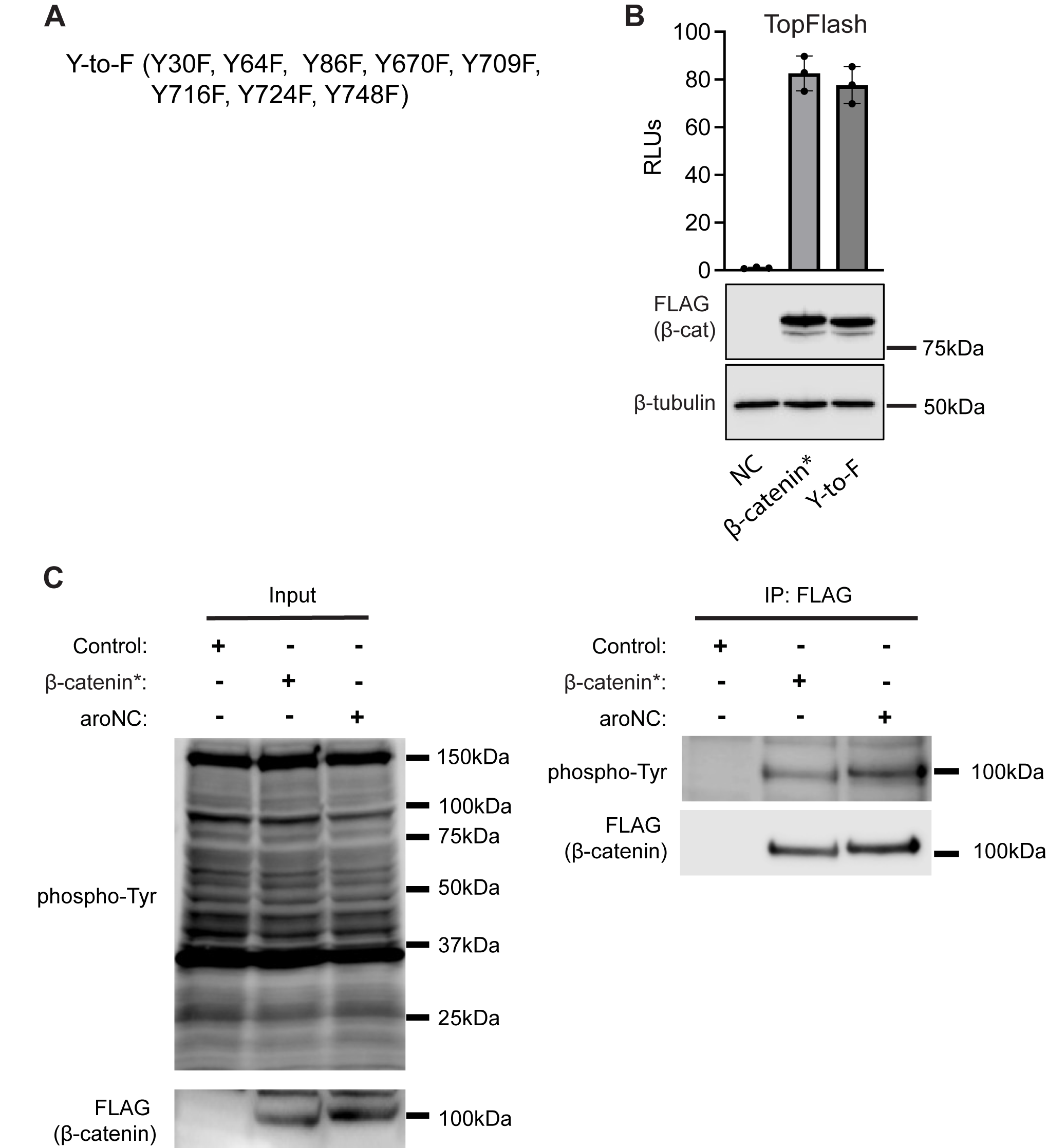

Supplement: S12 Fig — (A) Indications of the specific amino acid residues that were mutated for the Y-to-F construct. (B) Top: TopFlash luciferase reporter data induced by β-catenin* and the Y-to-F β-catenin mutant, which has all tyrosine residues in the terminal IDRs mutated to alanines. Bottom: western blots indicating the corresponding β-catenin mutant expression. (C) FLAG-β-catenin* and FLAG-aroNC immunoprecipitates tyrosine phosphorylated β-catenin. Left: total protein lysate from HEK293T β-catenin KO cells overexpressing either FLAG-β-catenin* or FLAG-aroN was used in the immunoprecipitation. Right: elution fractions were run on a gel and blotted for the tyrosine phosphorylation. Summary data displayed in S12 Fig can be found in S1 Data. (TIF) [file pbio.3002368.s012.tif]

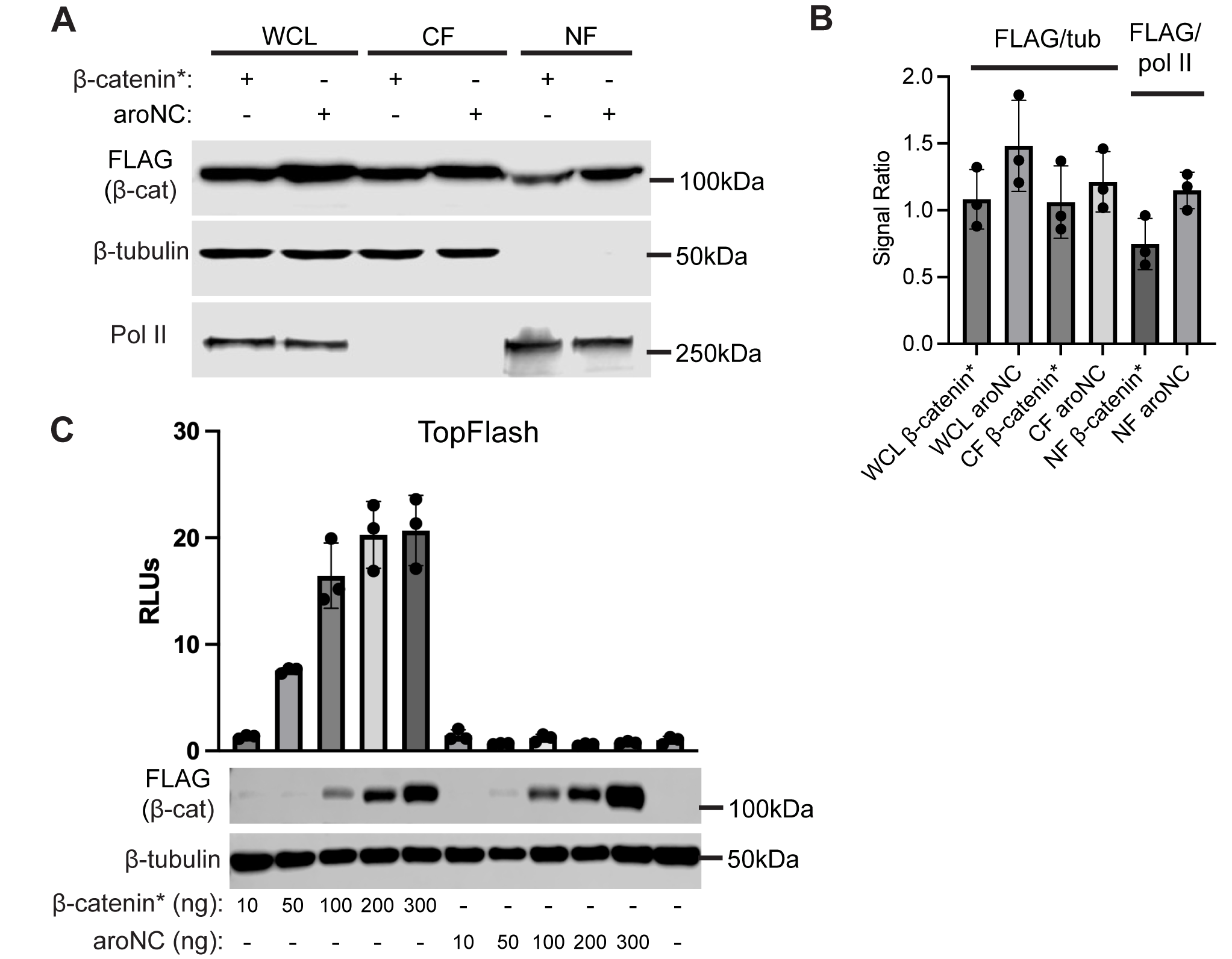

Supplement: S13 Fig — (A) Western blot showing nuclear fractionation samples from HeLa cells expressing FLAG-β-catenin* or FLAG-aroNC. (B) Quantification of western blots from 3 independent experiments. Data presented as the ratio of FLAG signal intensity to either β-tubulin or RNA pol II signal intensity. (C) Top: TopFlash luciferase reporter data induced by a dose of FLAG-β-catenin* or FLAG-aroNC expressed in HeLa cells. Bottom: western blots indicating the corresponding β-catenin mutant expression. Summary data displayed in S13 Fig can be found in S1 Data. (TIF) [file pbio.3002368.s013.tif]

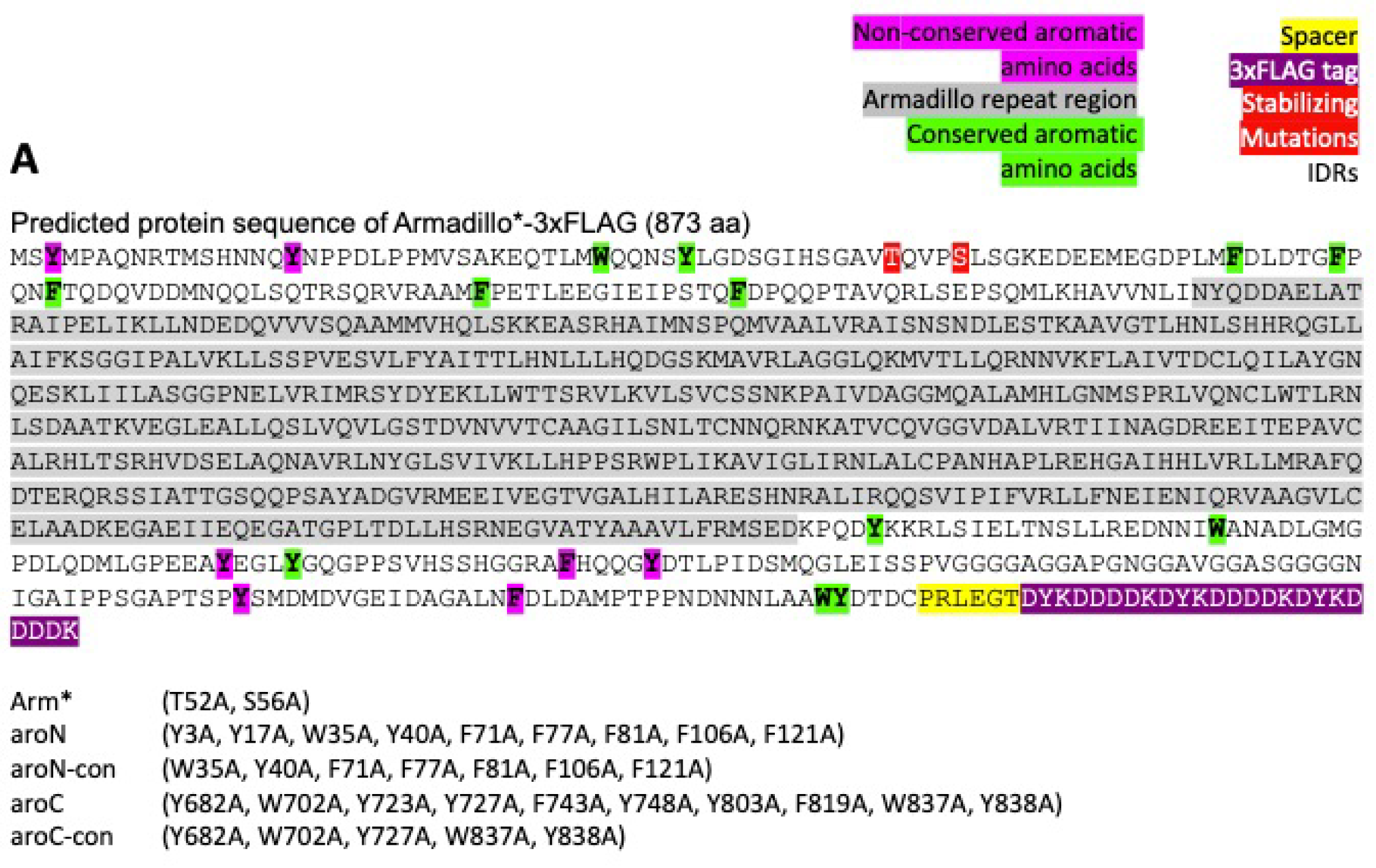

Supplement: S14 Fig — (A) Annotated amino acid sequence of the Arm protein. All the aromatic mutants are built upon the Arm* mutation, i.e., they all have T52A and S56A. (TIF) [file pbio.3002368.s014.tif]

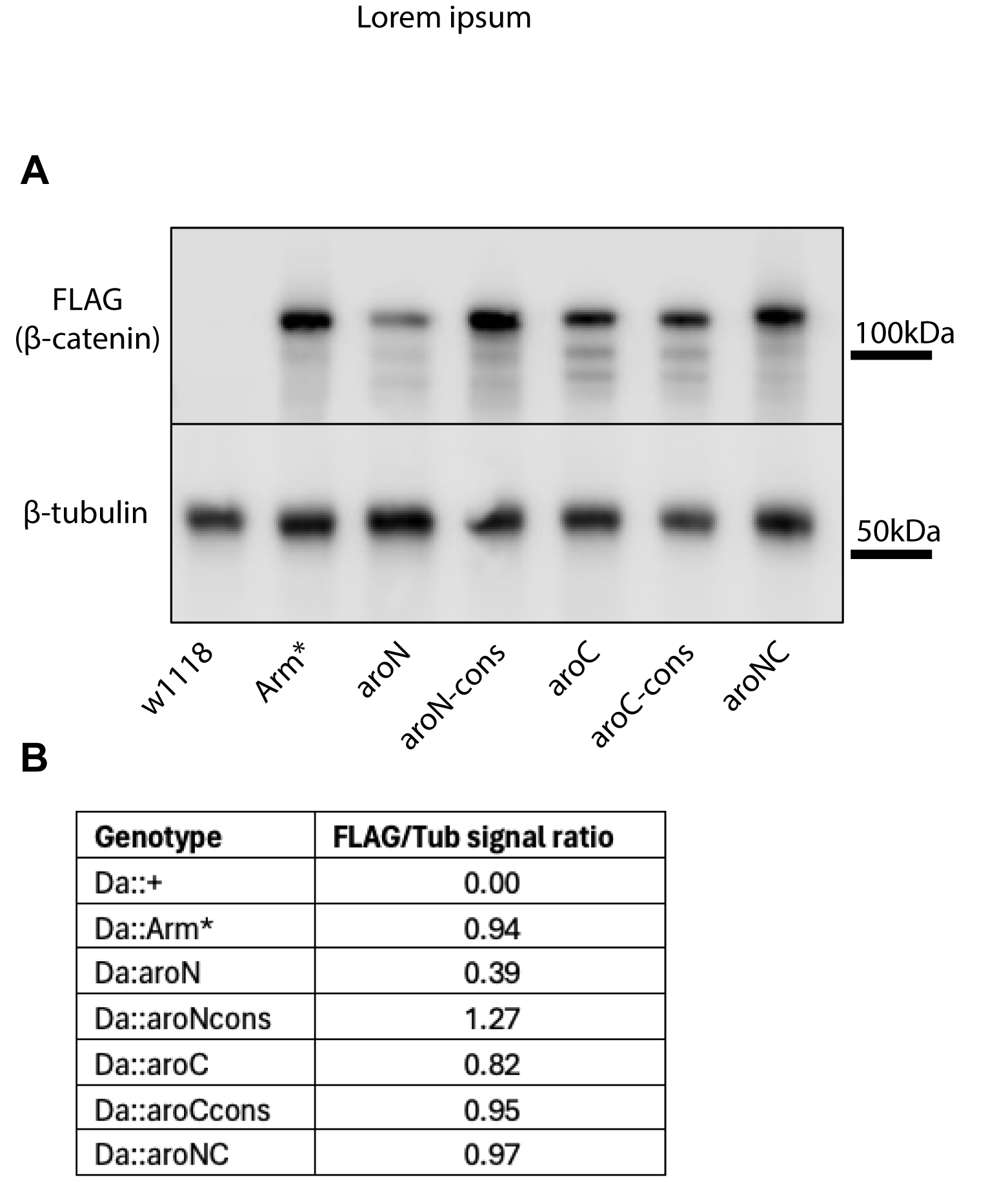

Supplement: S15 Fig — (A) Western blot analysis of Drosophila embryo lysates that were collected 4–8 h after laying. α-FLAG blot shows Arm protein expression and β-tubulin was used as a loading control. (B) Quantification of the ratio of the FLAG to tubulin signal intensity. (TIF) [file pbio.3002368.s015.tif]

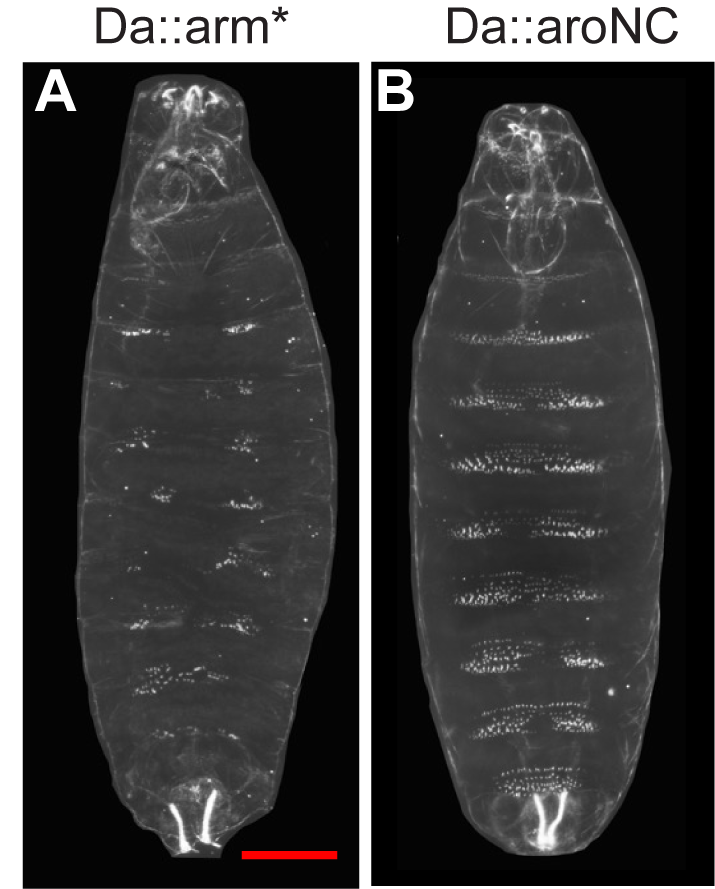

Supplement: S16 Fig — (A) Representative darkfield image showing the ventral side of late embryonic Drosophila cuticles containing the P[Da-Gal4] and P[UAS-Arm*] transgenes. (B) A late embryonic Drosophila cuticle containing the P[Da-Gal4] and P[UAS-aroNC] transgenes. (TIF) [file pbio.3002368.s016.tif]

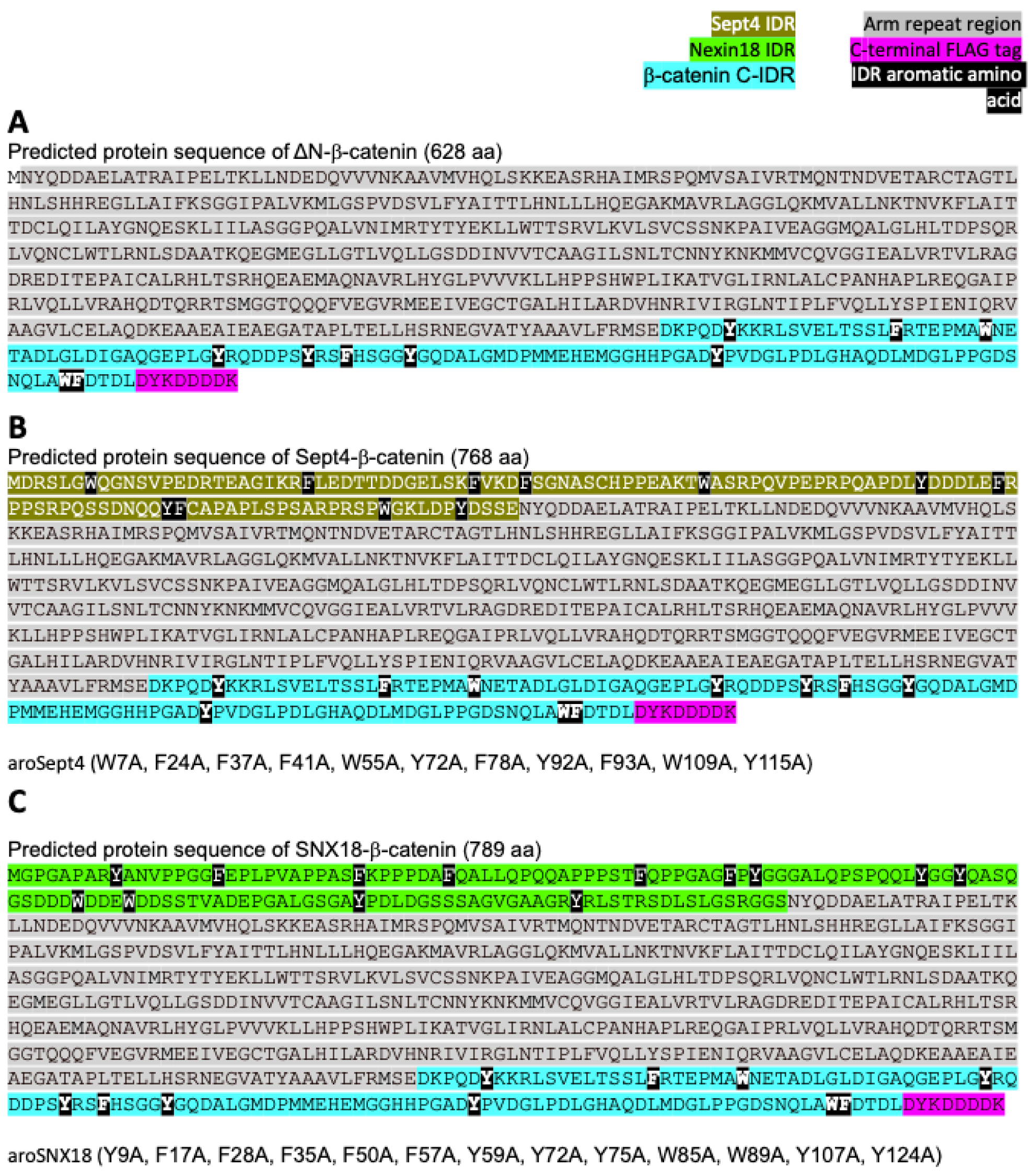

Supplement: S17 Fig — (A) Annotated sequence of Sept4-β-catenin with indicated mutations for aroSept4-β-catenin. (B) Annotated sequence of Nex18-β-catenin. (TIF) [file pbio.3002368.s017.tif]

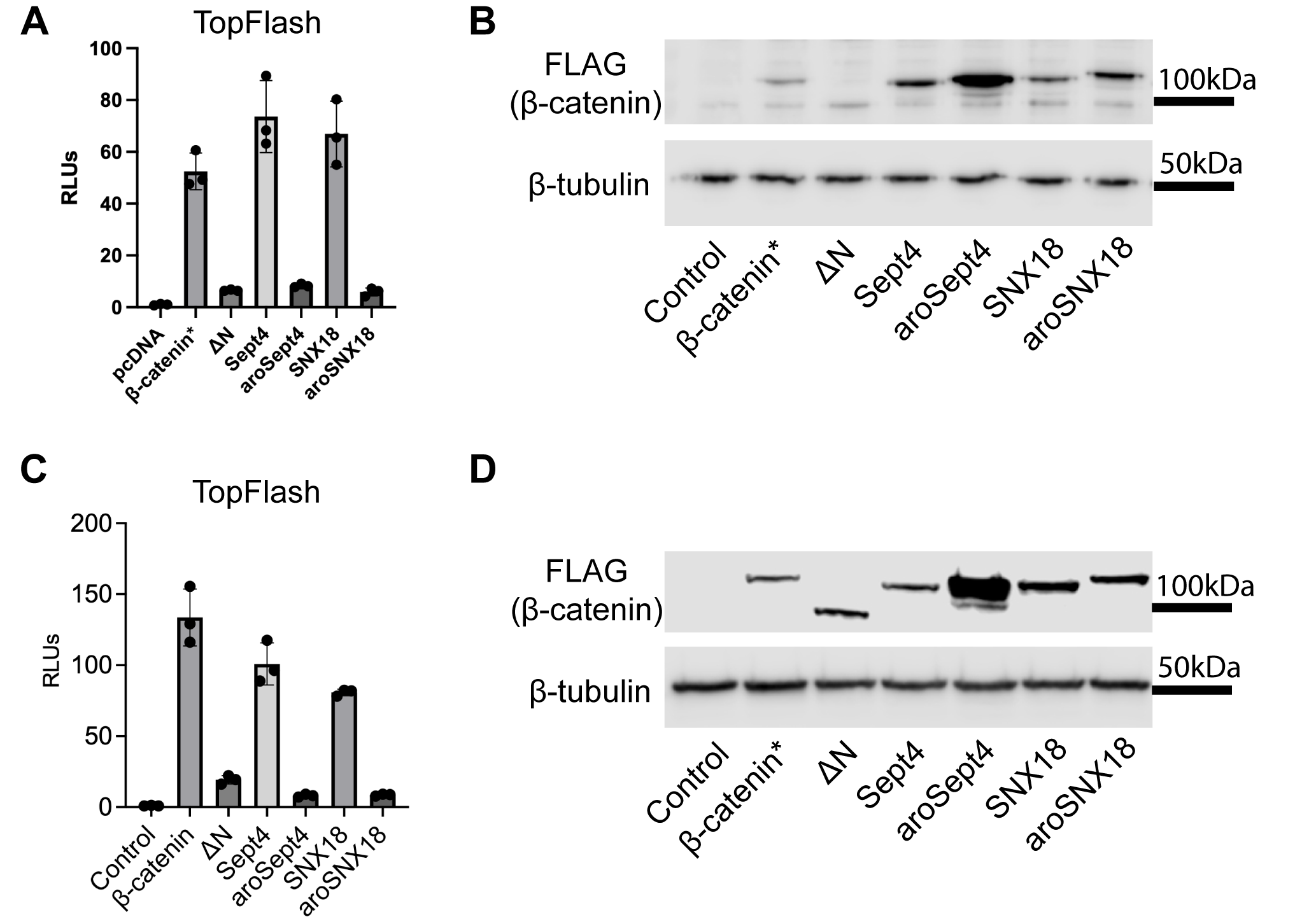

Supplement: S18 Fig — (A) TopFlash luciferase reporter data induced by β-catenin* and the indicated heterologous IDR mutants. (B) Western blot indicating the expression of the various FLAG-tagged β-catenin constructs, data corresponds to panel A. Note: aroSept4 and aroSNX18 are overexpressed relative to their WT counterparts. (C) Additional replicate of TopFlash luciferase reporter data induced by β-catenin* and the indicated heterologous IDR mutants. (D) Western blot indicating the expression of the various FLAG-tagged β-catenin constructs, data corresponds to panel C. Note: ΔN is slightly overexpressed relative to β-catenin*. Summary data displayed in S18 Fig can be found in S1 Data. (TIF) [file pbio.3002368.s018.tif]

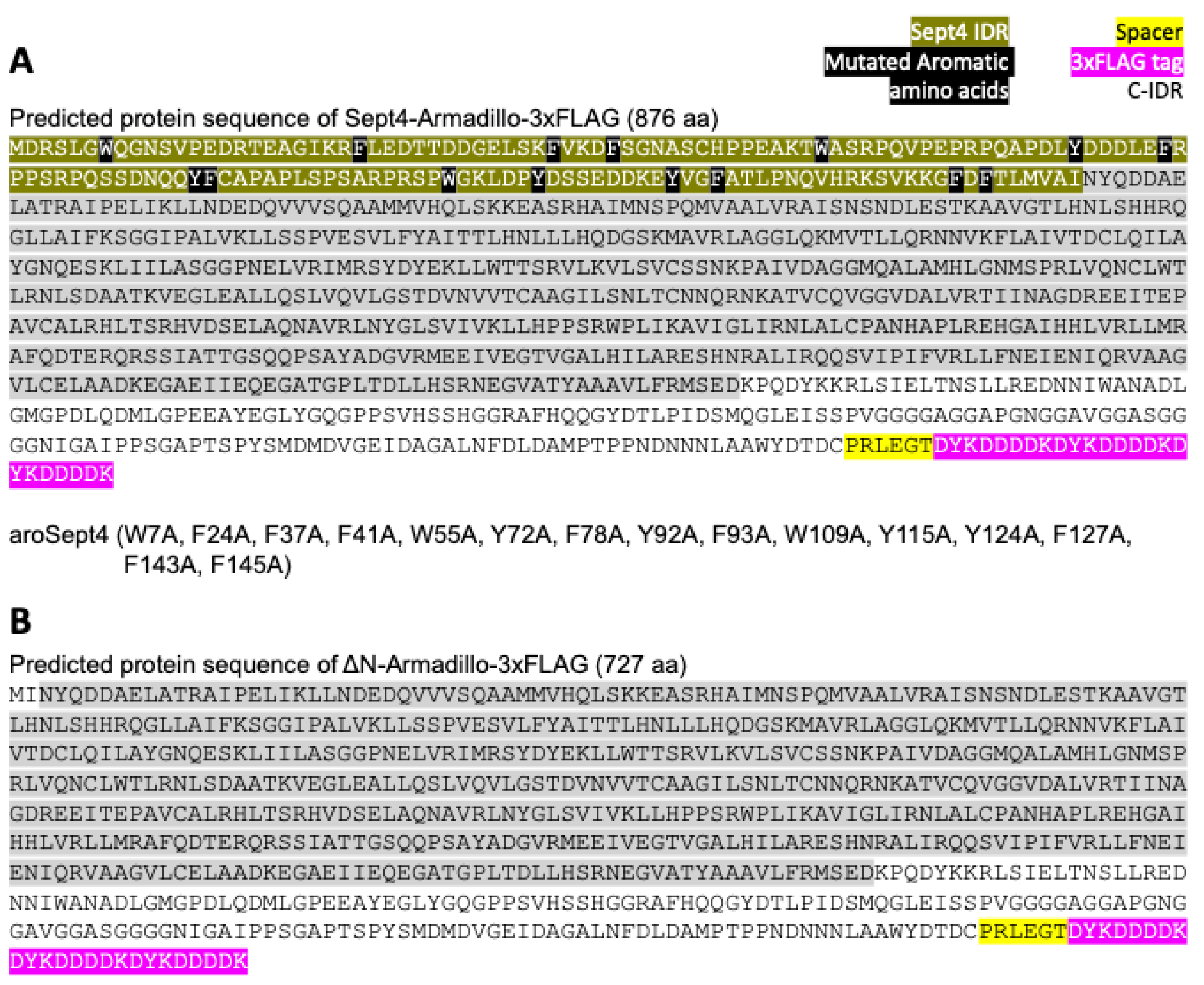

Supplement: S19 Fig — (A) Annotated sequence of Sept4-Armadillo with indicated mutations for aroSept4-Armadillo. (B) Annotated sequence of ΔN-Armadillo. (TIF) [file pbio.3002368.s019.tif]
