# Supplementary material for: Wnt target gene activation requires β-catenin separation into biomolecular condensates
Source: PLoS Biol. 2024 Sep 24;22(9):e3002368. doi: 10.1371/journal.pbio.3002368 (PMC11460698; doi:10.1371/journal.pbio.3002368)
Supplement: S1 Raw images — (PDF) [file pbio.3002368.s022.pdf]

Figure 4A (Left)

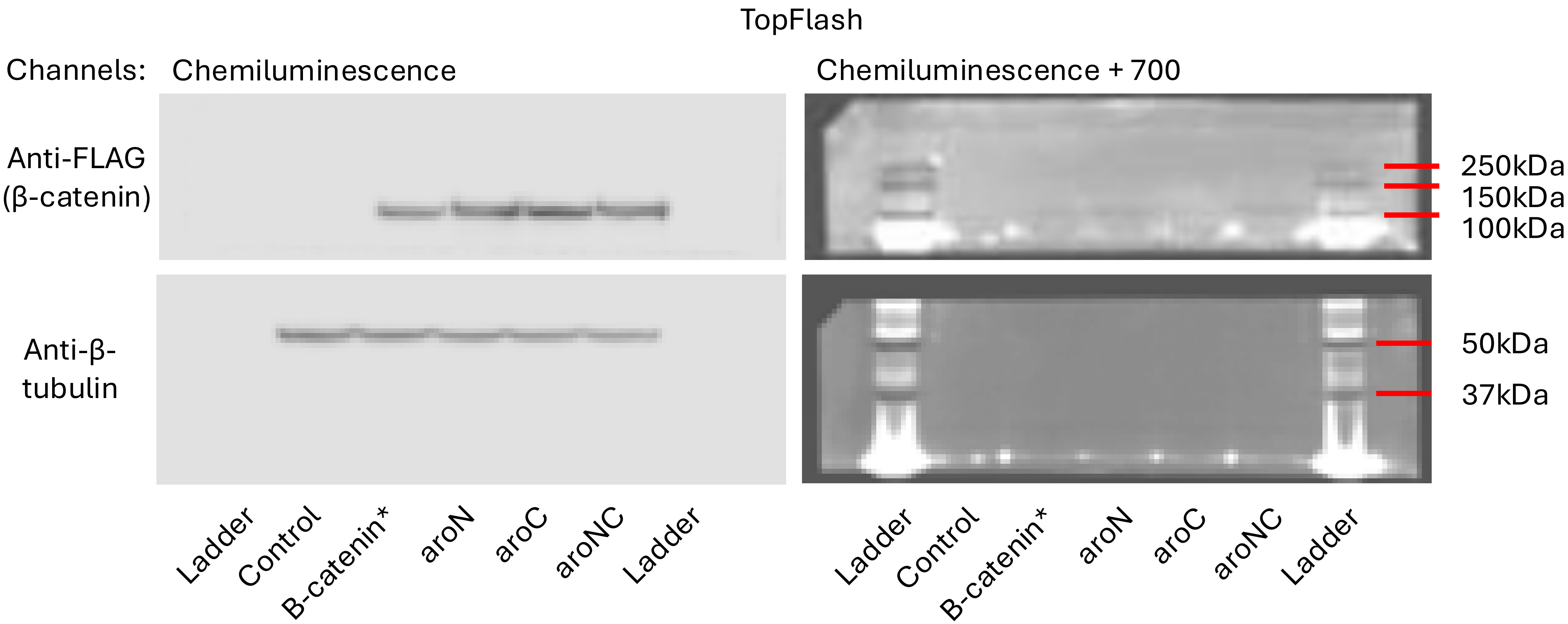

Figure 4A (Right)

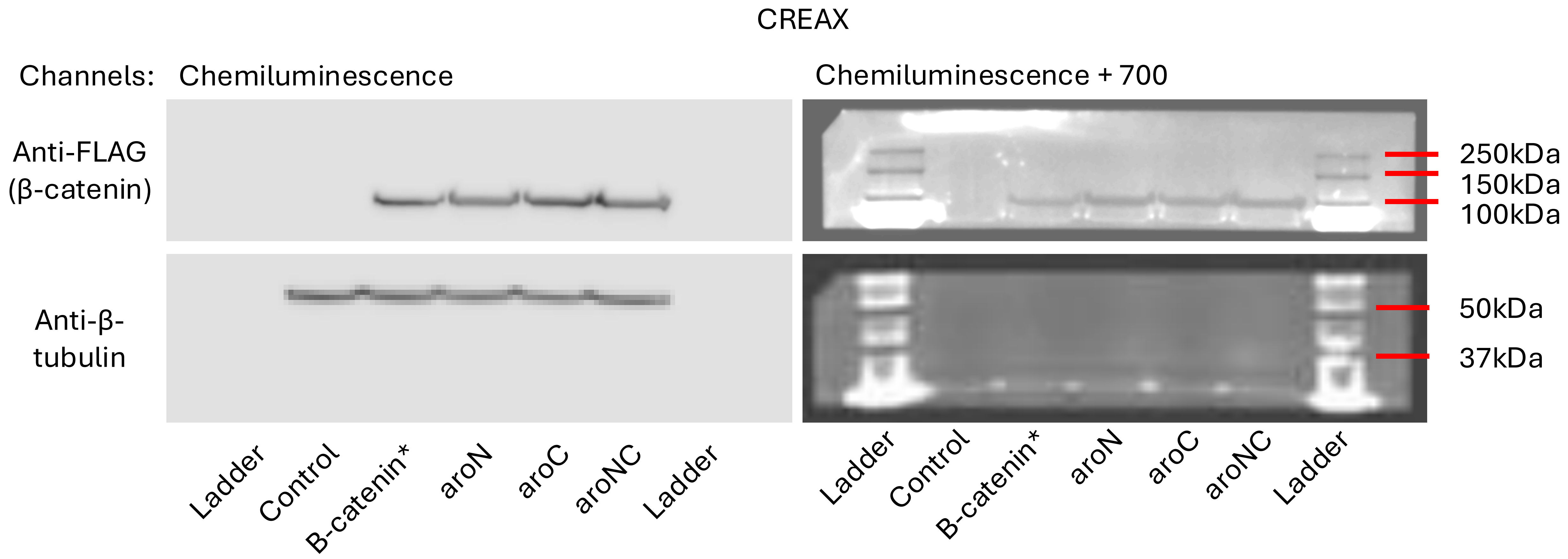

Figure 4E

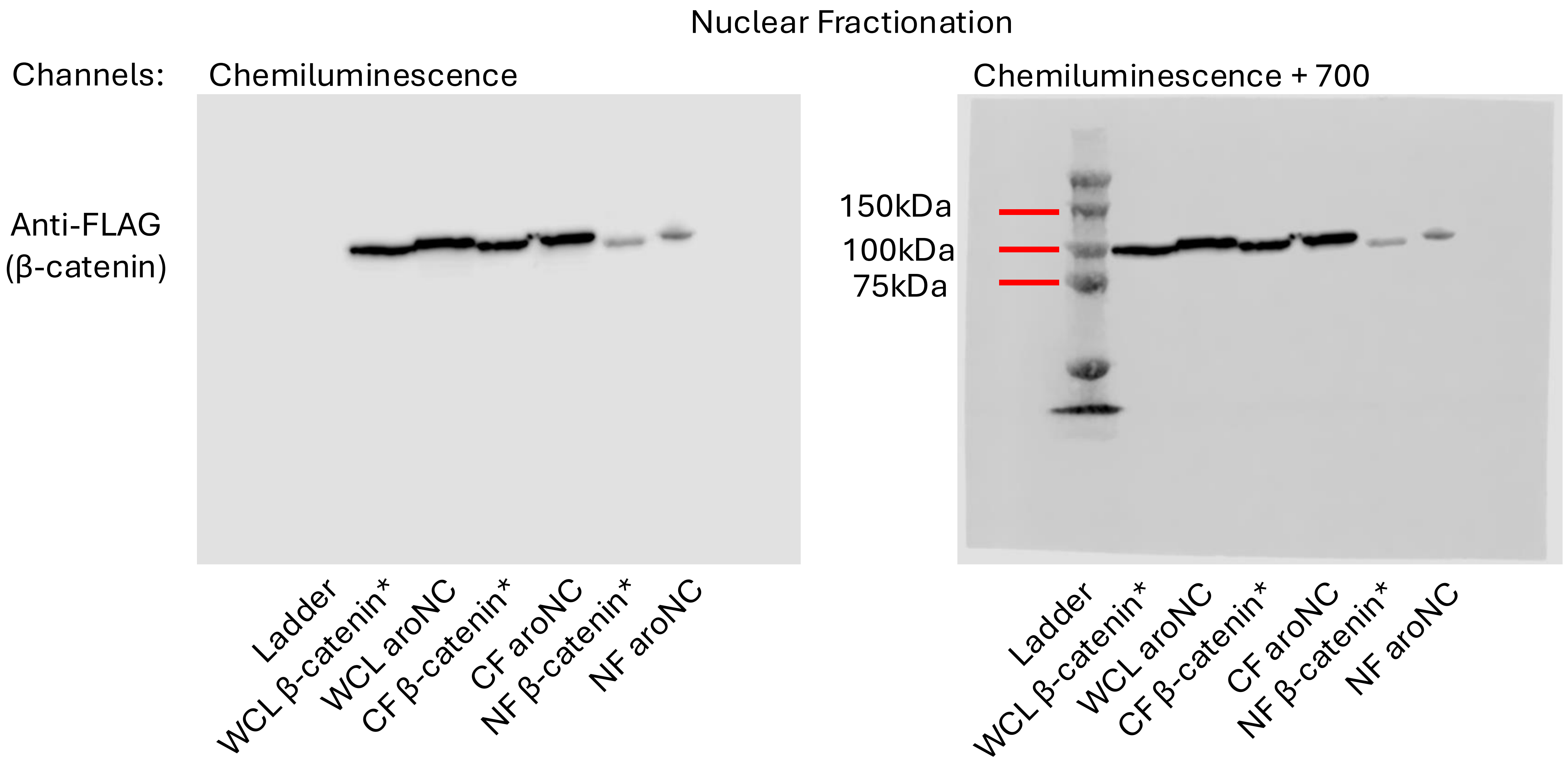
Nuclear Fractionation
Channels:
Chemiluminescence
Chemiluminescence + 700
Anti-FLAG
(β-catenin)
150kDa
100kDa
75kDa
Ladder
WCL β-catenin*
WCL aroNC
CF β-catenin*
CF aroNC
NF β-catenin*
NF aroNC
Ladder
WCL β-catenin*
WCL aroNC
CF β-catenin*
CF aroNC
NF β-catenin*
NF aroNC

Figure 4E

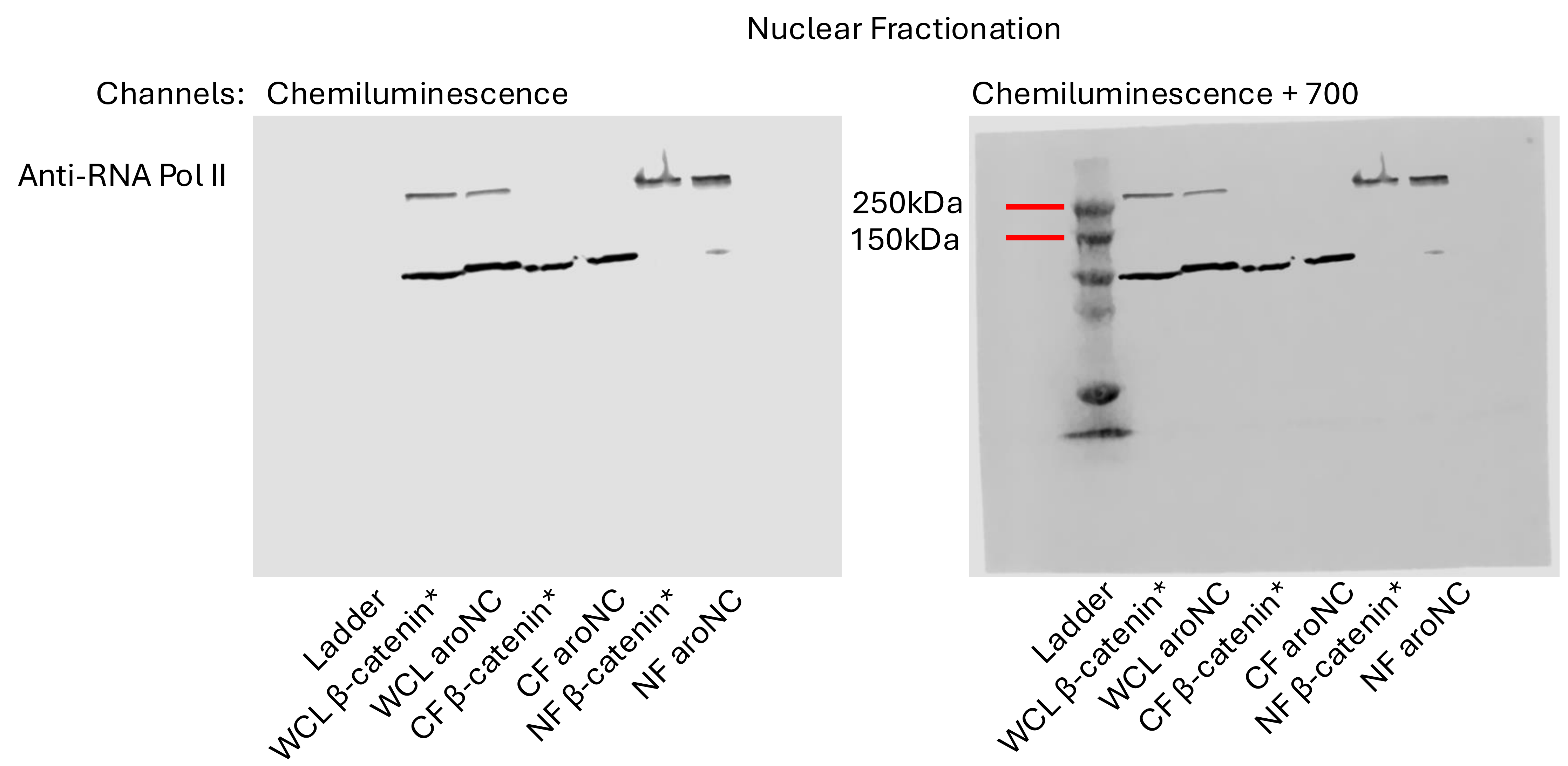
Nuclear Fractionation
Channels:
Chemiluminescence
Chemiluminescence + 700
Anti-RNA Pol II
250kDa
150kDa
Ladder
WCL β-catenin*
WCL aroNC
CF β-catenin*
CF aroNC
NF β-catenin*
NF aroNC
Ladder
WCL β-catenin*
WCL aroNC
CF β-catenin*
CF aroNC
NF β-catenin*
NF aroNC

Figure 4E

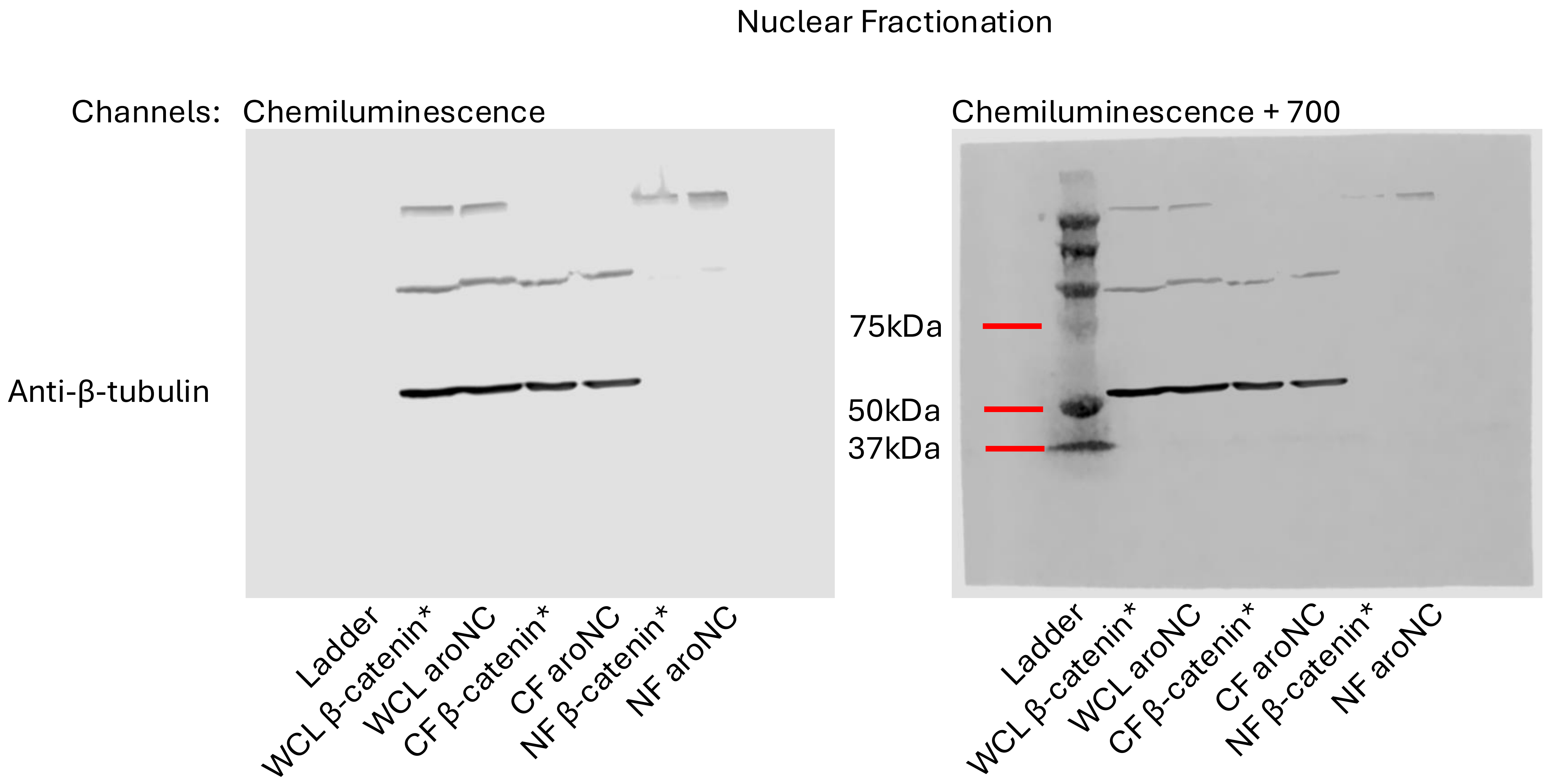
Nuclear Fractionation
Channels: Chemiluminescence
Chemiluminescence + 700
Anti-β-tubulin
75kDa
50kDa
37kDa
Ladder
WCL β-catenin*
WCL aroNC
CF β-catenin*
CF aroNC
NF β-catenin*
NF aroNC
Ladder
WCL β-catenin*
WCL aroNC
CF β-catenin*
CF aroNC
NF β-catenin*
NF aroNC

Figure 5B

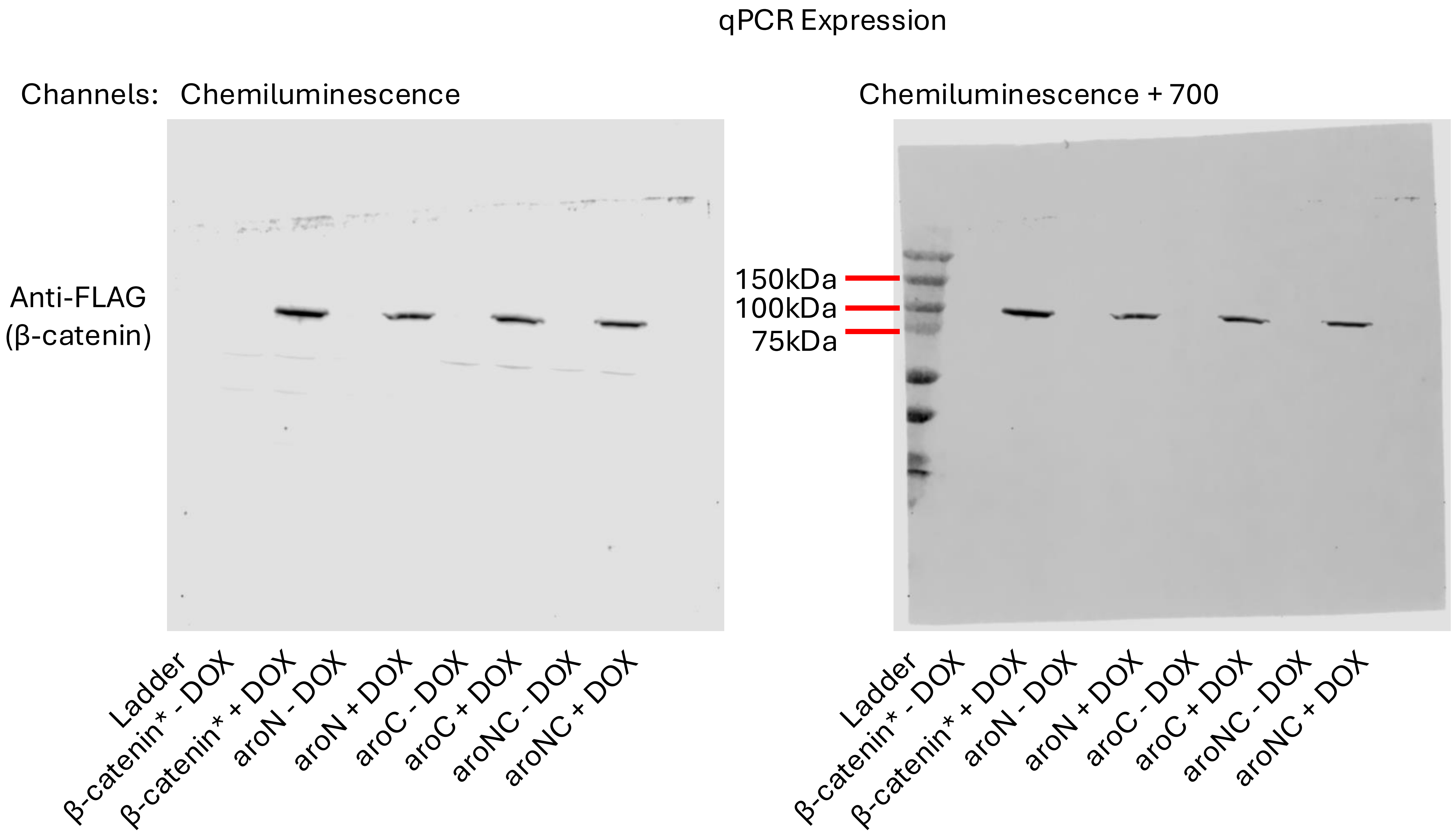
qPCR Expression
Channels: Chemiluminescence
Chemiluminescence + 700
Anti-FLAG
(β-catenin)
150kDa
100kDa
75kDa
Ladder
β-catenin* - DOX
β-catenin* + DOX
aroN - DOX
aroN + DOX
aroC - DOX
aroC + DOX
aroNC - DOX
aroNC + DOX
Ladder
β-catenin* - DOX
β-catenin* + DOX
aroN - DOX
aroN + DOX
aroC - DOX
aroC + DOX
aroNC - DOX
aroNC + DOX

Figure 5B

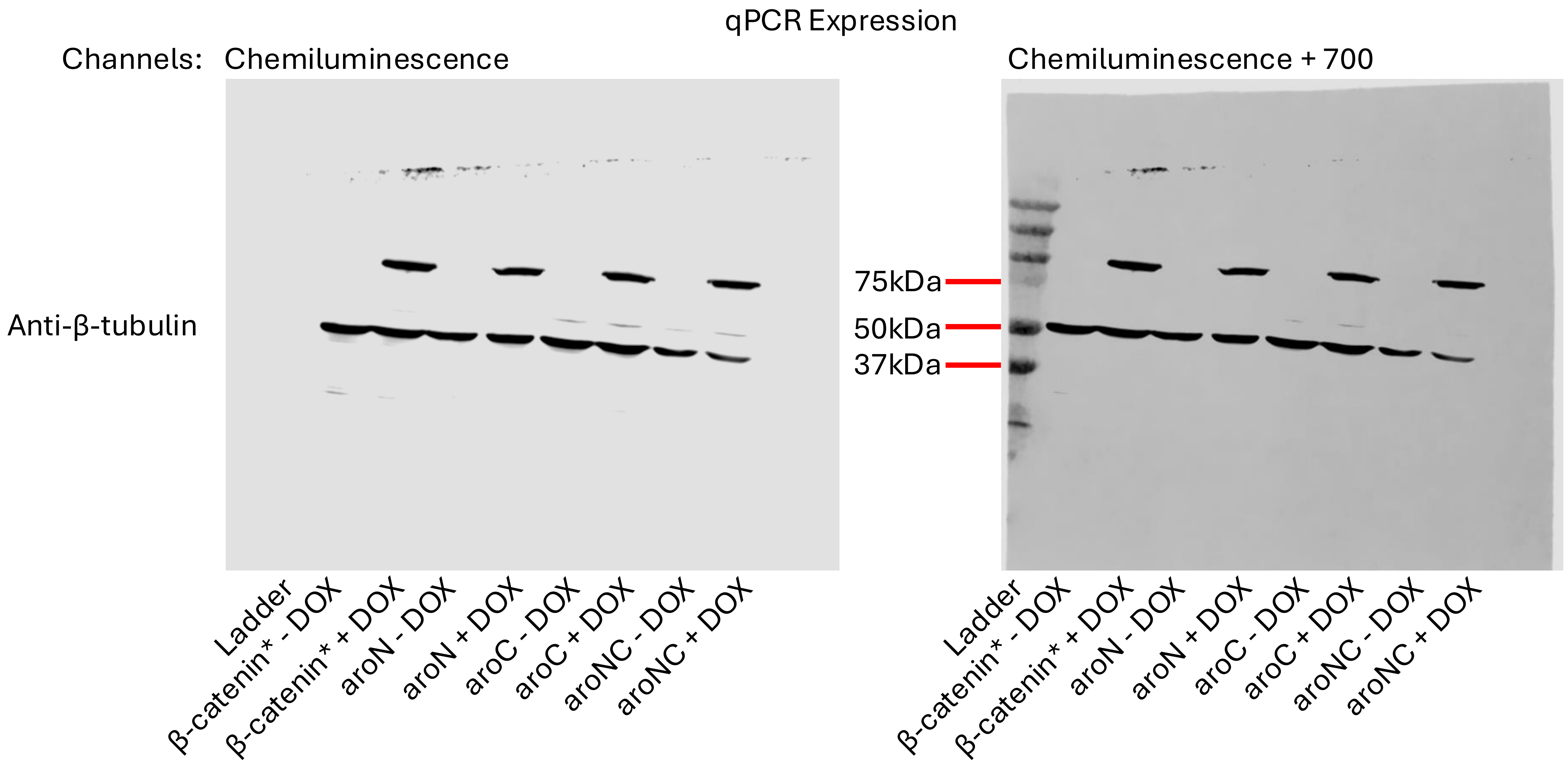
qPCR Expression
Channels:
Chemiluminescence
Chemiluminescence + 700
Anti-β-tubulin
75kDa
50kDa
37kDa
Ladder
β-catenin* - DOX
β-catenin* + DOX
aroN - DOX
aroN + DOX
aroC - DOX
aroC + DOX
aroNC - DOX
aroNC + DOX
Ladder
β-catenin* - DOX
β-catenin* + DOX
aroN - DOX
aroN + DOX
aroC - DOX
aroC + DOX
aroNC - DOX
aroNC + DOX

Figure 10A

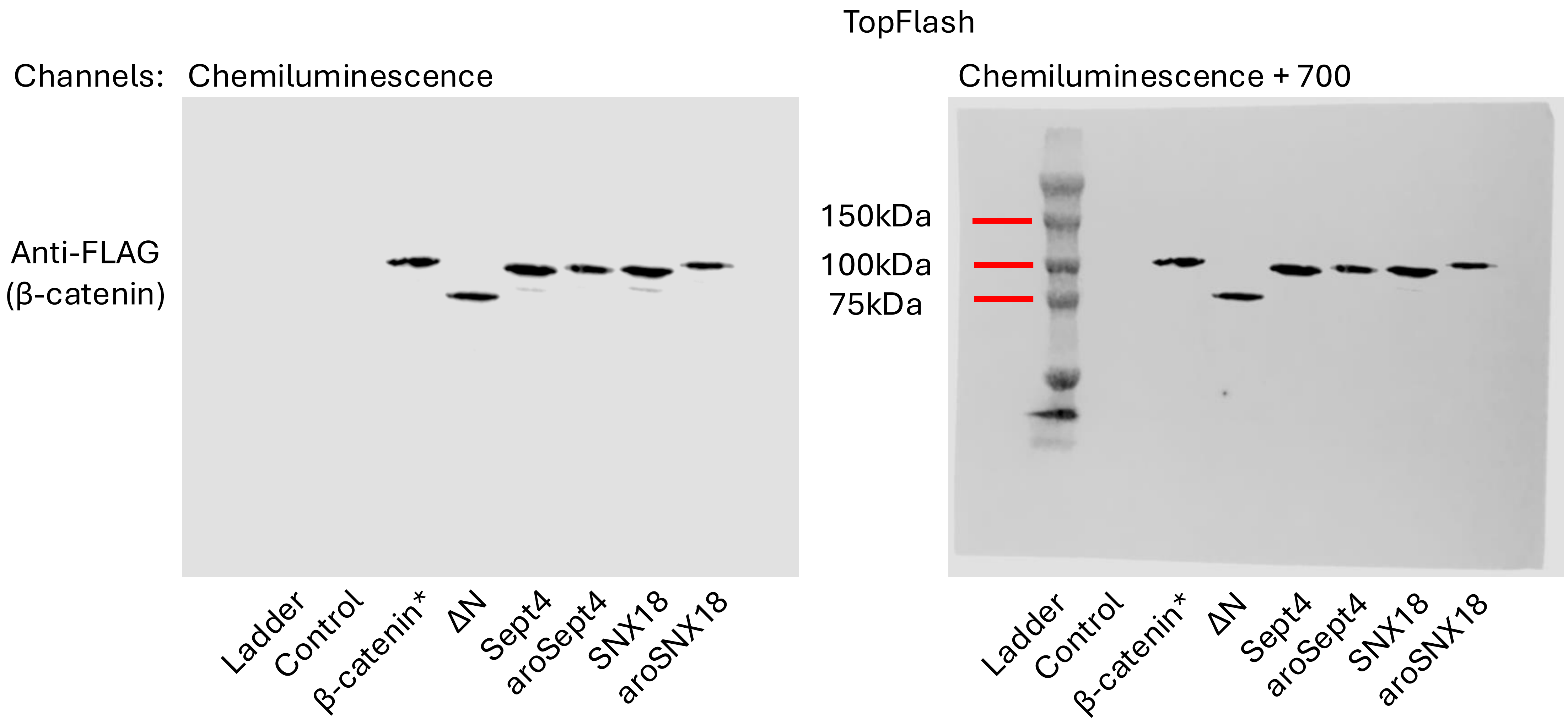
TopFlash
Channels: Chemiluminescence
Chemiluminescence + 700
Anti-FLAG
(β-catenin)
150kDa
100kDa
75kDa
Ladder
Control
β-catenin*
ΔN
Sept4
aroSept4
SNX18
aroSNX18
Ladder
Control
β-catenin*
ΔN
Sept4
aroSept4
SNX18
aroSNX18

Figure 10A

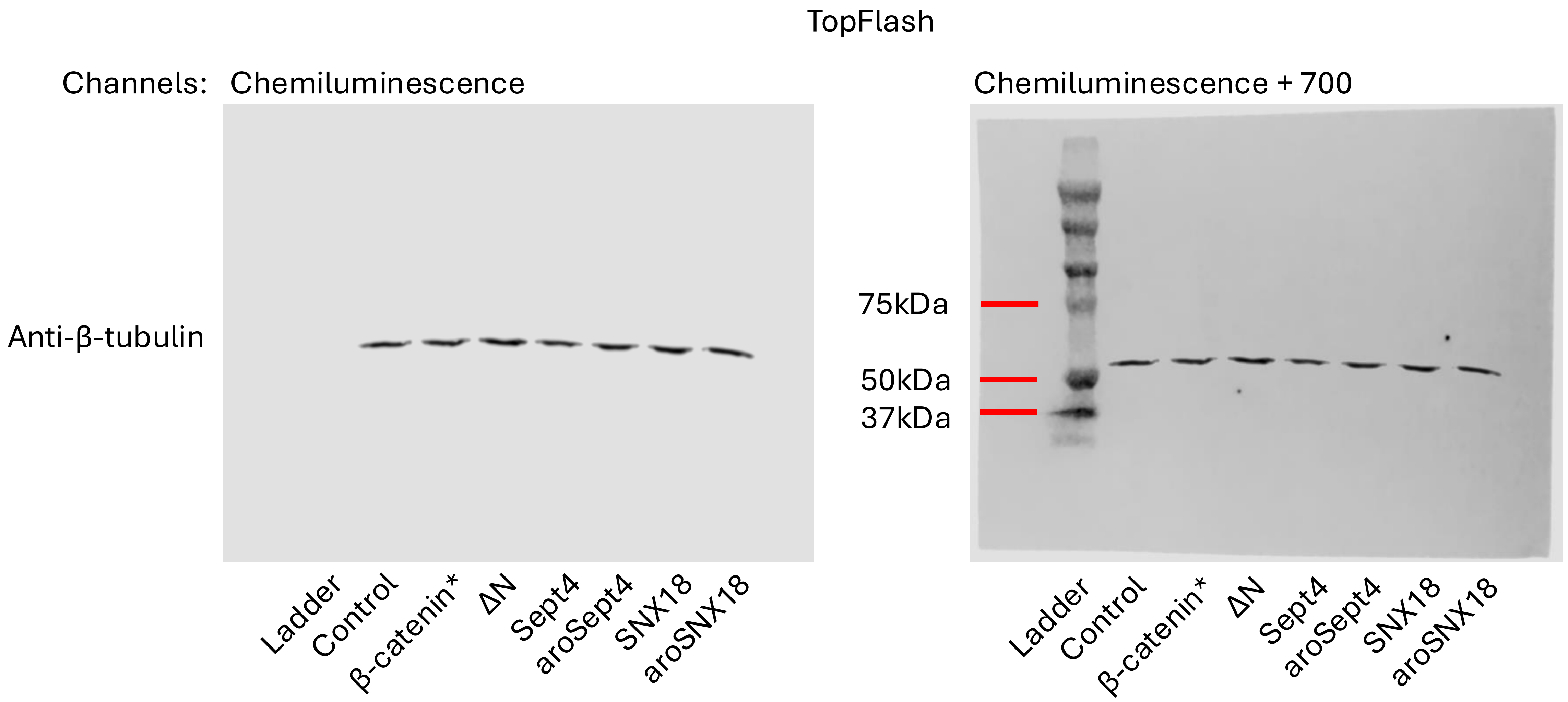
TopFlash
Channels:
Chemiluminescence
Chemiluminescence + 700
Anti-β-tubulin
75kDa
50kDa
37kDa
Ladder
Control
β-catenin*
ΔN
Sept4
aroSept4
SNX18
aroSNX18
Ladder
Control
β-catenin*
ΔN
Sept4
aroSept4
SNX18
aroSNX18

S3B Fig

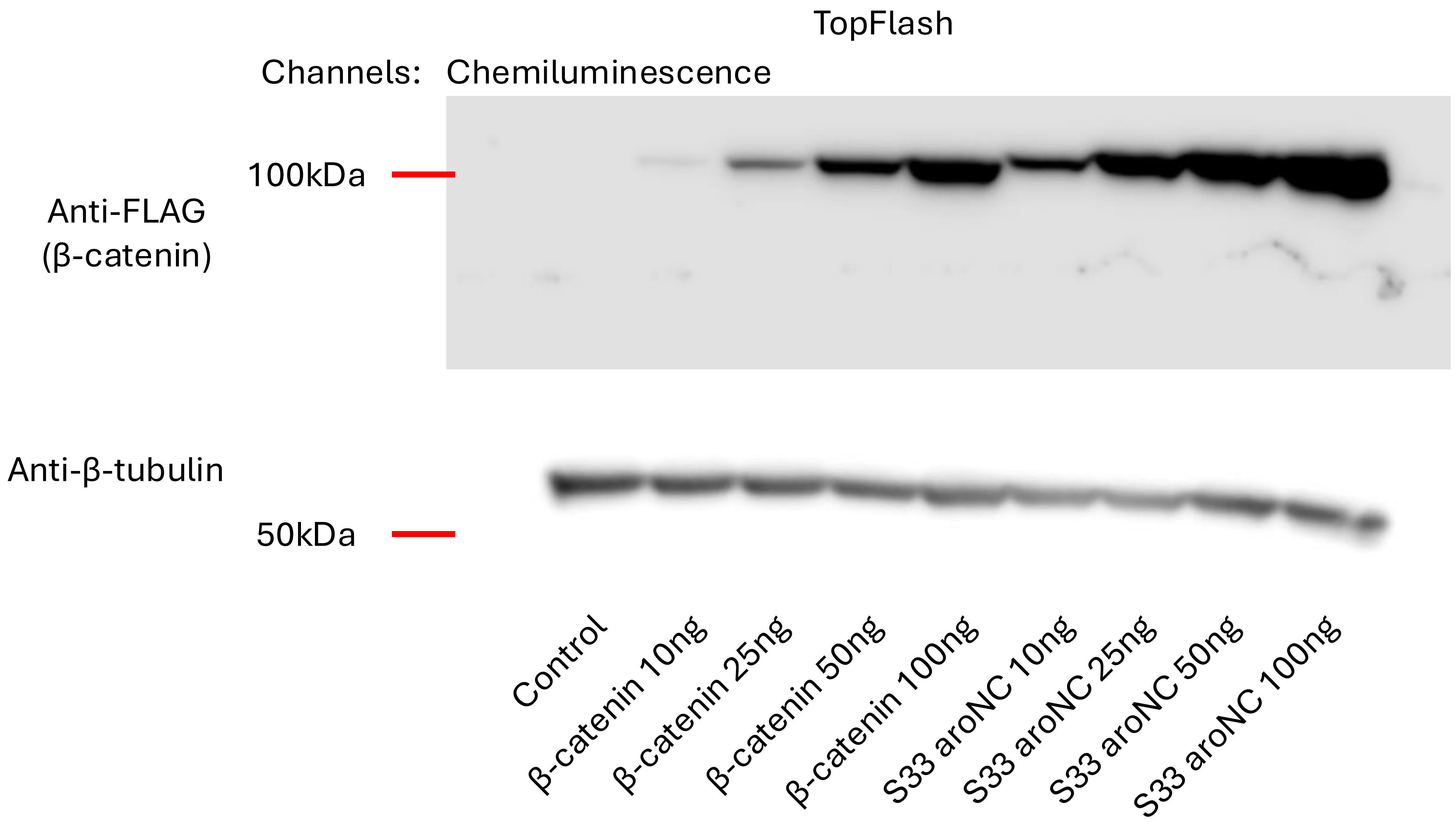
TopFlash
Channels: Chemiluminescence
100kDa
Anti-FLAG
(β-catenin)
Anti-β-tubulin
50kDa
Control
β-catenin 10ng
β-catenin 25ng
β-catenin 50ng
β-catenin 100ng
S33 aroNC 10ng
S33 aroNC 25ng
S33 aroNC 50ng
S33 aroNC 100ng

S10A Fig

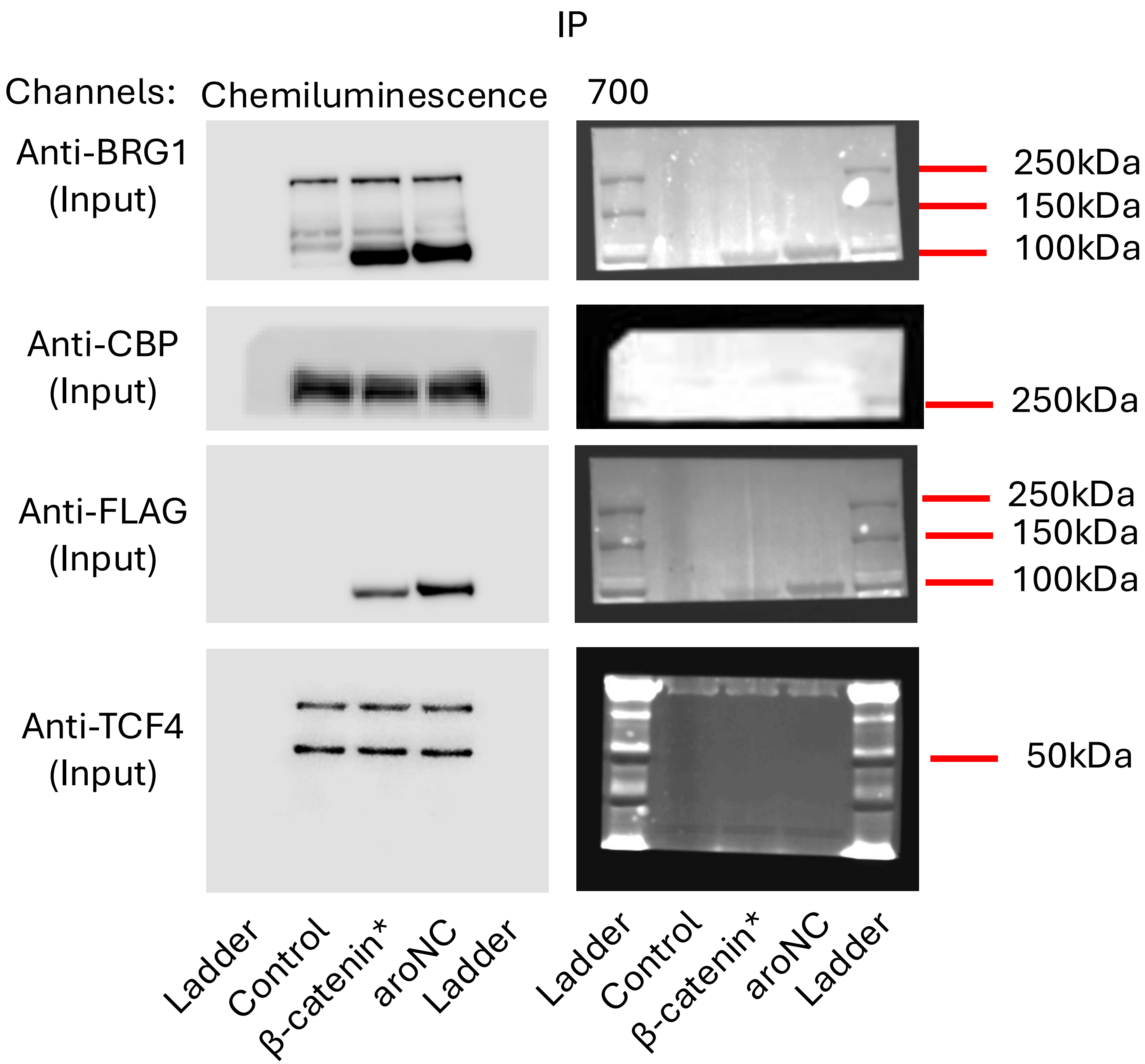
IP
Channels:
Chemiluminescence
700
Anti-BRG1 (Input)
Anti-CBP (Input)
Anti-FLAG (Input)
Anti-TCF4 (Input)
250kDa
150kDa
100kDa
250kDa
250kDa
150kDa
100kDa
50kDa
Ladder
Control
β-catenin*
aroNC
Ladder
Ladder
Control
β-catenin*
aroNC
Ladder

S10A Fig

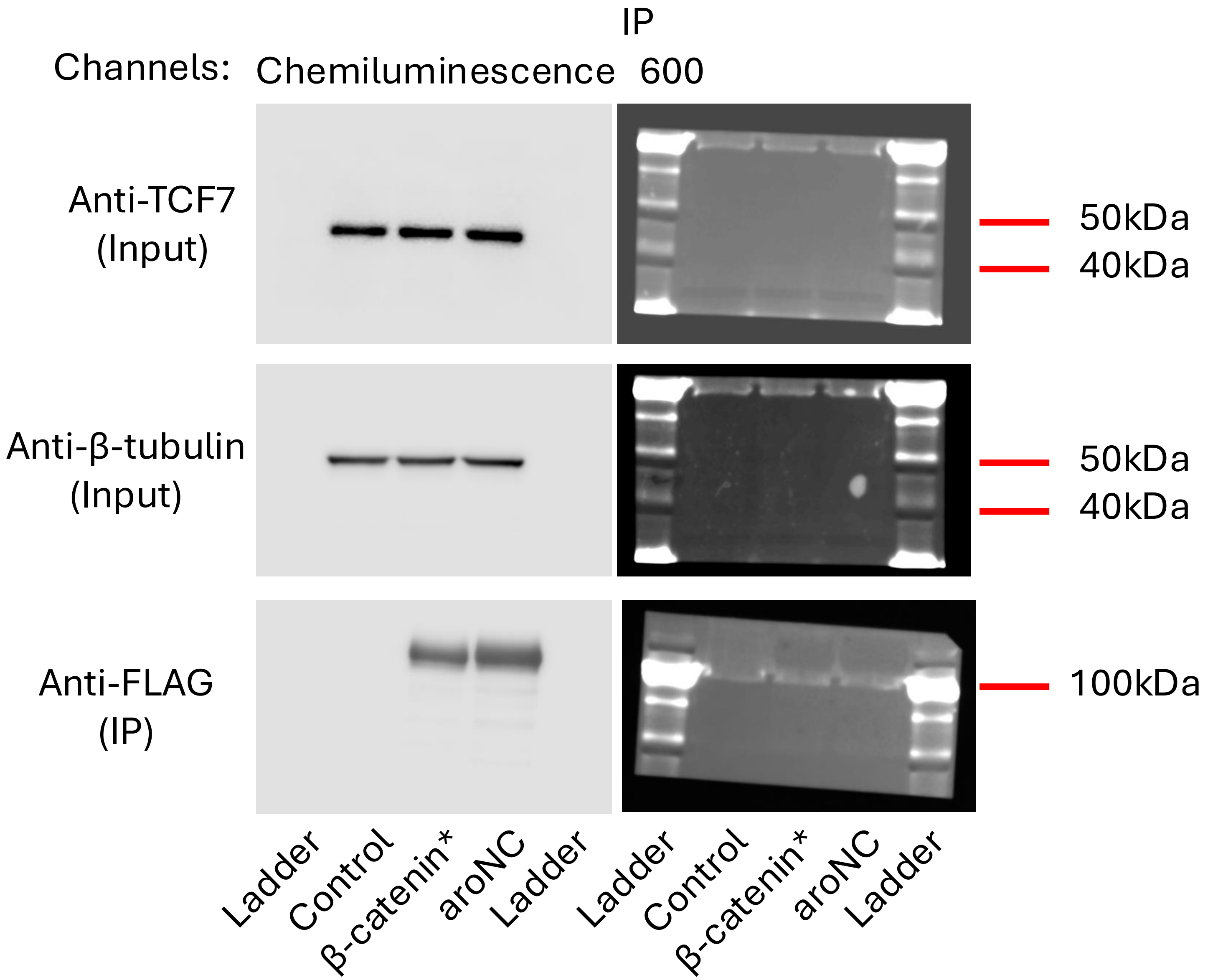
IP
Channels:
Chemiluminescence
600
Anti-TCF7
(Input)
Anti-β-tubulin
(Input)
Anti-FLAG
(IP)
50kDa
40kDa
50kDa
40kDa
100kDa
Ladder
Control
β-catenin*
aroNC
Ladder
Ladder
Control
β-catenin*
aroNC
Ladder

S10A Fig

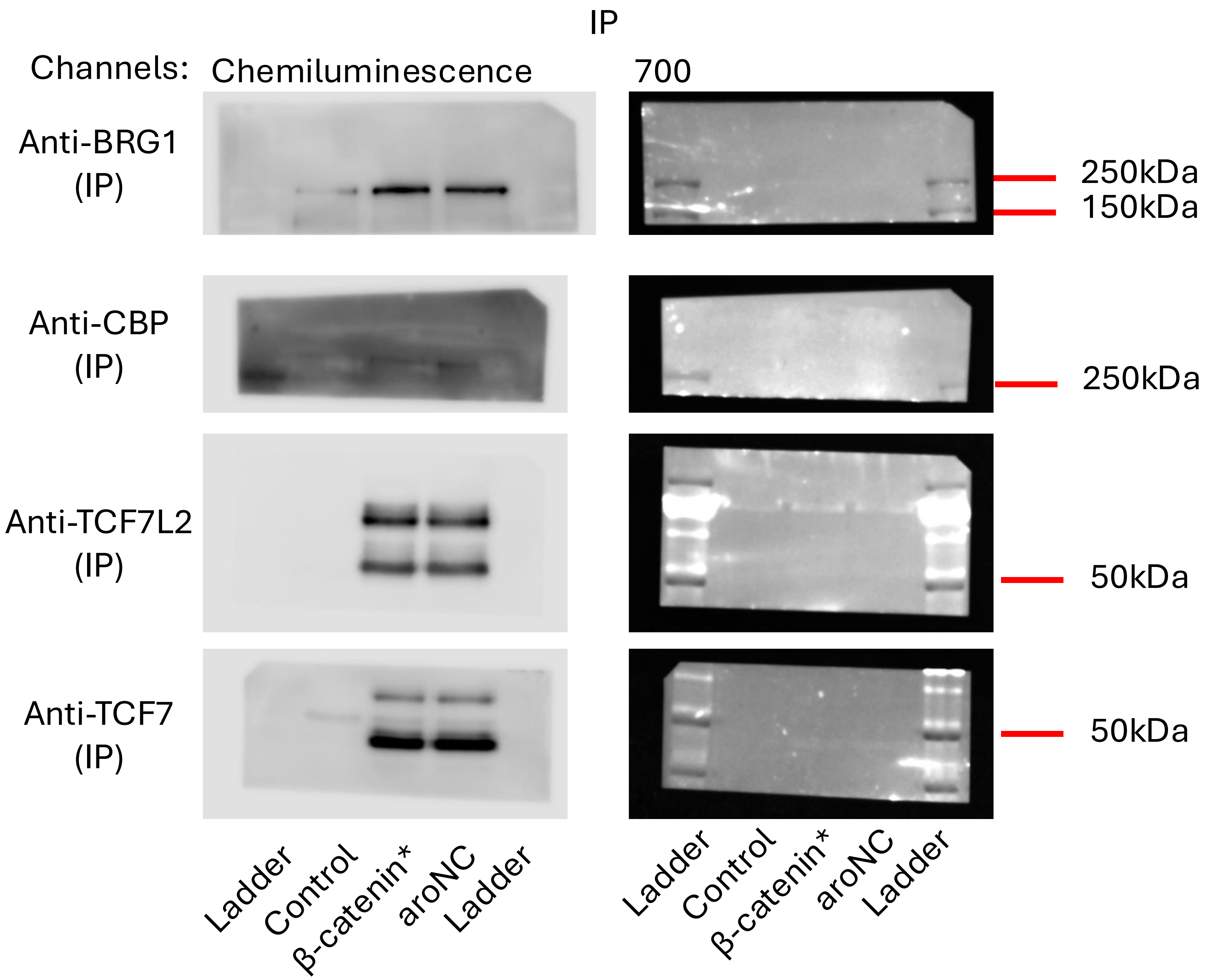
IP
Channels:
Chemiluminescence
700
Anti-BRG1 (IP)
Anti-CBP (IP)
Anti-TCF7L2 (IP)
Anti-TCF7 (IP)
250kDa
150kDa
250kDa
50kDa
50kDa
Ladder
Control
β-catenin*
aroNC
Ladder
Ladder
Control
β-catenin*
aroNC
Ladder

S10B Fig

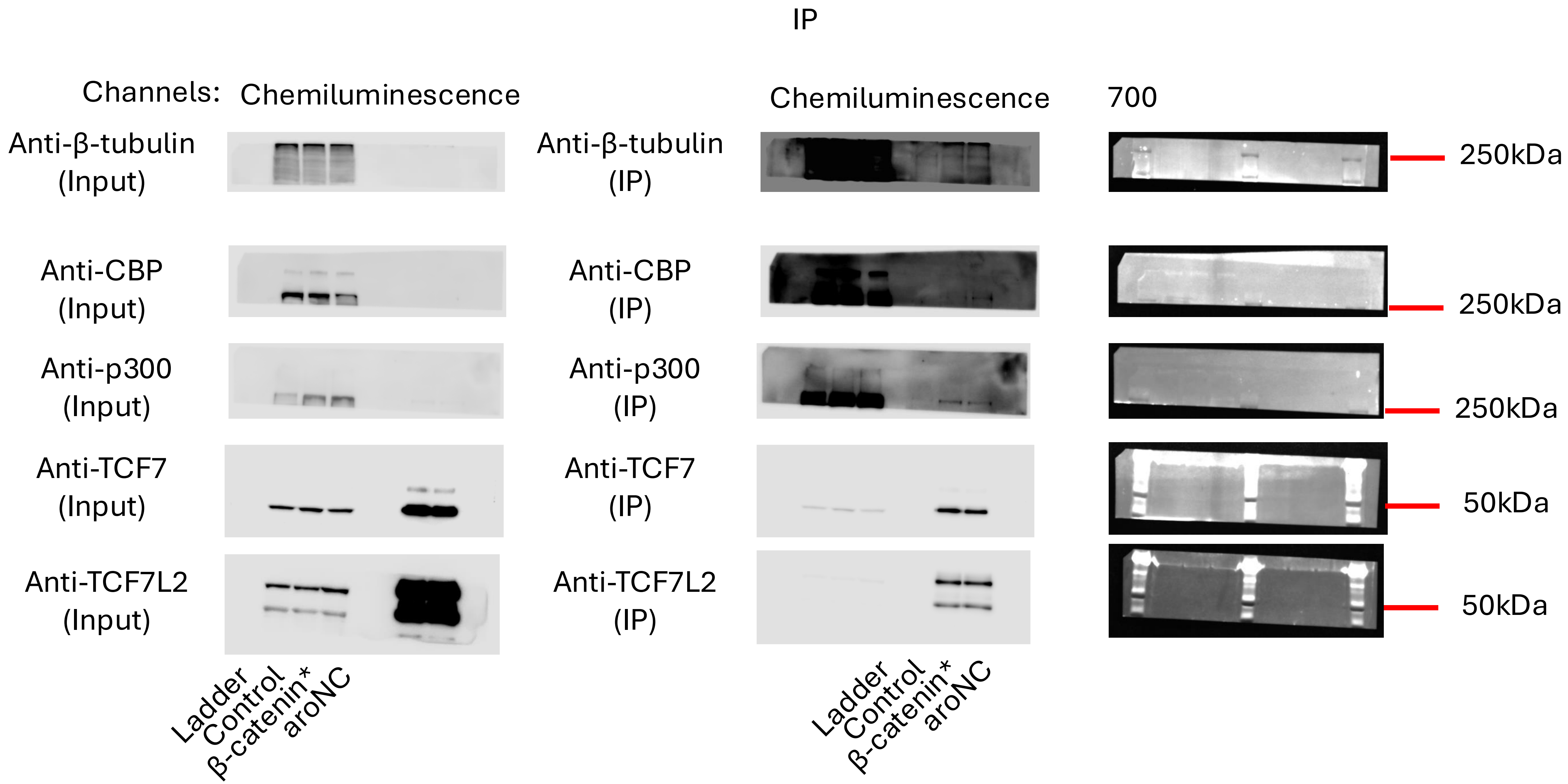
IP
Channels:
Chemiluminescence
Chemiluminescence
700
Anti-β-tubulin (Input)
Anti-β-tubulin (IP)
250kDa
Anti-CBP (Input)
Anti-CBP (IP)
250kDa
Anti-p300 (Input)
Anti-p300 (IP)
250kDa
Anti-TCF7 (Input)
Anti-TCF7 (IP)
50kDa
Anti-TCF7L2 (Input)
Anti-TCF7L2 (IP)
50kDa
Ladder
Control
β-catenin*
aroNC
Ladder
Control
β-catenin*
aroNC

S10B Fig

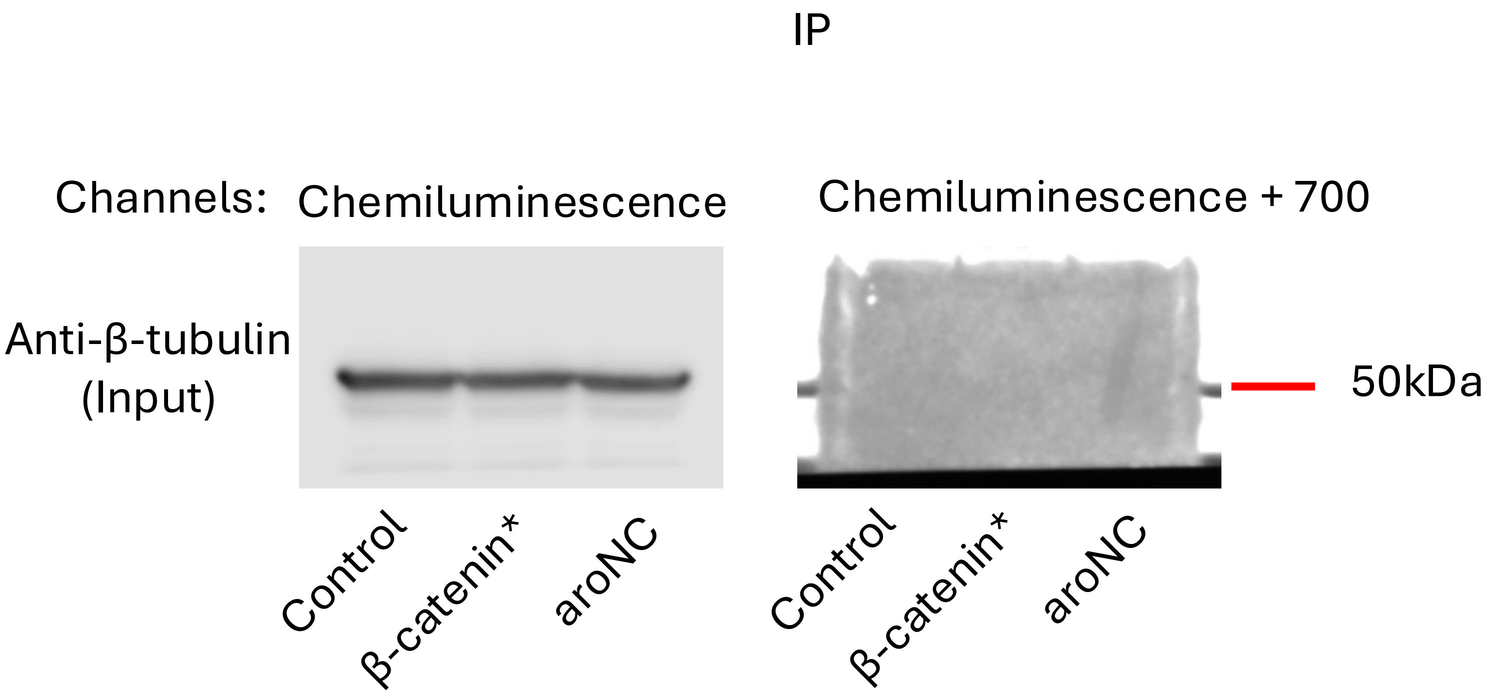
IP
Channels:
Chemiluminescence
Chemiluminescence + 700
Anti-β-tubulin
(Input)
50kDa
Control
β-catenin*
aroNC
Control
β-catenin*
aroNC

S11B Fig

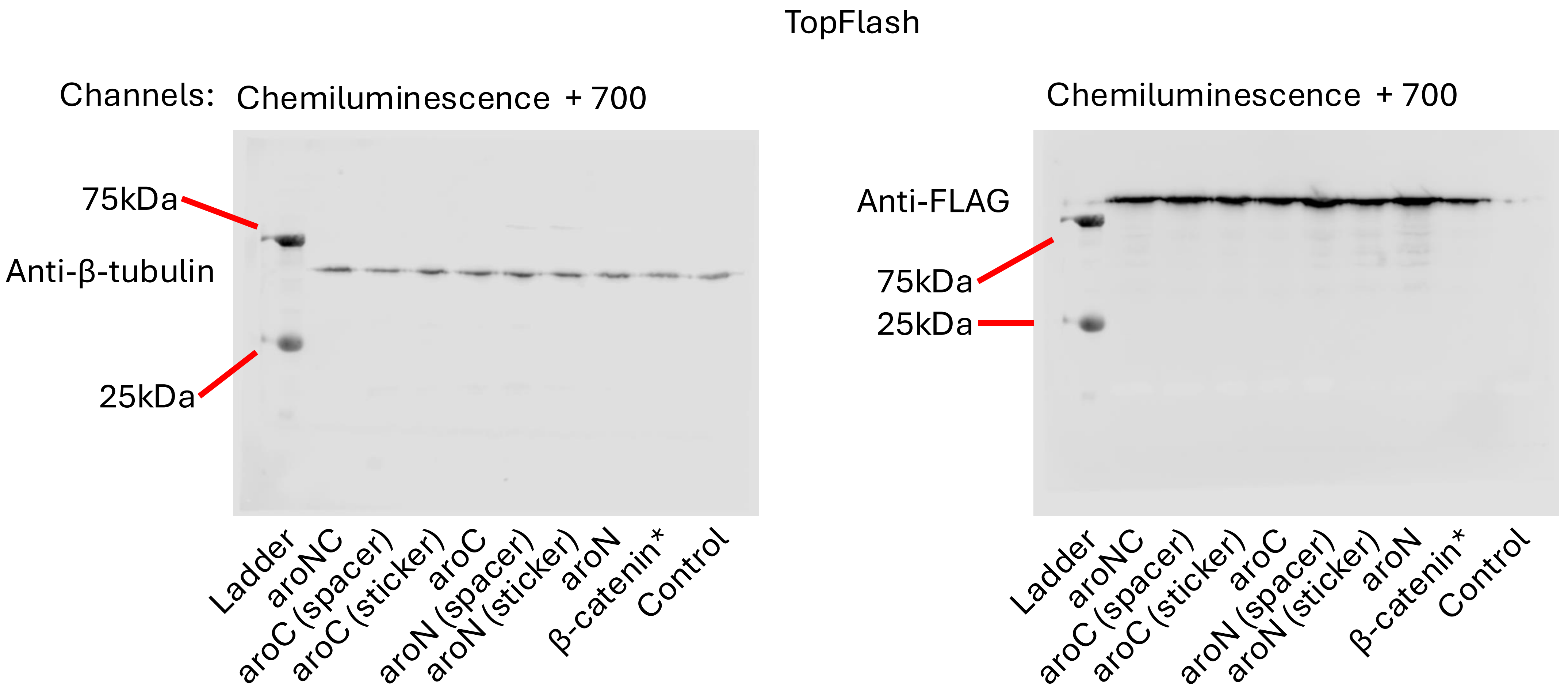
TopFlash
Channels:
Chemiluminescence + 700
75kDa
Anti-β-tubulin
25kDa
Ladder
aroNC
aroC (spacer)
aroC (sticker)
aroC
aroN (spacer)
aroN (sticker)
aroN
β-catenin*
Control
Chemiluminescence + 700
Anti-FLAG
75kDa
25kDa
Ladder
aroNC
aroC (spacer)
aroC (sticker)
aroC
aroN (spacer)
aroN (sticker)
aroN
β-catenin*
Control

S11B Fig

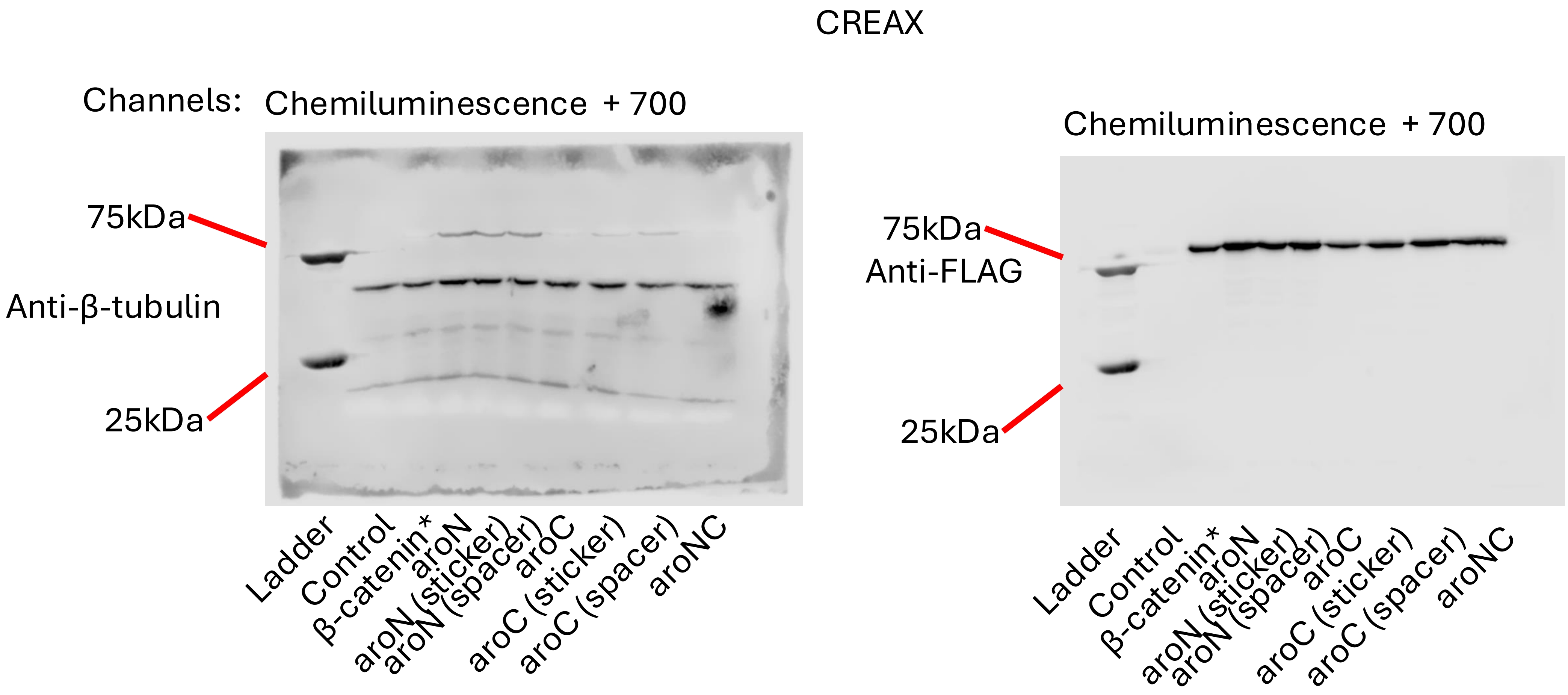
CREAX
Channels:
Chemiluminescence + 700
75kDa
Anti-β-tubulin
25kDa
Ladder
Control
β-catenin*
aroN
aroN (sticker)
aroN (spacer)
aroC
aroC (sticker)
aroC (spacer)
aroNC
Chemiluminescence + 700
75kDa
Anti-FLAG
25kDa
Ladder
Control
β-catenin*
aroN
aroN (sticker)
aroN (spacer)
aroC
aroC (sticker)
aroC (spacer)
aroNC

S11C Fig

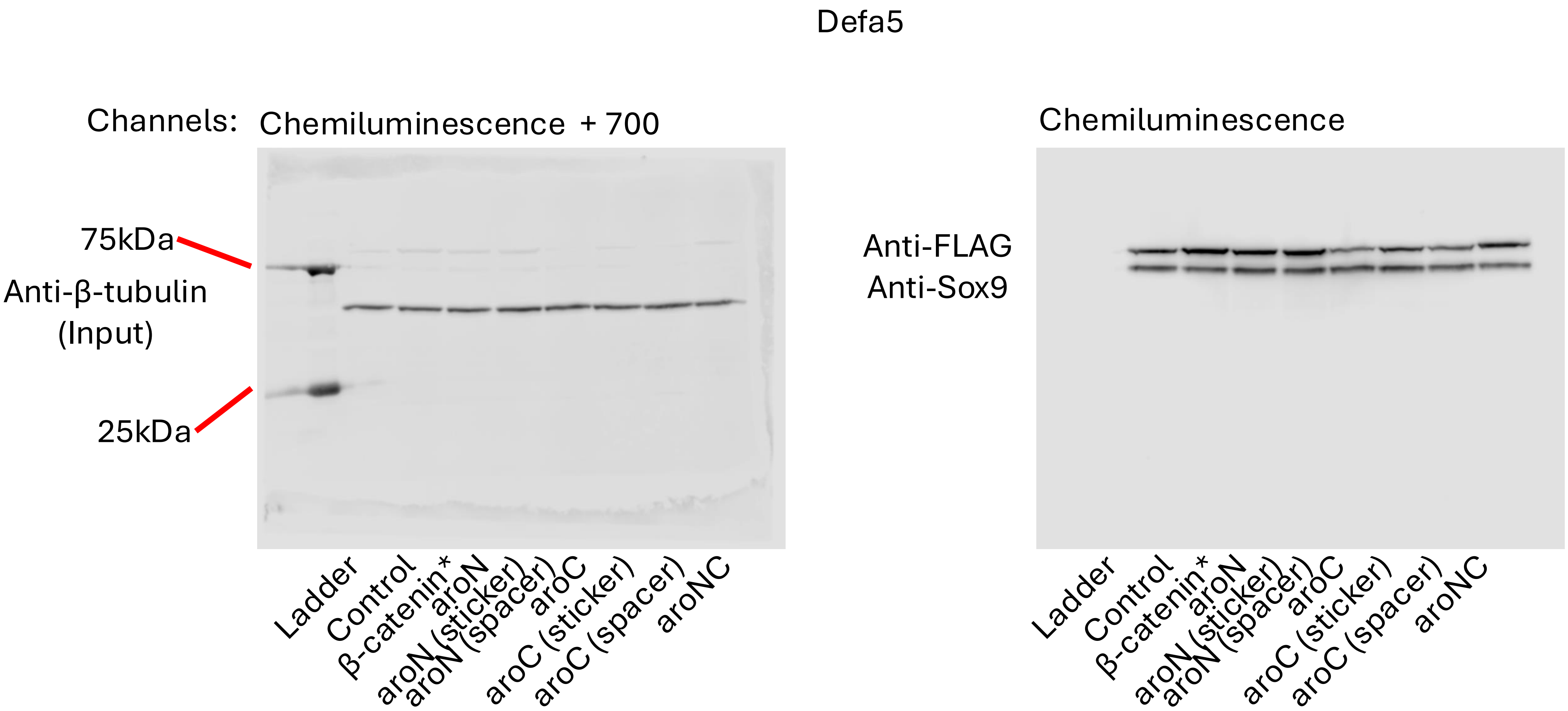
Defa5
Channels:
Chemiluminescence + 700
Chemiluminescence
75kDa
Anti-β-tubulin
(Input)
25kDa
Anti-FLAG
Anti-Sox9
Ladder
Control
β-catenin*
aroN
aroN (sticker)
aroN (spacer)
aroC
aroC (sticker)
aroC (spacer)
aroNC
Ladder
Control
β-catenin*
aroN
aroN (sticker)
aroN (spacer)
aroC
aroC (sticker)
aroC (spacer)
aroNC

S12B Fig

TopFlash

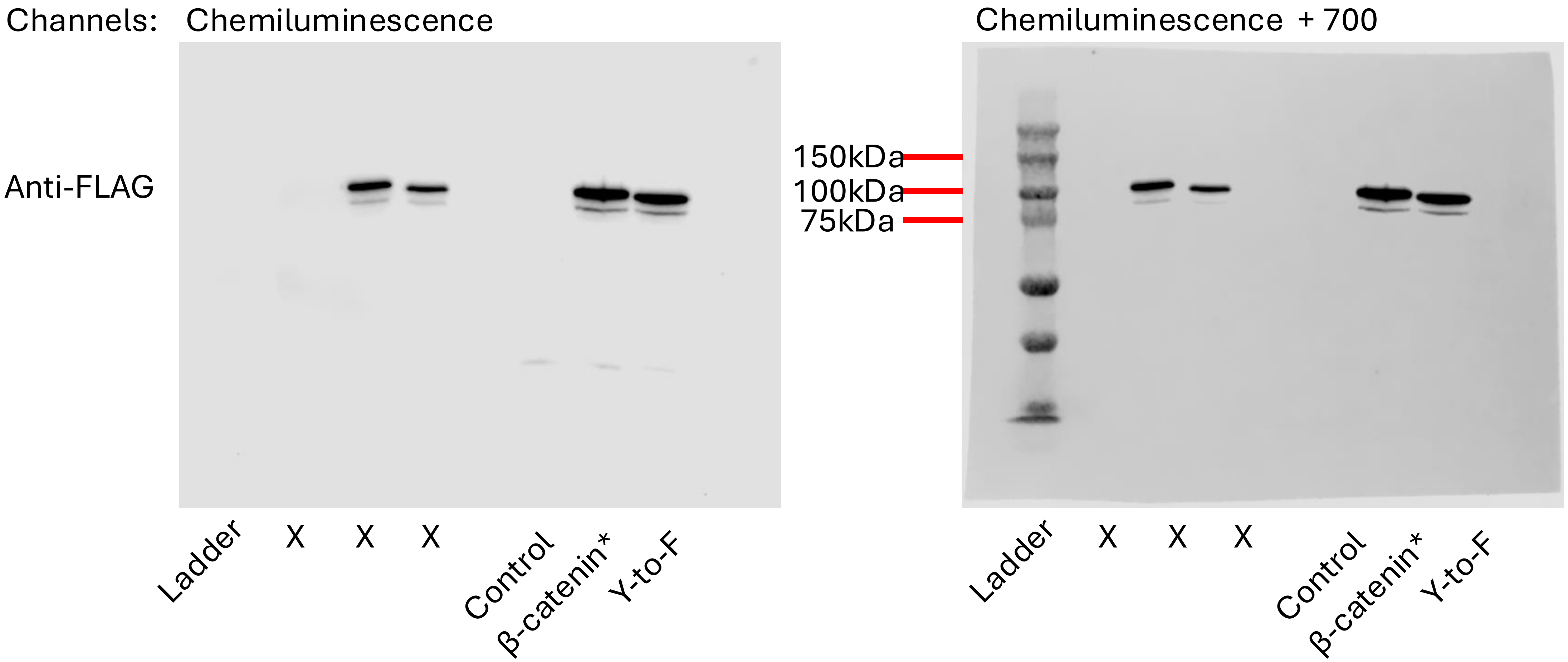

S12B Fig

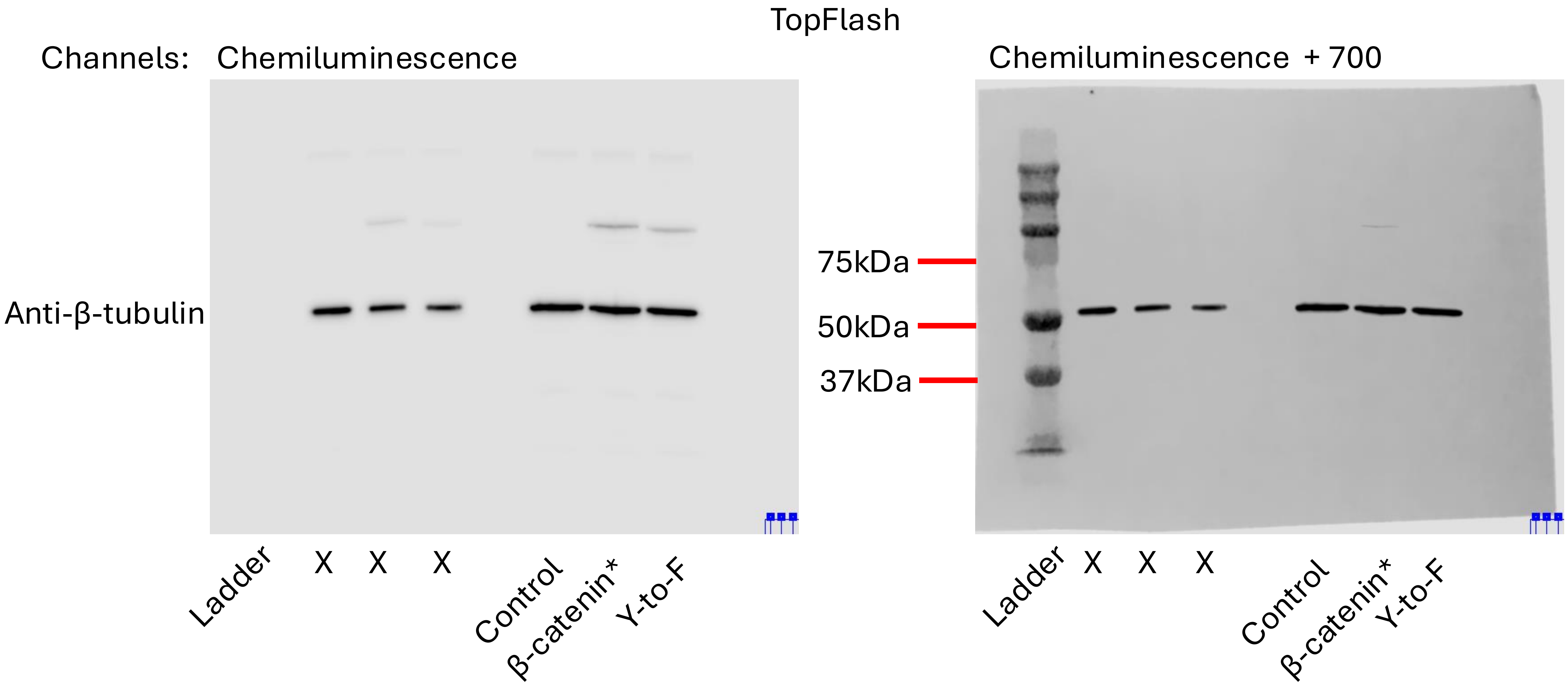
TopFlash
Channels: Chemiluminescence
Chemiluminescence + 700
Anti-β-tubulin
75kDa
50kDa
37kDa
Ladder
X
X
X
Control
β-catenin*
Y-to-F
Ladder
X
X
X
Control
β-catenin*
Y-to-F

S12C Fig

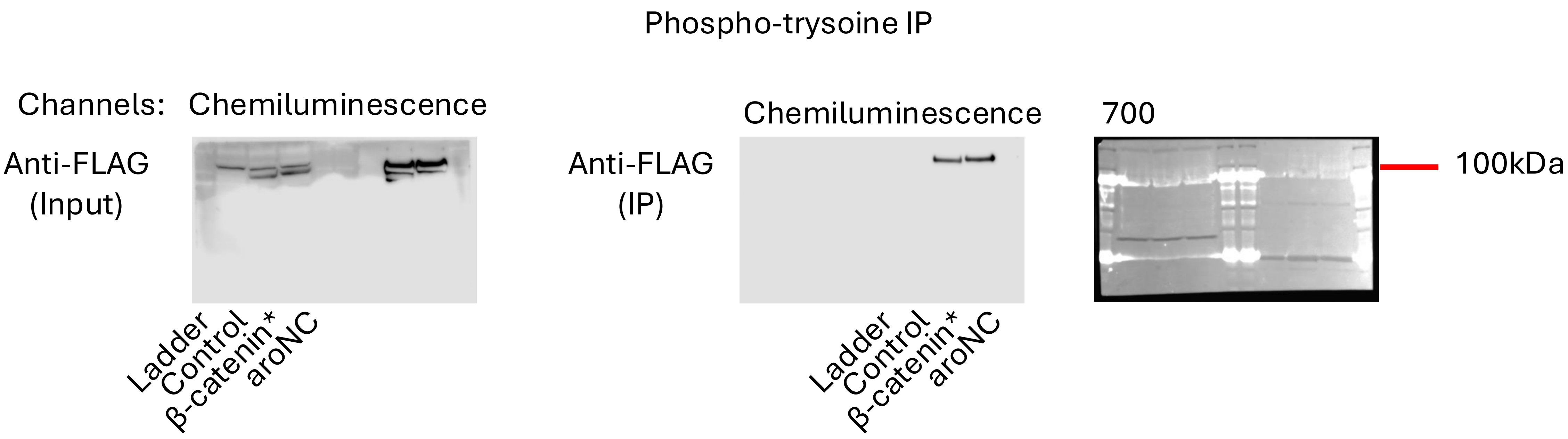
Phospho-trysoine IP
Channels:
Chemiluminescence
Anti-FLAG
(Input)
Ladder
Control
β-catenin*
aroNC
Chemiluminescence
Anti-FLAG
(IP)
Ladder
Control
β-catenin*
aroNC
700
100kDa

S12C Fig

Phospho-tryosine IP

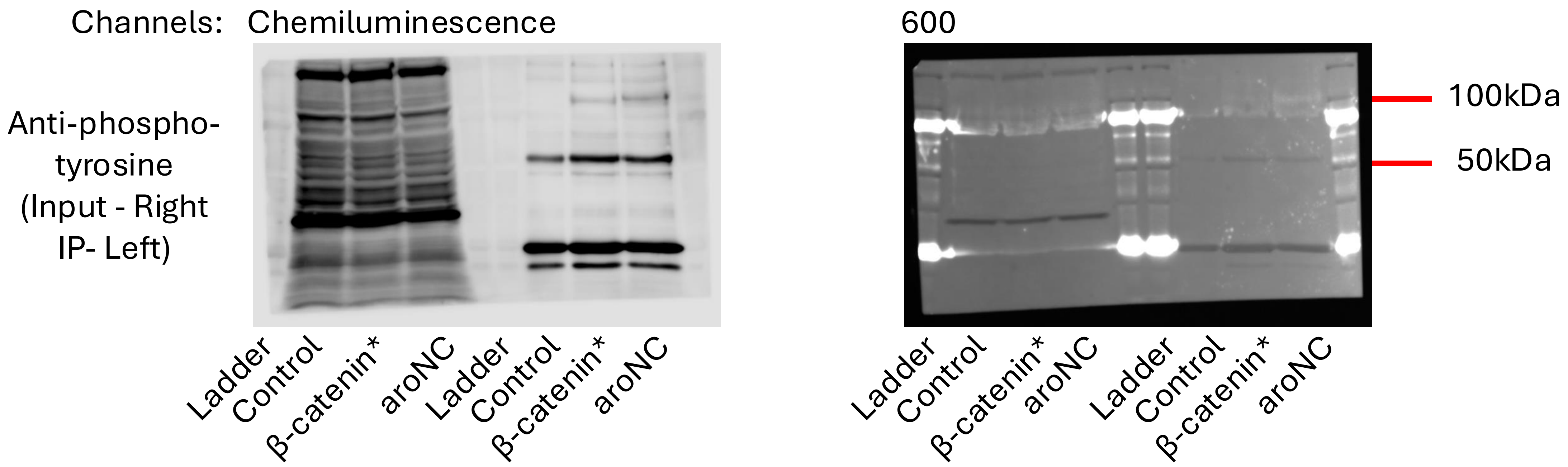

S13A Fig

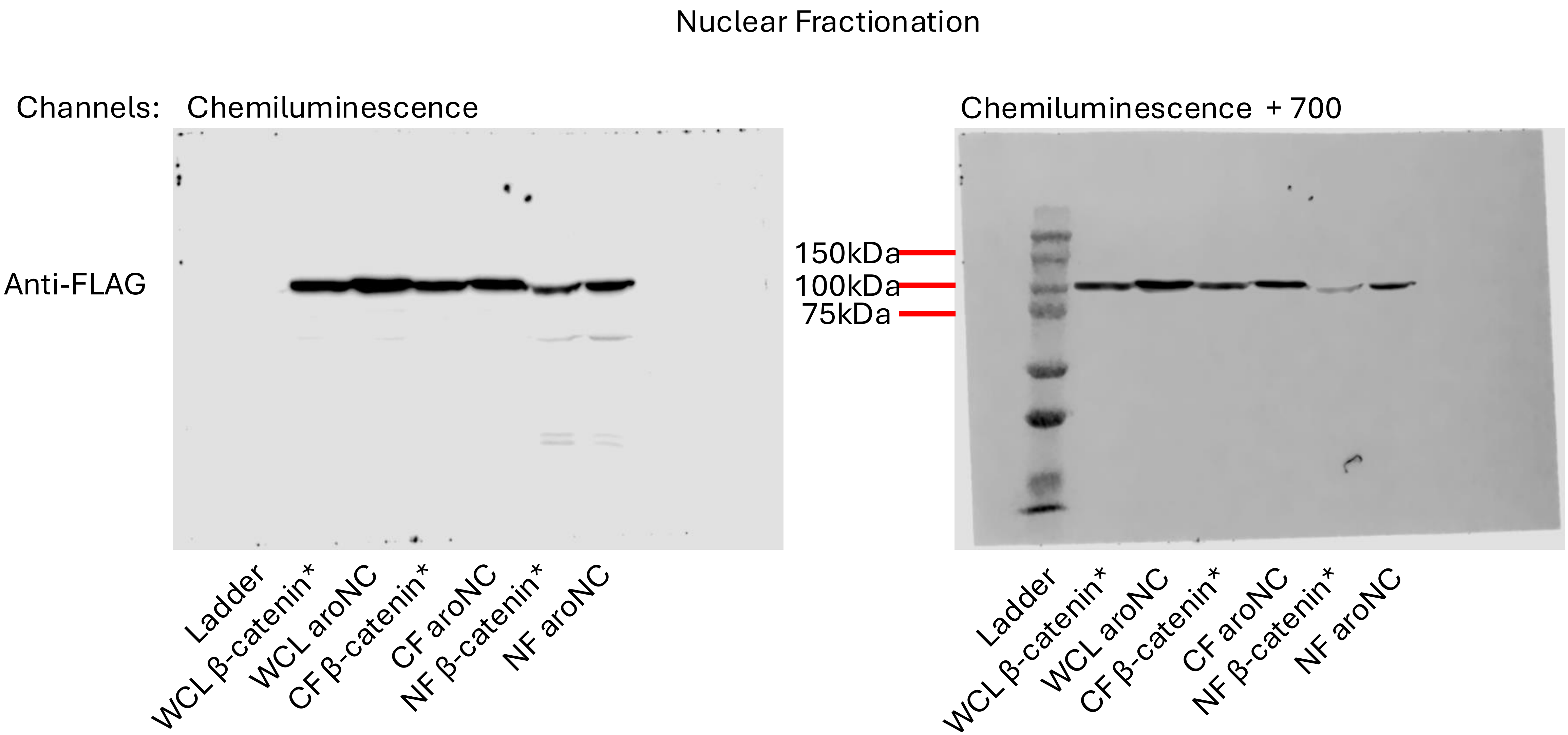
Nuclear Fractionation
Channels: Chemiluminescence
Chemiluminescence + 700
Anti-FLAG
150kDa
100kDa
75kDa
Ladder
WCL β-catenin*
WCL aroNC
CF β-catenin*
CF aroNC
NF β-catenin*
NF aroNC
Ladder
WCL β-catenin*
WCL aroNC
CF β-catenin*
CF aroNC
NF β-catenin*
NF aroNC

S13A Fig

Nuclear Fractionation

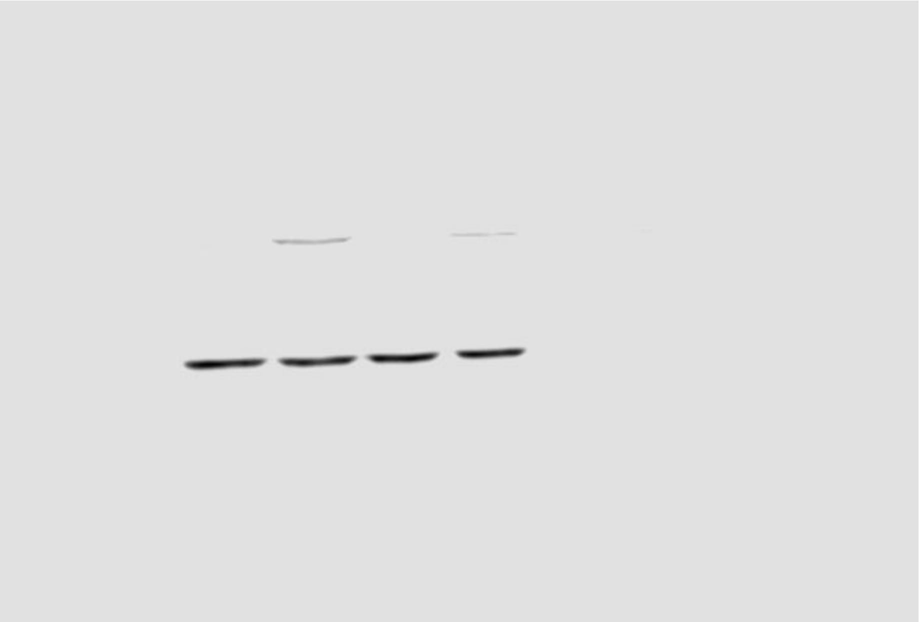

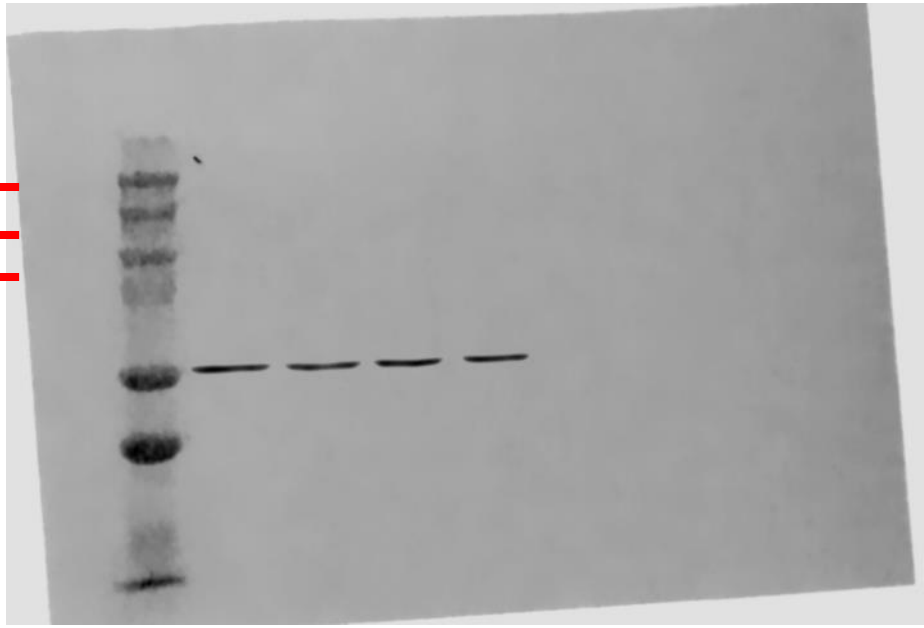

S13A Fig

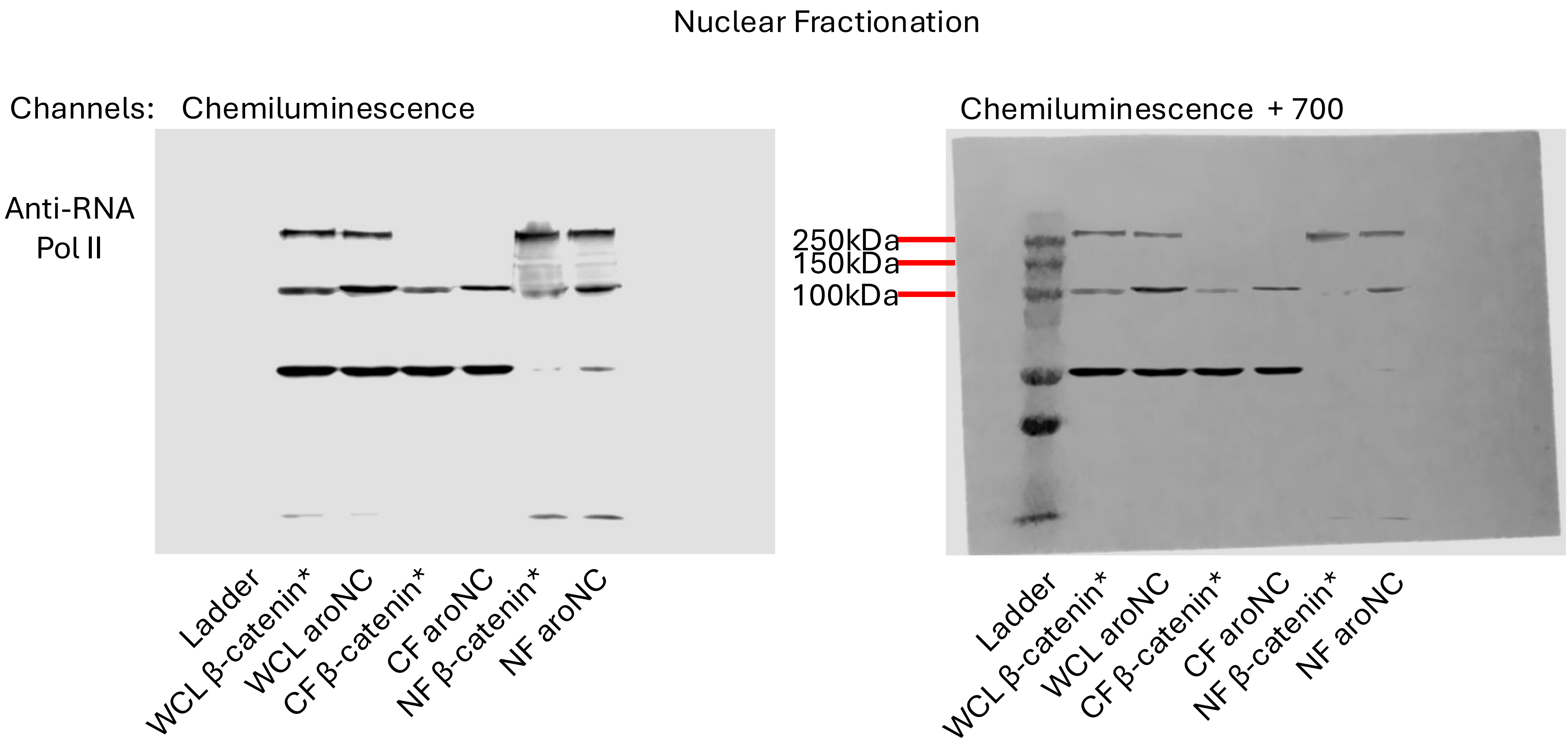
Nuclear Fractionation
Channels: Chemiluminescence
Chemiluminescence + 700
Anti-RNA Pol II
250kDa
150kDa
100kDa
Ladder
WCL β-catenin*
WCL aroNC
CF β-catenin*
CF aroNC
NF β-catenin*
NF aroNC
Ladder
WCL β-catenin*
WCL aroNC
CF β-catenin*
CF aroNC
NF β-catenin*
NF aroNC

S13C Fig

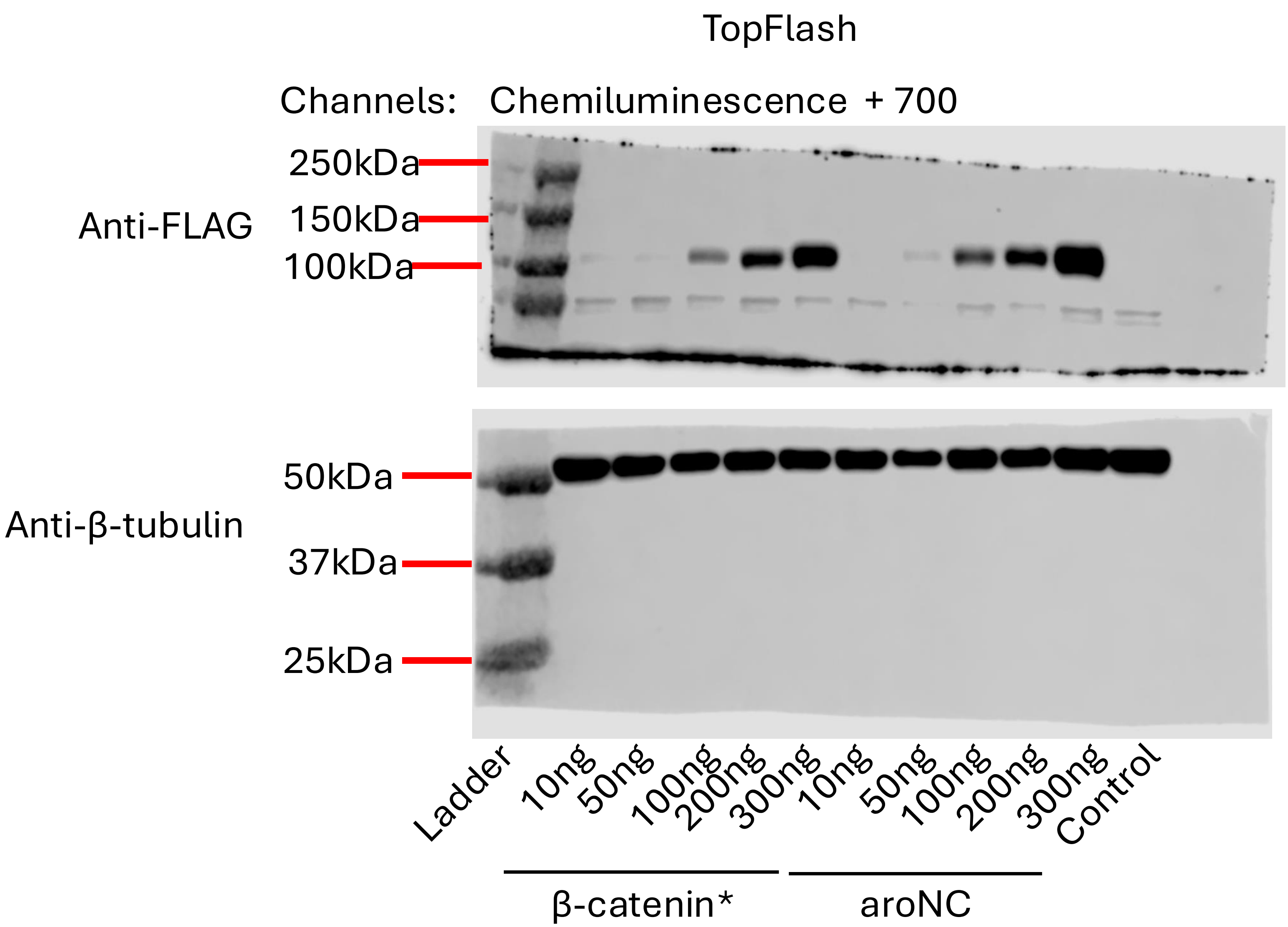
TopFlash
Channels: Chemiluminescence + 700
Anti-FLAG
250kDa
150kDa
100kDa
Anti-β-tubulin
50kDa
37kDa
25kDa
Ladder
10ng
50ng
100ng
200ng
300ng
10ng
50ng
100ng
200ng
300ng
Control
β-catenin*
aroNC

S15A Fig

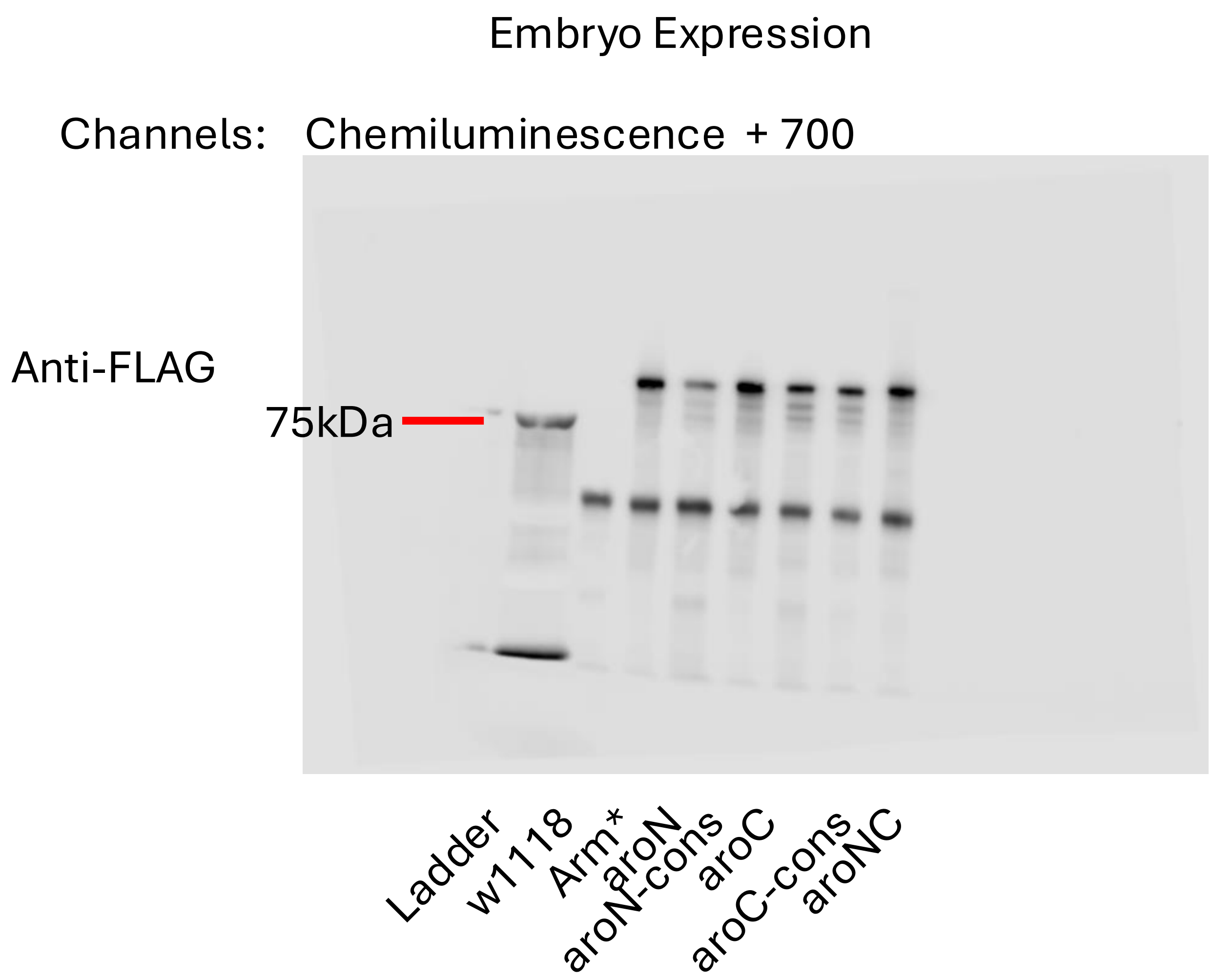
Embryo Expression
Channels: Chemiluminescence + 700
Anti-FLAG
75kDa
Ladder
w1118
Arm*
aroN
aroN-cons
aroC
aroC-cons
aroNC

S15A Fig

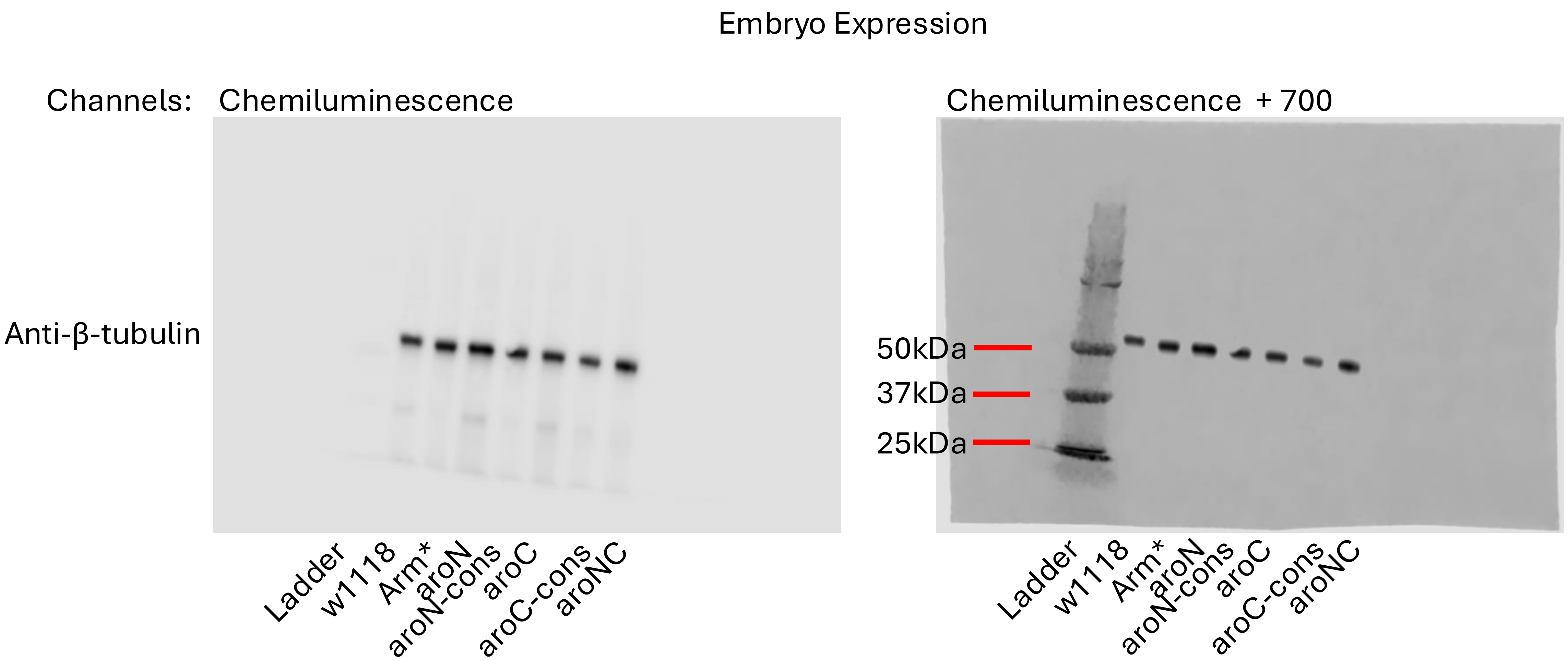
Embryo Expression
Channels:
Chemiluminescence
Chemiluminescence + 700
Anti-β-tubulin
50kDa
37kDa
25kDa
Ladder
w1118
Arm*
aroN
aroN-cons
aroC
aroC-cons
aroNC
Ladder
w1118
Arm*
aroN
aroN-cons
aroC
aroC-cons
aroNC

S18B Fig

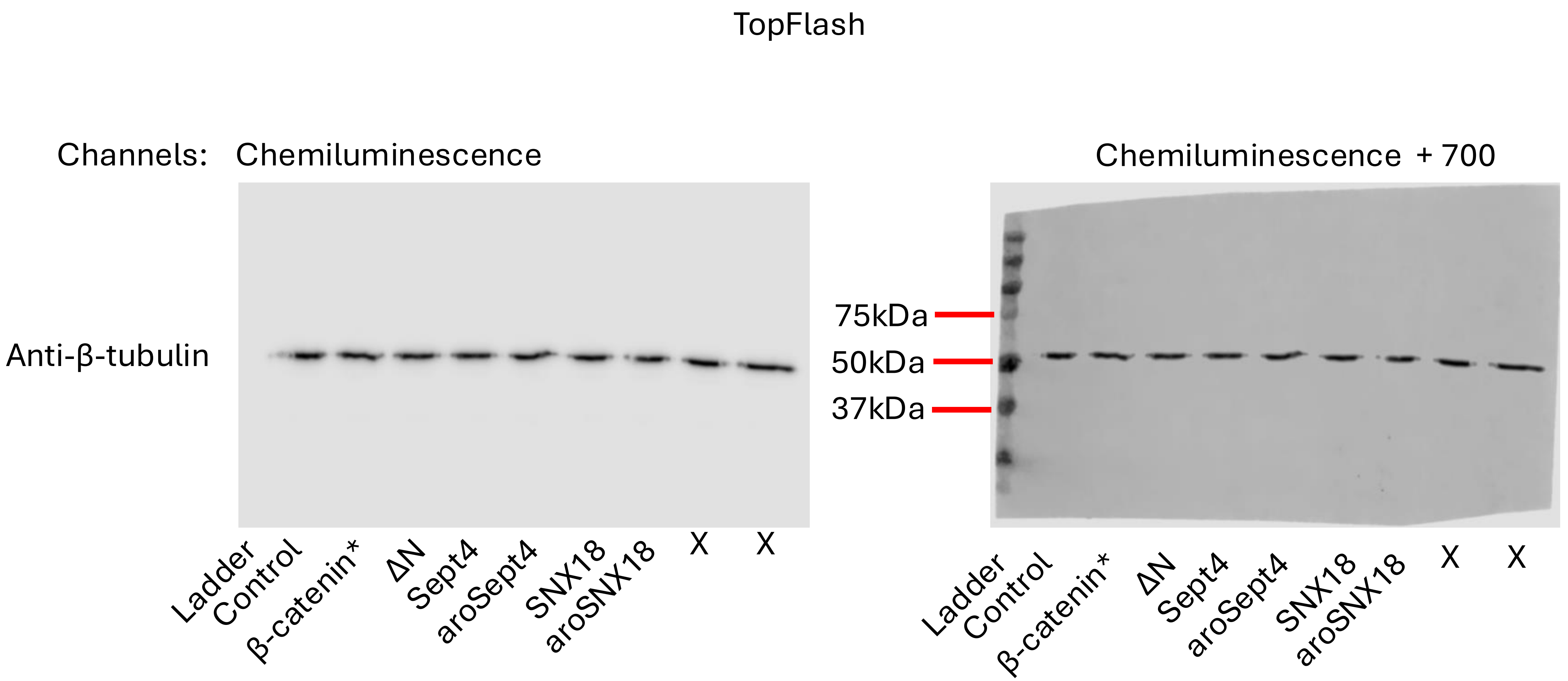
TopFlash
Channels:
Chemiluminescence
Chemiluminescence + 700
Anti-β-tubulin
75kDa
50kDa
37kDa
Ladder
Control
β-catenin*
ΔN
Sept4
aroSept4
SNX18
aroSNX18
X
X
Ladder
Control
β-catenin*
ΔN
Sept4
aroSept4
SNX18
aroSNX18
X
X

S18B Fig

TopFlash

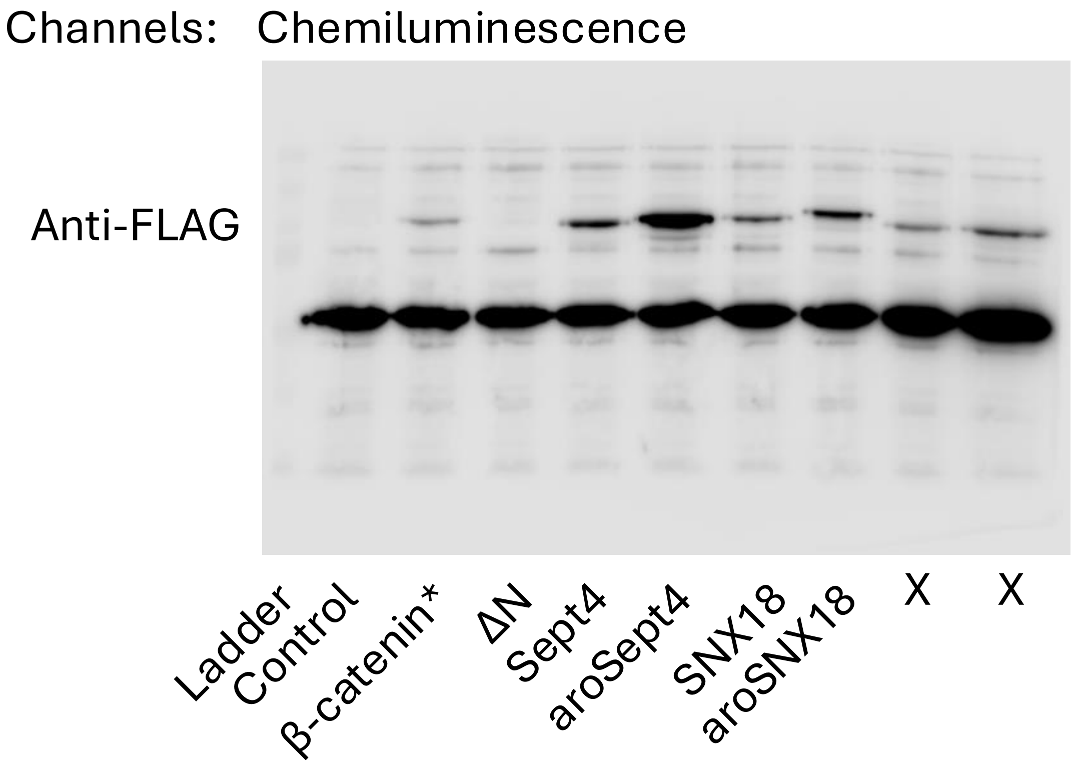

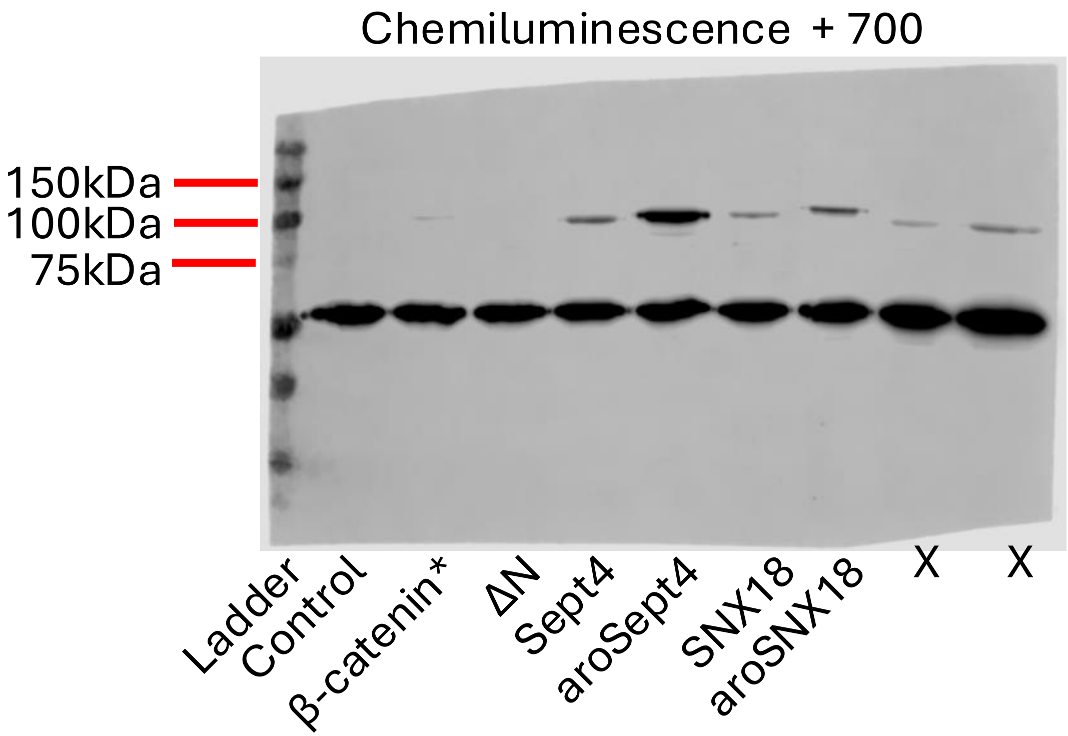

S18D Fig

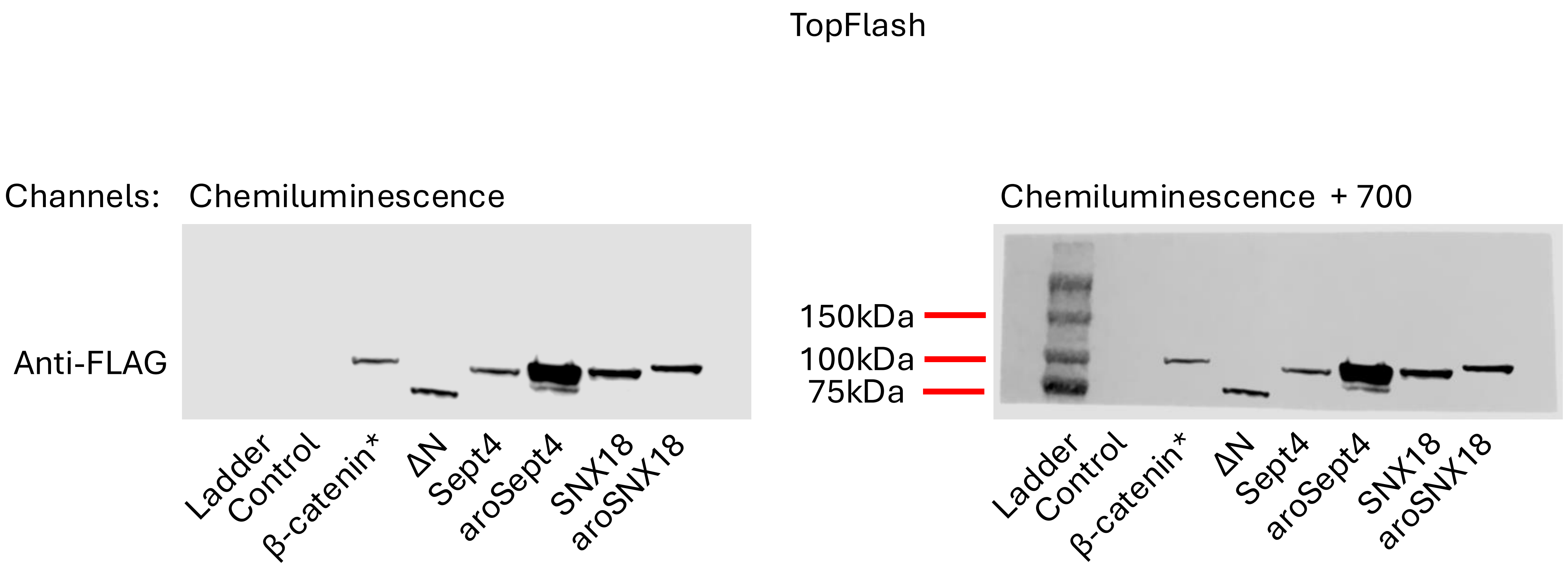
TopFlash
Channels:
Chemiluminescence
Anti-FLAG
Ladder
Control
β-catenin*
ΔN
Sept4
aroSept4
SNX18
aroSNX18
Chemiluminescence + 700
150kDa
100kDa
75kDa
Ladder
Control
β-catenin*
ΔN
Sept4
aroSept4
SNX18
aroSNX18

S18D Fig

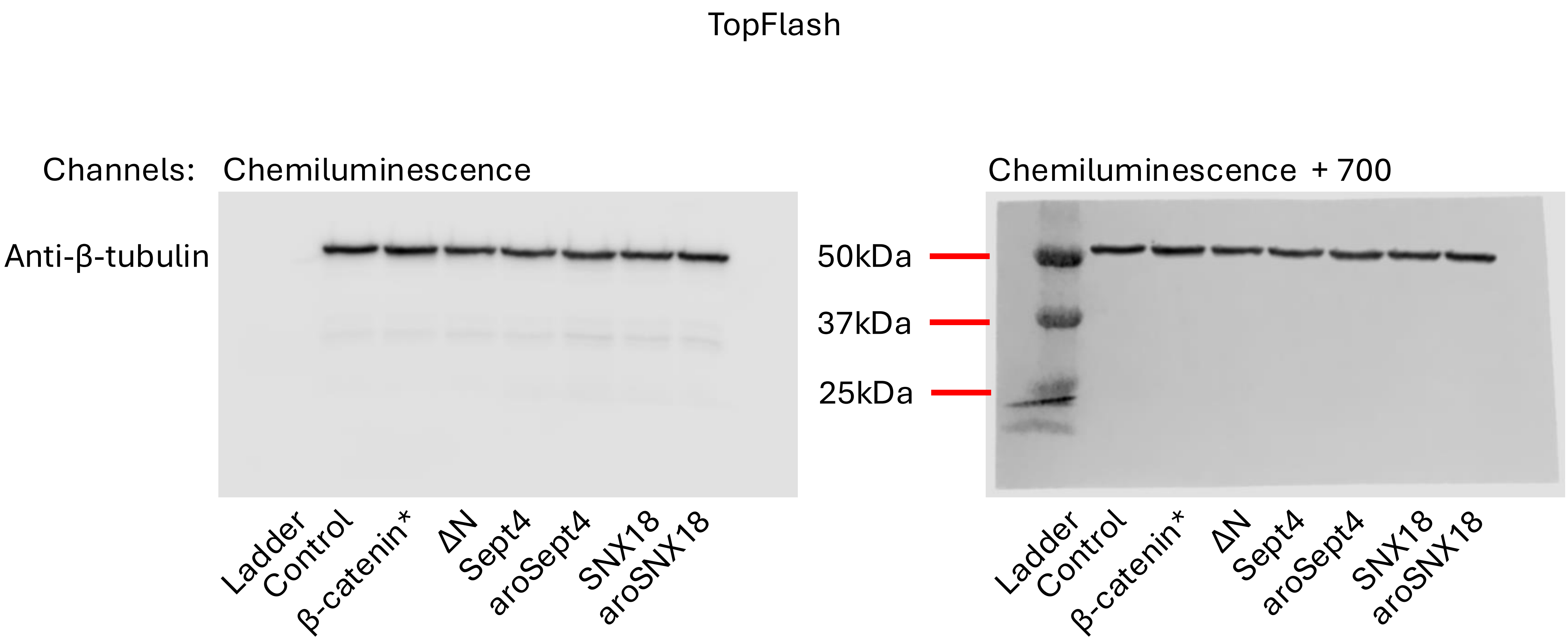
TopFlash
Channels:
Chemiluminescence
Chemiluminescence + 700
Anti-β-tubulin
50kDa
37kDa
25kDa
Ladder
Control
β-catenin*
ΔN
Sept4
aroSept4
SNX18
aroSNX18
Ladder
Control
β-catenin*
ΔN
Sept4
aroSept4
SNX18
aroSNX18
