# Supplementary material for: Wnt target gene activation requires β-catenin separation into biomolecular condensates
Source: PLoS Biol. 2024 Sep 24;22(9):e3002368. doi: 10.1371/journal.pbio.3002368 (PMC11460698; doi:10.1371/journal.pbio.3002368)
Supplement: Attachment — Submitted filename: Response to reviewers_PBIOLOGY_D_23_02482R1.pdf [file pbio.3002368.s023.pdf]

Point by point responses to reviewers' critiques (in blue)

Executive summary of changes to the figures - The following additions and modifications have been done to the figures:

- We have added a new Figure (Fig 3)
- Figure 4 (previous Fig 3) is largely redone (panels A, B, E, F)
- Previous Fig. 4 is replaced with new data (Fig 5)
- Added panel B to Fig. 7 (formerly Fig. 6)
- Added several panels to Fig. 8 (formerly Fig. 7)
- Fig. 10 (formerly Fig. 9) is redone with additional SNX-β-catenin mutant

Supplemental Figures S3, S5, S7, S8, S10, S11 and S17 are new.

In addition, we have included three papers that were published in late 2023 that are relevant to our story:

Zhao et al (2023) LEF1 enhances β-catenin transactivation through IDR-dependent liquid-liquid phase separation. Life Science Alliance 6(11): e202302118.
Goldman et al (2023) Intrinsically disordered domain of transcription factor TCF-1 is required for T cell developmental fidelity. Nature Immunology 24: 1698-1710.
Gui et al (2023) Targeted perturbation of signaling-driven condensates. Molecular Cell 83: 4141-4157.

All three are strong papers that complement our work. For example we extend the data in Zhao et al and Goldman et al on the role of IDRs in TCFs by demonstating that aromatic residues in LEF1's IDR are required for its ability to co-condense with β-catenin. While Gui et al contains interesting data on peptide inhibitors of TCF/β-catenin condensates, the caveat that these inhibitors block the pathway by non-condensate mechanisms was not (in our opinion) carefully addressed. This is not a criticism, but rather reflects the challenge of rigorously demonstrating that condensation of proteins are required for physiological function, which our manuscript (now greatly improved by addressing reviewer concerns) addresses for β-catenin.

Reviewers' comments

Rev. 1:
In this study, Stewart et al. shed light on the molecular requirements and functional roles of biomolecular condensate formation of the transcriptional co-factor β-catenin in the nucleus. While earlier studies pinpointed a role of the terminal intrinsically disordered regions (IDRs), how IDR their roles in mediating protein-protein interactions and/or driving condensates separately contribute to transcriptional regulation has not been addressed. The authors show that aromatic residues in both the N- and C-terminal IDR of β-catenin are important for condensate formation. As proof for the physiological relevance of biomolecular condensation of

β-catenin, the authors propose that these aromatic residues are crucial for β-catenin's transcriptional role in the nucleus, while nuclear import of the protein is not affected. In addition, the authors elegantly show rescue of β-catenin condensation and transcriptional activity by replacement of its IDRs with aromatic-rich, phase separation-promoting IDRs from two unrelated proteins, strongly supporting a functional role of condensate formation. Interestingly, in vivo experiments in Drosophila indicate that the degree of dependency on β-catenin condensate formation for the activation of Wnt targets is different for different tissues, thus revealing tissue context dependency.

Overall, the manuscript is well-written and provides a clear description of the advances and limitations of the work compared to previous studies. In general, the presented work provides a very valuable contribution to the field of Wnt signaling and biomolecular condensation. Several points however will need to be addressed to validate some of the conclusions and strengthen the manuscript.

We appreciate the kind words of this reviewer but take their concerns seriously.

Major points:

1) The authors attempt to discriminate different aromatic β-catenin mutants with regard to their biomolecular condensation and transcriptional output. However, for many experiments quantifications of β-catenin levels are lacking. As formation of biomolecular condensates is highly dependent on protein concentration (e.g. figure 1B compare 3 vs 4 uM), it will be critical to obtain quantitative measurement of protein expression levels in the comparison of different mutants.
For instance, in figure 3A-B, the authors observe a transcriptional difference between β-catenin AroN compared to AroC and AroNC, while claiming β-catenin is expressed 'at similar levels'. Based on the shown western blot, however, aroN clearly displays higher expression levels compared to the other variants, which could explain the relatively higher transcriptional activity of this aroN mutant. Another example is shown in figure 4B, where clear differences in expression are observed upon Dox-induction, contrasting the conclusions in the text.

To address this concern, the data for Fig. 4A, Fig. 4E (new, formerly figure 3), Figure 5B and C (formerly Fig 4B) and Figure 10A (formerly Fig. 9A) were redone, and this time the westerns were done in triplicate and the quantification is shown. While the main conclusions of these panels are unchanged, these improvements emphasize that the differences in signaling activity with the various mutants are not due to expression levels.

There is a bothersome variability from experiment to experiment in the western signal of our transfected β-catenin variants, making it difficult to get expression matched data for the figures. The result is that each of the experiments have each been done many times. Interestingly, while it has been difficult to get conditions where the western signal for each construct looks identical, the reporter data much more consistent. We are extremely confident that in these assays, the differences in signaling are intrinsic to each β-catenin mutant, and the differences

are not driven by subtle differences in expression. For example, aroN is always higher than aroC in numerous (>15) experiments. Regarding Fig. 10B, the western and quantification is not perfect, so we have included two additional versions of the experiment in S17 Fig, to illustrate that the reporter data is consistent, i.e., aroSept4 and aroSNX18 are always show minimal activation of the TopFlash reporter, even when expressed at higher levels according to the western.

Similarly, most fluorescent images are not quantified. For example, in figure 4C aroN appears more highly expressed compared to other clones. Additionally, quantification of foci in this figure would be helpful, as in the current images differences in foci formation are not obvious and do not match the conclusions in the text. AroC and AroNC are claimed to not form any condensates, however small foci are visible in two out of the three images. In summary, the authors should perform quantifications for all western blots and immunofluorescent images (except for in vitro data) and weaken their claims regarding minor differences between the different β-catenin mutants, unless substantiated by equal expression levels or statistical tests.

We agree that the quality of the micrographs in the previous Fig. 4 were not sufficient. We have repeated these experiments with GFP tagged versions of the various β-catenins. These new data (Fig. 3A) reveal a consistent and dramatic difference between β-catenin* (which form puncta in the nucleus) and aroN, aroC and aroNC which do not. Quantification of the puncta/cell is also included (Fig. 3B).

These data demonstrate that the aromatics are important for the condensation of β-catenin and confirm the β-catenin-LEF1 colocalization in a recently published paper (Zhao et al 2023) which we comment on in the results (lines 219-232) and discussion (lines 522-542). We also note in the results and discussion that the different assays (in vitro droplet, GFP puncta in cells, Wnt readouts) do not perfectly align (lines 215-218; lines 612-616). We think these differences reflect the complexities and limitations of each assay, e.g., we don't believe that the large β-catenin* puncta in Fig. 3 represent all the condensates in the cell: just the most obvious ones. Understanding the limitations for these experiments will require further investigation, but the GFP-fusion cell culture data bolster our main conclusion that β-catenin condensation is driven by aromatic residues in the terminal IDRs.

2) To support the claim that the aromatic residues in the N-terminal IDR mainly serve to promote condensate formation required for transcriptional regulation, it would be helpful to show that the interactome of the relevant β-catenin mutants is not altered, or at least show that known interactions are not significantly altered upon mutation of the aromatic N-terminal IDR residues.

The review raises a critical point: how do we know that mutating the aromatic residues in the IDR doesn't interrupt binding of β-catenin to its partners. We addressed this concern by performing co-IPs against known β-catenin binding partners. We observed no consistent difference in the co-IP of β-catenin* and aroN with TCF7, TCF7L2 (both of which binds to Arm

repeats 3-8), BRG1 (binding to Arm repeats 9-12) and p300 and CBP (the published binding data is more complicated, but most reports find the strongest binding to the C-terminal IDR). Strikingly, similar data were also obtained comparing β-catenin* and aroNC. These data are presented in S8 Fig and discussed in the results (lines 254-262) and discussion (lines 489-490). These data suggest that the aromatic mutants do not globally disrupt the protein-protein interactions that can be detected in a co-IP (where the concentrations of the proteins are low due to dilution of the extract).

We thank the reviewer for inspiring these experiments, which emphasize that the aromatic residue-driven condensation we are studying is distinct from the traditional model of β-catenin acting as an adaptor between TCFs and co-activators.

3) In Figure 3C the authors claim that nuclear import of β-catenin is not affected by aromatic mutations disrupting its biomolecular condensation. Given the novelty of this finding, the authors should substantiate this finding by repeating this experiment in other cell lines to ensure the generalizability of this observation. In addition, as stated above, quantitative measurements are required to determine the nuclear β-catenin fraction for the different mutants.

We agree that the ability of the aromatic mutants to enter the nucleus is a key point, as this indicates a defect in a nuclear function. We fractionated the cell extracts into a cytosolic and nuclear fraction in HEK293T cells and found that nuclear levels of aroNC were similar to β-catenin* (Fig. 4E,F). We note that the levels of nuclear β-catenin in these experiments are very low compared to the IF data (Fig. 4C,D). We suspect that β-catenin is exported out of the nuclei during the fractionation (despite our efforts to fractionate quickly and at low temperatures). To extend these data to another cell line, fractionation was performed on the Dox-inducible Hela lines used in Fig. 5 (S11 Fig). Again, no obvious difference was detected between β-catenin* and the aromatic mutants.

4) The authors observe clear differences between SP5 and AXIN2 transcription upon overexpression the different β-catenin mutants in Figure 4A. These results are intriguing, but puzzling. To gain better insight in the different transcriptomes of various β-catenin mutants, RNA sequencing analysis would be helpful. Alternatively, at least some of the canonical Wnt target genes, such as LGR5, CCND1 and MYC should be included in the analysis.

We agree that RNAseq will be required to fully understand the varying responses of Wnt targets to the aromatic mutants, but we fell this is beyond the scope of this manuscript. In repeating the qRT-PCR experiments, we included Notum (CCND1 and MYC were not activated under the conditions we used). These data, along with quantified westerns are now shown in Fig. 5. The results demonstrate that mutation of the aromatic residues compromises the ability of β-

catenin to activate endogenous Wnt targets, but to varying degrees. But aroNC is dramatically compromised in activation of SP5, AXIN2 and Notum.

5) To determine the effects of the different β-catenin mutants on transcription, the authors use a doxycycline-inducible expression system. Bearing in mind the concentration-dependent formation of biomolecular condensates, it may be hypothesized that β-catenin-mediated transcription is only induced once the critical concentration for BMC formation is reached, thereby acting as an on/off switch for transcription. Can the authors use their inducible system to titrate doxycycline and determine whether indeed transcription is activated in a binary manner, or whether the response to increasing expression levels is linear?

We agree that this would be an informative experiment, but there is a surprising level of cell-cell variation in the expression of β-catenin in our DOX-inducible lines (which are pools, not clonal). Perhaps with assays detecting expression of Wnt readouts in individual cells we could address whether their activation is binary or graded, but this is beyond the scope of the current manuscript.

6) Figure 4C, 3B: Both on western blot and in IF, the aroN mutant appears more stable. Potentially, the aromatic mutations in the N-terminal tail may affect β-catenin degradation, for instance by allosterically disrupting its N-terminal recognition, phosphorylation, or ubiquitination. Can the authors clarify their view on this?

This is an interesting idea that is actively discussed in our group. When transiently expressed in the S33Y background (which should prevent destruction complex mediated degradation) there are no clear-cut difference in expression levels (though some of the authors feel that the aromatic mutants are consistently expressed at a higher level). But when wild-type β-catenin is compared with an aroNC mutant (with Serine at position 33) we see a dramatic increase in expression levels in HEK293T (shown in Fig. S3). We suspect that the aromatic mutants compromise the ability of β-catenin to be recognized and modified by the destruction complex, but confirming this would require additional experiments. For this report, we briefly discuss this result in the discussion (lines 634-638), linking it to recent data from others that the destructive complex utilizes a condensate-driven mechanism to degrade β-catenin.

7) Based on the results shown in Figure 5 and 6, where similar defects are observed for the aroN/C vs aroN/C conserved mutants, the authors conclude that the conserved aromatic residues are most important for β-catenin's function. However, in Figure S6 mutations with lower conservation (sticker and spacer mutants) also show significant signaling defects. This could suggest that mutating several aromatics could already be sufficient to compromise condensate formation. To substantiate the statement that the conserved residues are more important than the non-conserved residues, these mutants should be tested side-by-side in one of the functional assays at comparable expression levels.

We agree that we overstated our results. The original impetus for mutating the conserved aromatics was to reduce the overall number of mutations and still abrogate signaling. In this sense, aroNcon and aroCcon were successful in Drosophila readouts, which we now state more specifically in the text. But we agree that additional mutants would be needed to strengthen this suggestion, which we now state (lines 560-561).

Our data indicate that condensation is driven by multiple aromatic residues, and further mutagenesis will be required to parse out whether some aromatic residues are more important than others. One interesting addition to the revised manuscript is our finding that if the 7 tyrosine residues in the IDRs are altered to phenylalanines, there is no detectable difference in signaling activity (Fig. S10B). This suggests that it is the overall aromaticity of these residues that are important, rather than the hydroxyl groups on the tyrosines.

Minor points:

1) To draw an easy comparison with previous work, protein concentrations should be mentioned above the microscopy images in Figure 1B, 2C-E. DONE, though we do not consider the absolute concentrations to be important, due to prep to prep variation. In all our in vitro droplet experiments, the proteins being tested are purified simultaneously, and carefully compared on SDS-PAGE for consistency. The qualitative differences between various proteins are the main point.

2) In Figure 2H and S4C, condensates containing both LEF1 and deltaNC are shown, whereas in Figure 2E at the same concentration (middle panel) there are no condensates detected. Can the authors clarify this point? There are small LEF1 condensates in Fig. 2E - they might be difficult to see on different computer screens). We don't see a reproducible difference in LEF1 condensates when ΔNC is added. This is in contrast to β-catenin*, which does have a significant effect on LEF1 condensation (now shown in Fig. S5C and D).

3) Convert figure 4C and 5D to greyscale, in support of the color blind. Figure 4C is no longer part of the manuscript and the former Fig 5D (now 6D) was converted to greyscale.

4) In Figure 9C and D the control and β-catenin* should be included as well. DONE; these experiments were repeated at 29˚C (where Gal4 is often more active) during the critical developmental phase (3rd instar, early pupation). We obtained a more dramatic reduction in pigmentation in GMR::Arm* and GMR::Sept4 eyes compared to the original figure (done at 25C). This reduction in pigmentation was not observed when the aromatic mutants in the Sept4 IDR were mutated. We thank the reviewer for helping us to improve this important data.

Rev. 2:

In this manuscript, Stewart and colleagues demonstrated that aromatic amino acids of beta-catenin positioned in IDRs play a crucial role in BMC of itself using in vitro assays. Then, authors showed that these aromatic amino acids are important in forming BMC droplet with Lef-1 IDR, and this beta-catenin-Lef-1 BMC greatly accelerates Wnt signaling. Therefore, authors confirmed their findings biologically using fly models including eye development, Wnt reporter activity in wing disk, and body segmentation. The highlight of this study is that IDRs of other proteins,

which are independent to Wnt signaling but are known to be BMC protein can function as well as N-IDR of beta-catenin in Wnt signal activation.

Those data authors presented here are interesting and will give us additional insights to understand how Wnt signaling is regulated by BMC. The statistical analysis, supplementary information, and data availability for this manuscript appear to be of a sufficient level. However, this manuscript in current form can be insufficient for publication in terms of experimental design, data collection, and paper writing, as described below.

We hope the point-by-point responses below convey the seriousness with which we have taken the reviewer's comments.

Major points;

1. Overall, this manuscript requires effort and time to read. Because Figure legends contain detailed results, but the main text outlines the methodology and results. Therefore, it is necessary for understanding to go back and forth among the main text, figures, and legends frequently. Authors should explain why the experiments were selected, how the experiments were performed and what results were obtained in a way that can be understood just by reading the main text. The legend should include only explanations of the figures themselves but should not include explanations results of experiments. Please detail the results of experiments in the Results section.

We regret that the original version of our work was a difficult read. We have expanded the results in many places to make it more readable, explaining the rationale and results of each figure, taking care to make it stand alone from the figure legends.

2. Authors substituted all aromatic amino acids of beta-catenin IDRs into alanine to avoid BMC, resulting in Wnt signal downregulation. One of these aromatic amino acids, tyrosine is known to be not only important in BMC, but also the amino acid phosphorylated. Therefore, authors should show that the level of tyrosine phosphorylation of beta-catenin is not affected by the elimination of tyrosines. Authors can show this point by beta-catenin IP-Phospho tyrosine IB experiment. In addition, substitution of all those IDR tyrosines into other aromatic amino acids like F or W for TopFlash reporter assay may support the present data.

The reviewer raises an important point, as it is known that phosphorylation of β-catenin on specific tyrosine residues can significantly elevate its signaling activity. We have addressed this concern as the reviewer suggested. Changing all the Y residues to F did not alter the signaling activity of β-catenin* (S10 Fig). In addition, a co-IP of β-catenin* and aroNC did not reveal any detectable difference in the phospho Tyr status of the protein (S10 Fig). We thank the reviewer for bringing up this important point. The importance of Tyr phosphorylation in β-catenin signaling activity is briefly discussed in the Results (lines 292-300) and Discussion (lines 565-568).

3. As described in the Discussion section, authors introduced S33Y mutation to stabilize beta-

catenin for focusing the transcriptional activity by beta-catenin-Lef-1 BMC. Of course, we know this substitution is sometimes used for beta-catenin stabilization. However, it seems strange to employ the S-Y substitution in this study. Because authors eliminated aromatic amino acids including tyrosine for BMC but introduced another "aromatic Y" in same IDR for protein stabilization. I wondered why authors did not introduce S33A mutation as in many other studies and in this study (Y-A, F-A, W-A). Authors should show 'no difference' in droplet formation and TopFlash assays between S33Y- and S33A-based beta-catenin mutants.

We agree that is was unfortunate to use S33Y for the stabilizing mutation. To address this, we purified recombinant wild-type β-catenin and aroNC with serine at position 33 in vitro (along with their S33Y counterparts and performed condensate assays (S3A Fig). In multiple assays with multiple preparations, no detectable difference was seen between S33Y and wild-type (S33) or S33Y-aroNC and S33-aroNC. In a topflash assay where wild-type beta-catenin activated the reporter 10-15 fold, aroNC displayed dramatically reduced activation of the reporter (S3B Fig).

We also note that the stabilizing mutants used to make Arm* in *Drosophila* (T52A and S56A) confirm the main conclusion of the manuscript, i.e., aromatic residues in the terminal IDRs are required for condensation and signaling activity.

4. In Fig. 2, authors introduced substitutions of aromatic amino acids only in beta-catenin IDRs to confirming BMC mimicking interaction between beta-catenin and Lef-1. Author should additionally show the results when aromatic substitutions are introduced in Lef-1 IDR.

In Fig. 3 of the revised manuscript, we show that expression of mCherry-LEF1 co-localizes with β-catenin in puncta in cultured cells. We then identified 14 aromatic residues in the region between LEF1's β-catenin binding domain and the DNA binding HMG domain. While this aroLEF1 mutant localizes to the nucleus, it does not co-localize with β-catenin puncta (Figs. 3C & S7). This data extends the recent findings of others (Zhao et al 2023; Goldman et al 2023) that show that the central IDR of LEF1 and TCF7/TCF1 are required for condensation and transcriptional regulation.

5. Authors showed the co-localization/interaction of beta-catenin and Lef-1 in BMC mimicking droplet in vitro in Fig. 2C and localization of beta-catenin in nucleus in Fig. 3C. Authors should show the colocalization of beta-catenin and Lef-1 in puncta observed in nucleus.

New data in Figs. 3C and S7 show co-localization of GFP-β-catenin and mCherry-LEF1 in nuclei of human cells.

6. In Fig. 3C and D, authors compared the level of nuclear beta-catenin by immunofluorescent experiments. Authors should show more quantitative data. The level of nuclear beta-catenin can be evaluated by WB experiment with anti-beta-catenin after the separation of nuclear fraction.

New cell fractionation data in Fig. 4E,F show that aroNC accumulates in the nucleus of HEK293T cell extracts to a similar extent as β-catenin*. Similar data is also provided for Hela cells in S11 Fig.

7. Authors showed in Fig. 9A that IDRs of Sept4 and SNX18 can be functionally replaced with N-IDR of beta-catenin and loss of aromatic amino acids of Sept4 impaired beta-catenin function in Wnt signaling. Please include the results also with the loss of aromatic amino acids of SNX18 IDR.

We generated a SNX18-β-catenin fusion where all 13 aromatic residues in the heterologous SNX18 IDR are changed to alanines. As shown in Fig. 10B and S16 Fig, this protein (aroSNX18) has greatly reduced signaling activity compared to SNX18.

8. Authors can combine Fig. 6 with 7.

We choose not to do this because it would shrink the images too much.

Minor points;
1. In the legend of Fig. 1. We could not see the indication of (B). DONE
2. In Fig. 7E, Da::Arm* should be Da::arm*. DONE
3. Authors did not describe anything for Fig. 8C in the main text. DONE (line 446).

I fell that all of the above is required for the publication in PLoS Biology except Major point #8.

Rev. 3: Mark Peifer – note that this reviewer has signed his review.
Transcriptional regulation by the Wnt/beta-catenin pathway is key to development and tissue homeostasis and is disrupted in disease. Many transcription factors contain intrinsically disordered regions that can mediate phase separation in vitro, but the roles of these in vivo remain less clear, as few in vivo studies have taken apart the IDRs and assessed function. Here the Cadigan lab builds on an earlier observation that beta-catenin can phase separate in vitro and in vivo and that began assessment of the roles of aromatic residues. They substantially extend these earlier observations—characterizing in detail the roles of both the N-terminal and C-terminal IDRs and of the conserved and less conserved aromatic residues in each, using multiple assays. In an impressive series of experiments, they assess phase separation in vitro, transcriptional activity on reporter genes and on endogenous targets in mammalian cells, and, most impressive, signaling activity in vivo in several contexts in Drosophila. The data are carefully quantified and support their conclusions. These data lead to a much more nuanced picture and provide one of the most complete analyses of the IDRs of a transcription factor in vivo. This work will be of broad interests to cell and

developmental biologists. I had some suggestions about clarity and a few questions about the data presented that should be straightforward to address.

We appreciate the encouraging words and insightful critiques.

1. There was one important deficit in the discussion of prior work. Many of the aromatic residues in the N-terminal IDR (and 6 of 8 conserved aromatic residues) are deleted in the ArmS10 mutant, which retains strong signaling activity-- many of the authors' effects on genuine in vivo targets support the idea that such a mutant should retain significant activity. It's also worth noting that the same paper suggests ArmΔN, deleting all N-terminal amino acids, is less active than ArmS10. In earlier work from the same lab, the authors found that ArmS14, which deletes the last two conserved aromatic acids in the N-terminal domain (those not deleted by ArmS10) is fully active in signaling in vivo.

An important fact that was left out of the original manuscript is that our ΔN deletion proteins (Δ 2-146 for Arm; Δ1-140 for human β-catenin) have significant signaling activity in the fly embryonic epidermis and wing imaginal discs. They have minimal signaling activity in the fly eye and cell culture reporter assays, making these readouts ideal for the heterologous IDR rescue experiments shown in Fig. 10. We have now included a paragraph in the Discussion (lines 576-589) describing this and relating this information to previous deletions made in flies and in mammals. Regarding the published fly deletions the ΔN described in Pai et al 1996; 1997 (Δ1-128) has significant signaling activity in the fly embryonic epidermis but significantly less than mutant S10 (Δ34-87; removing W35, Y40, F71, F77 & F81). Mutant S14, (Δ101-139; removes F106 & F121) has near wild-type signaling activity in the embryonic epidermis (Orsulic and Peifer 1996). We think these results are consistent with a model where the number of aromatics in the deletions positively correlate with signaling activity. We also note that aroN (removing all nine N-terminal aromatics) has significant signaling activity in the embryonic epidermis.

The data in mouse developmental genetics (exon 3 deletions remove residues 5-80 containing six of the nine aromatics) and other deletions in human cancer lines fit a similar pattern. We don't understand why different Wnt readouts react differently to the loss of aromatics and N-IDR deletions and we don't believe that the aromatics are the only residues influencing condensation. We are pursuing these important questions with additional experiments.

2. Fig. 2B vc C. Did beta-catenin enhance droplet formation by Lef1. It appears so, and if so this is worth noting.

Yes, we consistently see this in the in vitro droplet assay and supplemental Fig. 5C,D documents this effect. We also show in cultured cells that mCh1-LEF1 only exhibits large droplets when co-expressed with GFP-β-catenin (Fig. 3C).

3. The authors should tell us in the main text the nature of the amino acid change(s) in Arm* and define in the text how aroN and aroC differ from aroNcon and aroC-cons

This is now explained more clearly in the Results (lines 337-343)

4. The authors say: "All the aromatic Arm mutants (which contain the stabilizing mutants of Arm*) exhibit minimal signaling activity in this assay". This needs to be more nuanced since they actually show they have low but statistically significant signaling activity.

Agreed. These results are now explained more carefully in the Results (lines 358-359)

5. The authors should move Fig S8A into the main figure as it provides important information.

DONE

6. The assay is Figure 7 is the most interesting of all of their assays, as it integrates effects on all target genes in vivo. It provides important information about ArmNC function. Did they test any of their other mutants in the Fig. 7 assay?

We have included the additional aromatic mutants in a revised Fig. 8 (formerly Fig. 7). These mutants rescue the cuticle defect of zygotic arm null to a similar degree as aroNC.

7. Fig 8. It is worth noting that that aroSept4 did rescue spherical condensates, though not at the same concentration

We agree this is worth noting and now include a brief comment on this in the Results (lines 446-447).

8. The chimeras remove the residues that are involved in beta-catenin phosphorylation and targeting for destruction—this needs to be noted in these experiments.

We agree this is worth noting and now include a brief comment on this in the Results (lines 461-463).

9. The authors stated "The finding that substitution of specific residues in the N-IDR (or deletion of the N-IDR) severely compromised BMC formation and signaling activity"—this needs to be nuanced to make it clear this was only true IN VITRO.

We have modified the text (line 494) but also include our new data that aroN in cells do not form detectable puncta.

Minor issues

Fig S1 It is worth noting that AlphaFold predicts alpha-helical regions in the N-terminal IDR (one of which is the alpha-catenin binding site)

We agree this is worth noting and now include a brief comment on this in the Results (lines 139-141) noting that these α-helices are lower confidence than those in the Arm repeats.

Rev. 4:

In the manuscript titeled "Beta-catenin-mediated activation of Wnt target genes utilizs a biomolecular condensate-dependent mechanism" the authors explore the contribution of beta-catenin phase-separation to it's capacity to drive transcription. The authors perform elegant and detailed mutational analysis and use in vitro, cell culture and fly in vivo models to evaluate the contribution of aromatic amino acids to beta-catenin condensate formation and transactivation. These findings are timely and build upon earlier work by Zamudio et al., as noted by the authors. This work supports a link between condensate formation and active transcription, but also inform that there is condensate-independent transactivation by beta-catenin. The work is generally of high quality and provides novel insights in condensate formation and transcriptional activation. I only have one minor comment.

We appreciate that the reviewer appreciates our work.

Fig 1. How does the S33Y affect condensate formation? Could it be the explanation of the difference between Zamudio et al and this report? It adds one aromatic Y to the IDR, therefore the aroN is not fully mutated. In light of the aromatic IDR substitution data, and the reduced activity of Arm with as little as 5 aromatic substitutions, this could be plausible explanation. Can the authors test a S33C or S33P oncogenic mutant and see if the residual in-vitro droplet formation is maintained?

To address this, we purified recombinant wild-type β-catenin and aroNC with serine at position 33 in vitro (along with their S33Y counterparts and performed condensate assays (S3A). In multiple assays with multiple preparations, no detectable difference was seen between S33Y and wild-type (S33) or S33Y-aroNC and S33-aroNC. These results are described in the Results (lines 164-165; 242-243).
