# Supplementary material for: Wnt target gene activation requires β-catenin separation into biomolecular condensates
Source: PLoS Biol. 2024 Sep 24;22(9):e3002368. doi: 10.1371/journal.pbio.3002368 (PMC11460698; doi:10.1371/journal.pbio.3002368)
Supplement: Attachment — Submitted filename: Point by point to PLOS Biology Reviewer 2 -revision 2.docx [file pbio.3002368.s024.docx]

Point by point responses to reviewer 2's concerns (in blue)

The authors responded sincerely to many of the issues raised and provided numerous pieces of evidence to support the story. In this revised format, almost all the data except one was of a high enough standard to be published. Now the paper excellently presented important new findings. We appreciate these positive comments (and those of reviewers 1 and 3)

However, it seems that only the data on the localization of nuclear β-catenin may still be confusing to readers. In this manuscript, Fig. 3A and B show that nuclear BMC puncta are observed only when IDRs are intact, but when IDRs are lost, BMC puncta are not formed. In these panels, almost no signal of β-catenin itself that has lost IDRs is observed in the nucleus surrounded by white dashes despite the presence of nuclear β-catenin(aroN/C) protein showing in other figures. Why?

The images in Fig. 3A were acquired with the goal of highlighting the nuclear puncta seen with GFP-β-catenin*, which are not observed when the aromatic residues in either terminal IDR are substituted. But we understand how the images could confuse readers in relation to the other data in Figs. 3 and 4 addressing the nuclear localization of β-catenins. As pointed out in the quantification in Fig. 3B, about 40% of the cells expressing GFP-β-catenin* have puncta. The remaining cells have diffuse signal in the cytosol and nucleus, similar in appearance to the GFP-aromatic mutants. We now include a supplemental figure (S7 Fig) with representative micrographs of cells expressing GFP-β-catenin* (but with no puncta) and the aromatic mutants. The gain of these images is higher than those in Fig. 3A, clearly showing considerable GFP signal in the nucleus and cytosol. Several panels also show untransfected cells, which have no detectable green fluorescence, demonstrating that the signal we observe is due to the GFP-β-catenin proteins. For convenience, we include S7 Fig below. A sentence referring to this figure was added to the results (page 8/9).

On the other hand, Fig. 3C and Fig. 4E clearly show by imaging and biochemistry that the presence or absence of IDRs does not affect the nuclear localization of β-catenin, strongly supporting the authors' claims. In addition, Fig. 4D also shows that the nuclear localization of β-catenin is not affected by IDRs. Fig. 4D also quantitatively shows that the nuclear localization of β-catenin is not affected by the presence or absence of IDRs, and therefore supports 3C and 4E, since the same amount of β-catenin is present in the nucleus regardless of the presence or absence of IDRs.

The data in S7 Fig. are consistent with our other results demonstrating that the aromatic mutants still accumulate in the nucleus. The reviewer mentions the data in Fig. 3C, where co-expression of the GFP-β-catenins with mCh1-LEF1 results in a dramatic shift in nuclear accumulation of β-catenin. The ability of LEF1 to promote nuclear β-catenin localization is well known, and we added a sentence commenting on this in the discussion (page 20) along with two references (Behrens et al 1996; Morgan et al 2019) to support our statement.

However, in this figure (4C), which is the basis for Fig. 4D, the BMC puncta formation in β-catenin* is unclear, making it difficult to make a qualitative assessment. Is it possible to adjust the contrast of the image a little more so that only β-catenin* clearly forms BMC puncta with same amount of nuclear β-catenin proteins?

The reviewer is correct that there is a lack of obvious puncta in the immunofluorescent images in Fig. 4D. These images were acquired at a lower magnification so we could efficiently quantify the signal of a large number of cells, to address whether the aromatic mutant signals had a different nuclear/cytosolic ratio than β-catenin (as shown in Fig. 4E, quantification showed no significant difference). We include higher magnification images in S9 Fig where some puncta are observed in cells expressing β-catenin* but not in the aromatic mutants. A sentence pointing out the difference between the images with GFP-β-catenin and FLAG immunoflorescence is now included in the Results (page 10).

Regarding this last point, we are beginning to utilize super-resolution imaging to better understand the GFP-β-catenin-mCherry-LEF1 puncta (as well as other TCF family members). I personally believe that the functional β-catenin/TCF puncta that we should be looking at are very small and difficult to visualize, but supporting this idea requires much more work and collaboration with imaging experts. Addressing this point is beyond the scope of our current manuscript.

All except for the problem above are consistent, and I believe this is an excellent paper worthy of acceptance. Congratulations. Thanks!

Changes to the References - We included the two references referred to above (refs 73, 74) and discovered that one reference in the text was not included in the previous list (ref 78). Two references in the previous list that are not cited in the manuscript have been removed, and the last reference (ref 94 in the revised manuscript) was incorrect and now has been corrected. We apologize for these previous oversights.
